# Author Correction: Genome editing in plants using CRISPR type I-D nuclease

**DOI:** 10.1038/s42003-021-01771-5

**Published:** 2021-02-25

**Authors:** Keishi Osakabe, Naoki Wada, Tomoko Miyaji, Emi Murakami, Kazuya Marui, Risa Ueta, Ryosuke Hashimoto, Chihiro Abe-Hara, Bihe Kong, Kentaro Yano, Yuriko Osakabe

**Affiliations:** 1 Graduate School of Technology, Industrial and Social Sciences, Tokushima University, Tokushima, 770-8503 Japan; 2 Department of Life Sciences, School of Agriculture, Meiji University, Kanagawa, 214-8571 Japan

**Keywords:** Molecular engineering in plants, Molecular engineering in plants

Correction to: *Communications Biology* 10.1038/s42003-020-01366-6, published online 6 November 2020.

The original version of the Article contained an error in Fig. 1a in which elements were missing from the depiction of the gRNA. The original (incorrect) figure is reproduced below. The error has been corrected in the HTML and PDF versions of the Article.

**Fig. 1:**
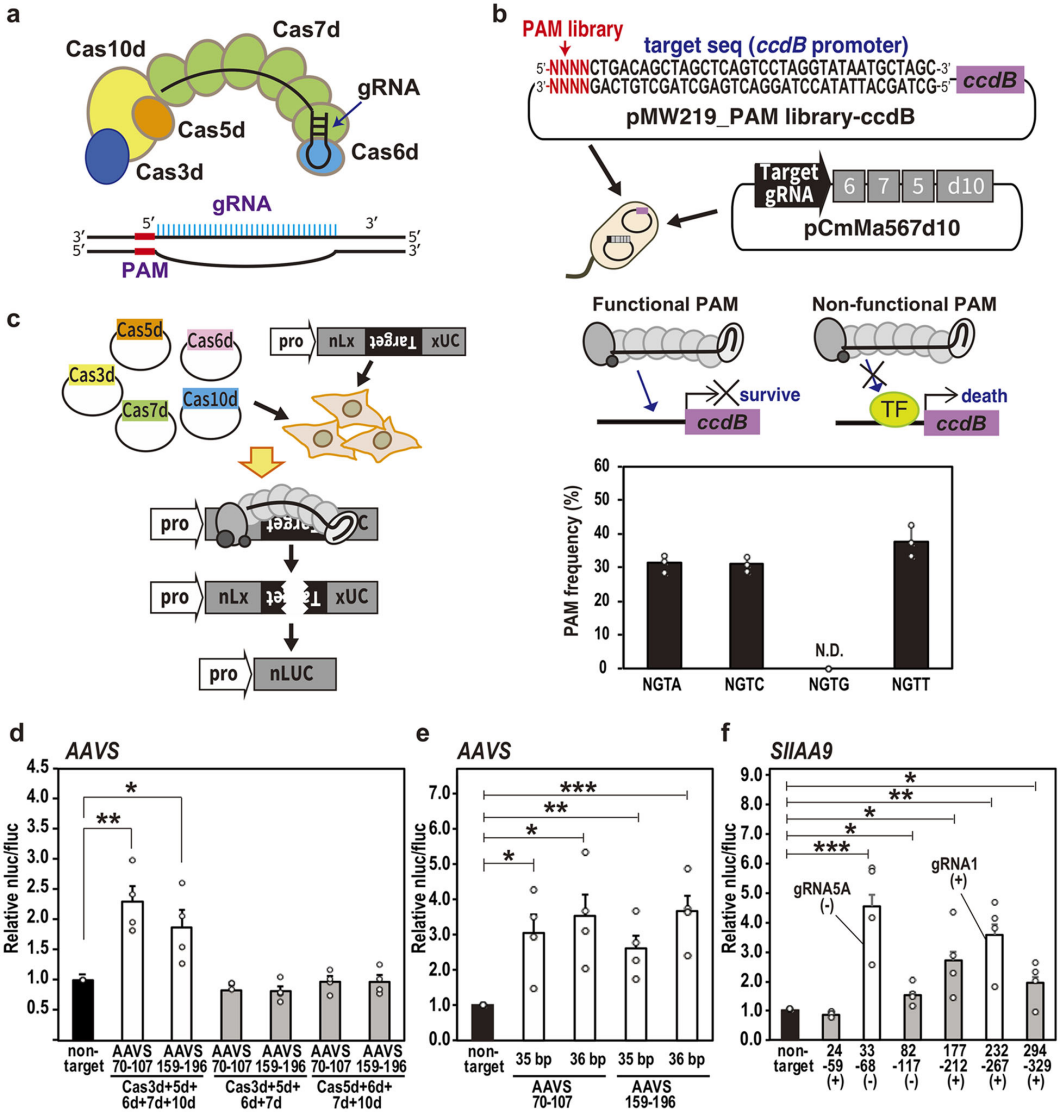
Genome editing activity of CRISPR type I-D detected by luc reporter assay.

