# Supplementary material for: Genome editing in plants using CRISPR type I-D nuclease
Source: Commun Biol. 2020 Nov 6;3:648. doi: 10.1038/s42003-020-01366-6 (PMC7648086; doi:10.1038/s42003-020-01366-6)
Supplement: Supplementary file 1 — Supplementary Information [file 42003_2020_1366_MOESM1_ESM.pdf]

Supplementary Information

Genome editing in plants using CRISPR type I-D nuclease

Keishi Osakabe[1*], Naoki Wada[1], Tomoko Miyaji[1], Emi Murakami[1], Kazuya Marui[1], Risa Ueta[1], Ryosuke Hashimoto[1], Chihiro Abe-Hara[1], Bihe Kong[2], Kentaro Yano[2], Yuriko Osakabe[1*]

[1]Graduate School of Technology, Industrial and Social Sciences, Tokushima University, Tokushima, 770-8503 Japan

[2] Department of Life Sciences, School of Agriculture, Meiji University, Kanagawa, 214-8571, Japan

***Correspondences**

Keishi Osakabe
Graduate School of Technology, Industrial and Social Sciences, Tokushima University, Tokushima, Japan
3-18-15 Kuramoto-cho, Tokushima-city, Tokushima 770-8503, JAPAN
Tel: +81-88- 634-6418 / Fax: +81-88- 634-6419
kosakabe@tokushima-u.ac.jp

Yuriko Osakabe
Graduate School of Technology, Industrial and Social Sciences, Tokushima University, Tokushima, Japan
2-1 Minami-josanjima, Tokushima-city, Tokushima 770-8503, JAPAN
Tel & Fax: +81-88- 656-9310
osakabe.yuriko@tokushima-u.ac.jp

Supplementary Figures

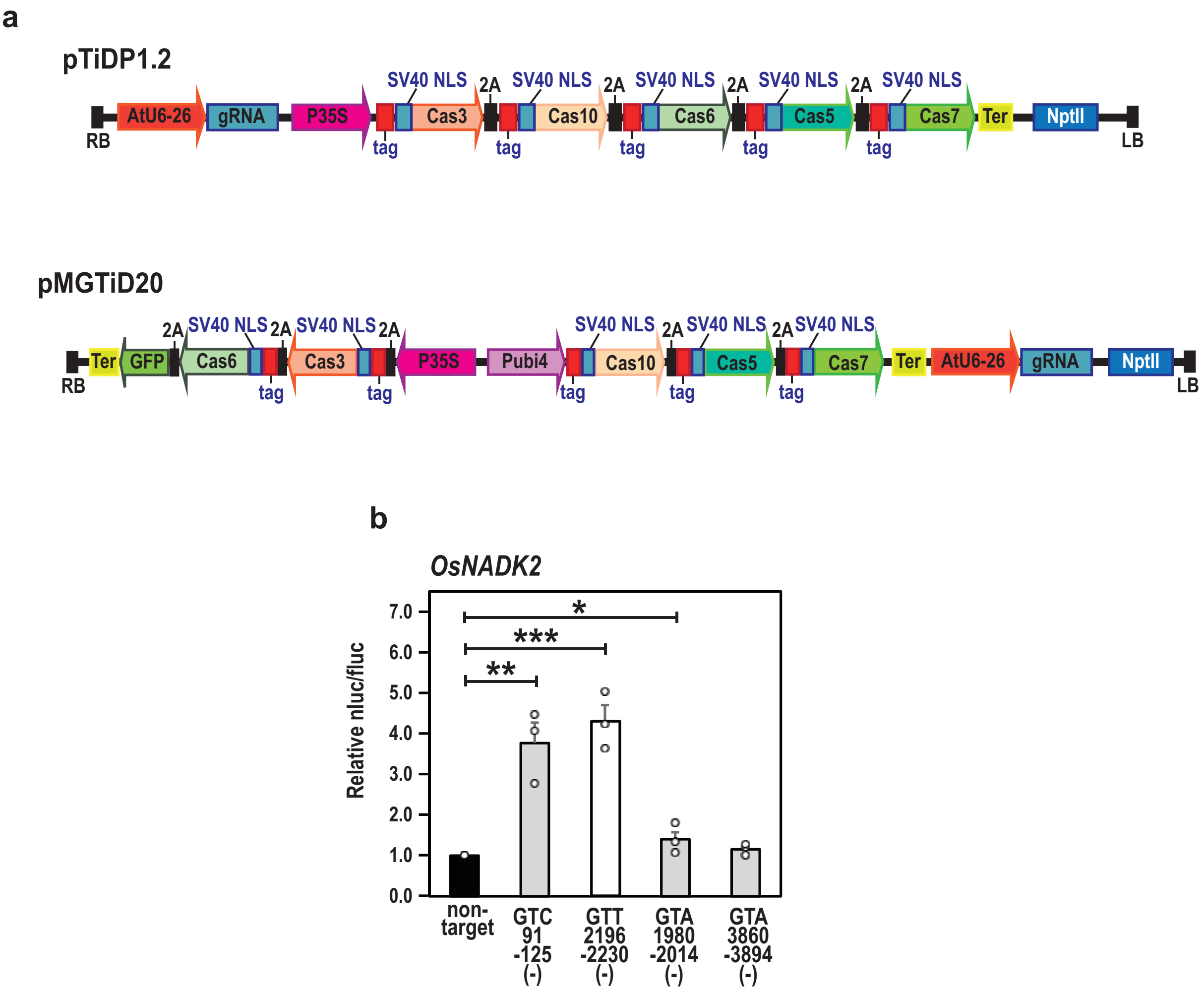

Supplementary Fig. 1. TiD expression vectors for plant cells and evaluation genome editing activity using Luc reporter assay.

a. Schematic structure of the Cas expression vector cassettes in tomato plants. SV40NLS on N-ter, which can be used fused with different tags, in Cas3d, Cas5d, Cas6d, Cas7d, and Cas10d were fused via 2A self-cleaving peptide to generate the single transcriptional products and express simultaneously. pTiDP1.2 was an all-in-one vector used in the *SlIAA9* mutagenesis and pMGTiD20, in which the two Cas expression cassettes were arranged in the same vector under the two promoters was used for the *SlRIN* mutagenesis. U6-26 gRNA: *Arabidopsis U6 snRNA-26* promoter and the gRNA sequence, 2x35SΩ: 2x *CaMV35S* promoter with the omega enhancer sequence, Pubi4: parsley *UBIQUITIN 4-2* promoter, AtCas9: *Arabidopsis*-codon optimized SpCas9, 2A: 2A self-cleaving peptide, NPTII: the kanamycin resistant marker expression cassette, RB: right border of T-DNA, LB: left border of T-DNA.

b. Luc reporter assay for the *OsNADK2* gene. gRNAs were target to the rice *NADK2* gene (*OsNADK2*) listed in **Supplementary Table 1**. Data are means ± S.E. independent experiments (n = 3) and *P < 0.05, **P < 0.01, and ***P < 0.005 are determined by Student's t tests.

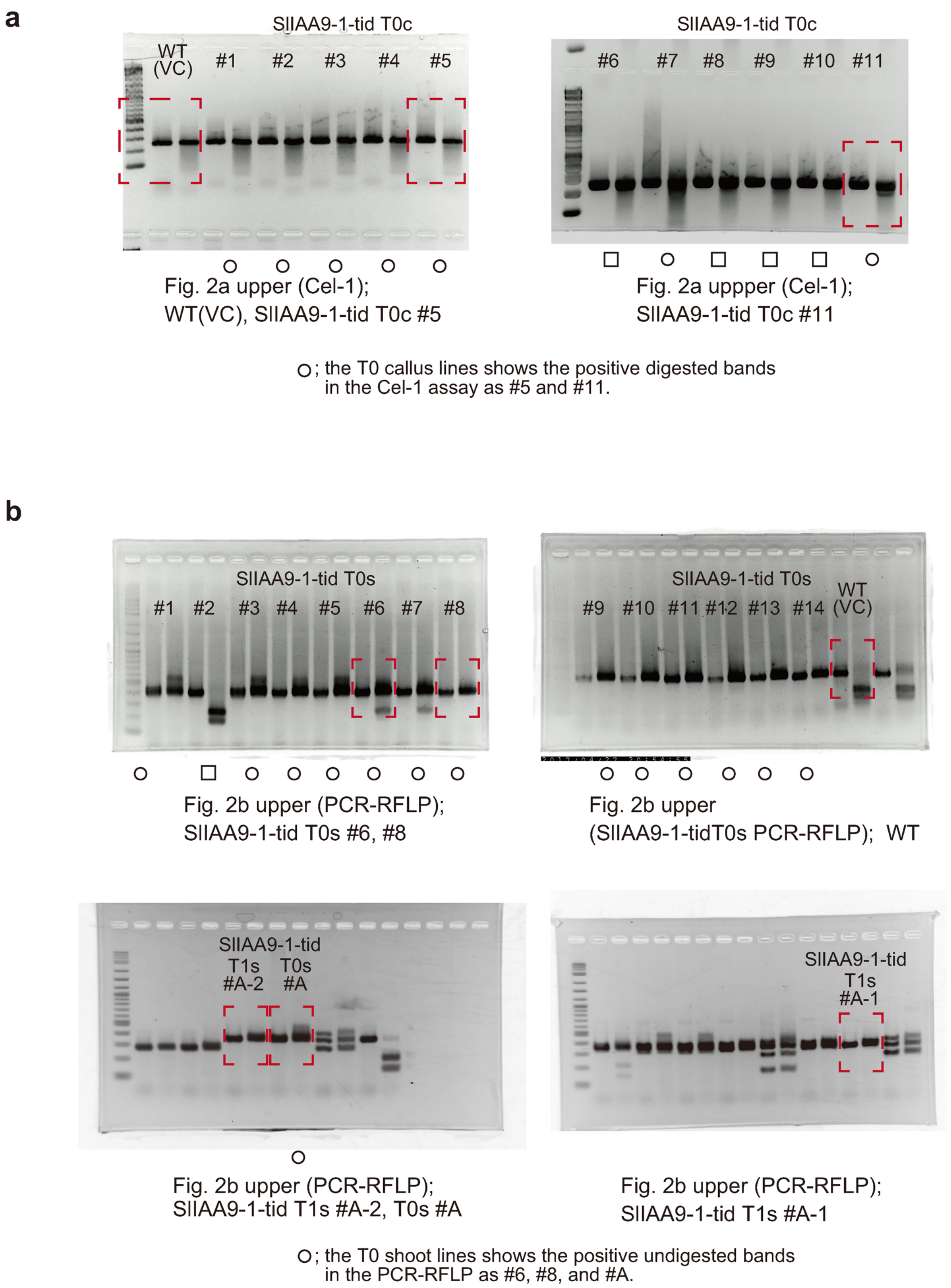

Supplementary Fig. 2. The mutations detected by Cel-1 assay in Micro-Tom calli and PCR-RFLP in Micro-Tom shoots.

a, #1 - 11; the CRISPR TiD transgenic Micro-Tom calli (T0 generation). **b,** #1 – 14 and #A; the CRISPR TiD transgenic Micro-Tom shoots (T0 generation), #A-1 and A-2; the next generations (T1) of #A. WT (VC); vector control. Gel images in red dashed boxes were used as representative data, as shown in **Fig. 2a** and **2b** upper panels.

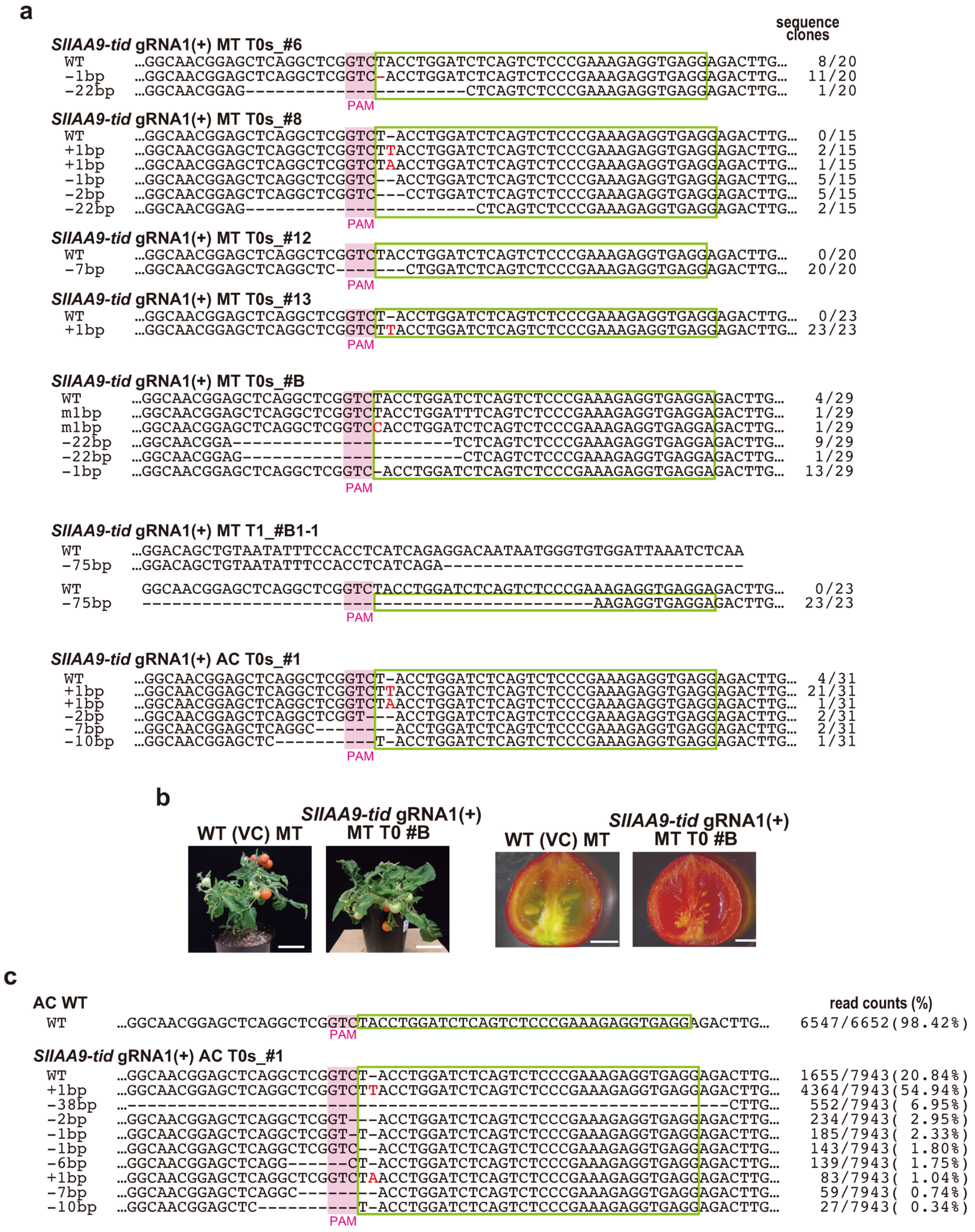

Supplementary Fig. 3. Small indels mutations for *SlIAA9* induced by CRISPR TiD.

a, Mutation sequences in the mutant shoots (T0 and T1 generations) transformed with CRISPR TiD analyzed by the Sanger method. WT; wild-type sequences. AC; Ailsa Craig. MT; Micro-Tom. gRNA target sequences are indicated in green boxes and PAM is indicated in pink boxes. The sequence frequencies in the cloned PCR products were indicated in the right of the sequence.

b, Plant phenotypes of *SlIAA9*-disrupted tomato plants and fruits with parthenocarpy phenotypes (Micro-Tom) generated by CRISPR TiD. Bars = 5 cm (left two panels), 1 cm (right two panels).

c, Mutation sequences in the *IAA9* gene of Ailsa Craig (AC) shoots (T0 generation) transformed with CRISPR TiD analyzed by amplicon deep sequencing using Mi-seq (illumina). WT; wild-type sequences. gRNA target sequences are indicated in green boxes and PAM is indicated in pink boxes. The sequence frequencies in the read counts in the deep sequencing are indicated to the right of the sequence.

a

mismatch numbers**	Number of target sites*									
	Arabidopsis ch01		Arabidopsis ch02		Arabidopsis ch03		Arabidopsis ch04		Arabidopsis ch05	
	SpCas9 (NGG)	MaTiD (GTH)	SpCas9 (NGG)	MaTiD (GTH)	SpCas9 (NGG)	MaTiD (GTH)	SpCas9 (NGG)	MaTiD (GTH)	SpCas9 (NGG)	MaTiD (GTH)
on-target	2024852	2518585	1332638	1633828	1609557	1946388	1267359	1549529	1811181	2239867
0	3590875	1524655	1527609	693787	2610265	919239	2152616	997440	2860629	1367336
1	6398057	2767420	2352226	1136182	3957375	1662319	3833721	1761886	4722706	2328135
2	10135564	4095706	3045817	1436936	4645525	2137870	5521159	2663123	6088907	3397776
3	17993819	5104391	5465023	1589495	7095499	2292971	9050160	3428185	9230475	4490174
4	49327434	5677644	22461231	1639245	26758664	2171542	26379806	3926023	32172304	5252813
5	231237832	6013954	136413816	1617877	161358929	1927721	135097225	4191087	188380784	5433103

*number of gRNA target sites for SpCas9 (PAM; -NGG) and MaTiD (PAM; -GTH) were counted.
**mismatch numbers in the gRNA target sequences.

b

mismatch numbers**	Number of target sites*									
	rice ch01		rice ch02		rice ch03		rice ch04		rice ch05	
	SpCas9 (NGG)	MaTiD (GTH)	SpCas9 (NGG)	MaTiD (GTH)	SpCas9 (NGG)	MaTiD (GTH)	SpCas9 (NGG)	MaTiD (GTH)	SpCas9 (NGG)	MaTiD (GTH)
on-target	4516525	3376576	3678198	2800248	3768183	2837691	3803566	2764410	3185462	2324640
0	12836488	3565284	7439644	2118542	5357006	1437160	19339544	5681897	13818736	4119728
1	15130824	4486074	9589678	2853940	7211042	2076294	19884580	6655072	13930719	4820324
2	17638958	5024562	11826850	3452532	9628199	2467548	18516740	6126554	13194104	4466932
3	29584075	5386008	20131036	3783027	18838555	2780419	23794277	5265181	18262170	3978810
4	86255454	5667482	58826862	3918574	59821057	3057810	56858637	4631118	47063963	3574805
5	357590050	5993240	239903685	3991907	251127268	3313017	224010317	4295631	184883233	3407192

mismatch numbers**	Number of target sites*									
	rice ch06		rice ch07		rice ch08		rice ch09		rice ch10	
	SpCas9 (NGG)	MaTiD (GTH)	SpCas9 (NGG)	MaTiD (GTH)	SpCas9 (NGG)	MaTiD (GTH)	SpCas9 (NGG)	MaTiD (GTH)	SpCas9 (NGG)	MaTiD (GTH)
on-target	3272682	2428549	3093651	2312690	2962775	2203515	2405969	1790241	2437615	1799198
0	10349726	3278980	10070412	3121744	10117802	3052558	7209264	2263984	6352007	2051120
1	11084119	3874278	11477590	3626120	11074802	3715202	7095090	2577298	6595022	2399723
2	11534563	3754940	11622940	3882958	11083564	3690172	6793294	2453110	6658671	2290609
3	17632542	3515342	15711031	3701227	15472612	3448383	9735400	2420985	9752114	2062108
4	48759249	3404396	40627798	3597676	38939686	3180426	24999200	2298920	26220540	1912004
5	195432187	3449701	162291552	3401642	151591982	3025592	98988240	2144257	104679914	1841915

mismatch numbers**	Number of target sites*			
	rice ch11		rice ch12	
	SpCas9 (NGG)	MaTiD (GTH)	SpCas9 (NGG)	MaTiD (GTH)
on-target	2964124	2257634	2832114	2135743
0	9289431	3455080	10194082	3450926
1	8562481	3130112	10938431	3956030
2	8786127	3155307	10736892	3893499
3	12672891	3144028	14528418	3638460
4	33632380	3074704	35427842	3343985
5	137599525	3085224	136000749	3131025

*number of gRNA target sites for SpCas9 (PAM; -NGG) and MaTiD (PAM; -GTH) were counted.
**mismatch numbers in the gRNA target sequences.

Supplementary Fig. 4. TiD target site numbers including mismatches in the Arabidopsis and rice whole genomes.

The target sites for SpCas9 (PAM; NGG) and MaTiD (PAM; GTH) were counted in the Arabidopsis (a) and rice (b) whole genomes using Cas-OFFinder and an in-house Perl script.

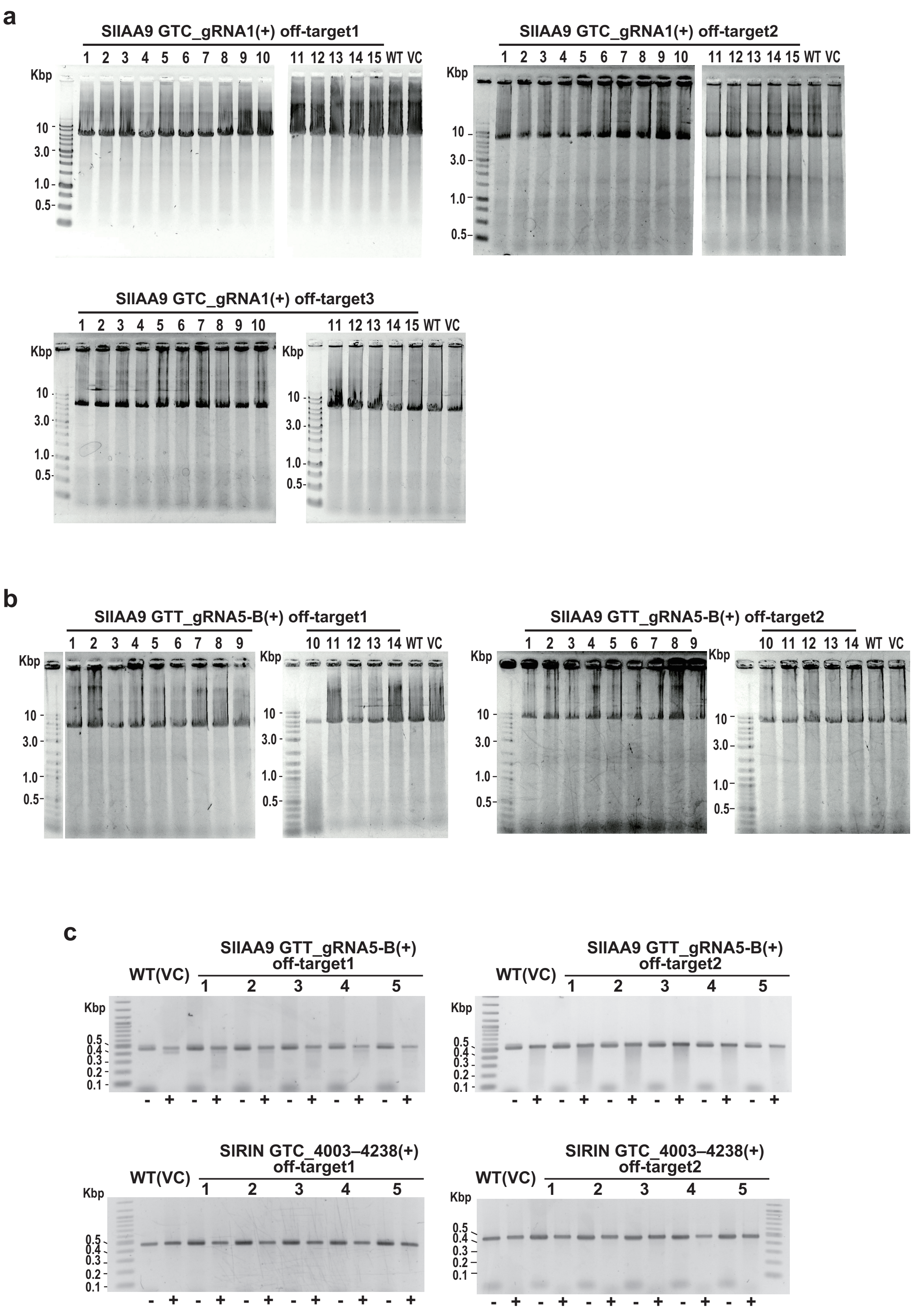

Supplementary Fig. 5. Detection of off-target sites using long-range PCR and small indels.

a, b, Long-range PCR at off-target sites for *SlIAA9* GTC_gRNA1(+) (**a**; off-target1 - 3) and *SlIAA9* GTT_gRNA5-B(+) (**b**; off-target1 and off-target2) in the transgenic T0 calli of Micro-Tom (*SlIAA9-tid_*

GTC_gRNA1(+)MT T0s_ #1-15, *SlIAA9-tid_* GTT_gRNA5-B(+)MT T0s_ #1-14), wild-type (WT) and vector control plants (VC).

c. The off-target effects of small indels were evaluated for *SlIAA9* GTT_gRNA5-B(+) and *SlRIN* GTC_4003–4238(+) in transgenic T0 calli by Cel-1 assay. The off-target effects for *SlIAA9* GTT_gRNA5-A(-) were not estimated because there are no sequences similar to the *SlIAA9* GTT_gRNA5-A(-) target sequence in the tomato genome.

The off-target candidate sequences are listed in **Supplementary Tables 5** and all primers for PCR for detecting off-target effects are listed in **Supplementary Tables 3 and 4**.

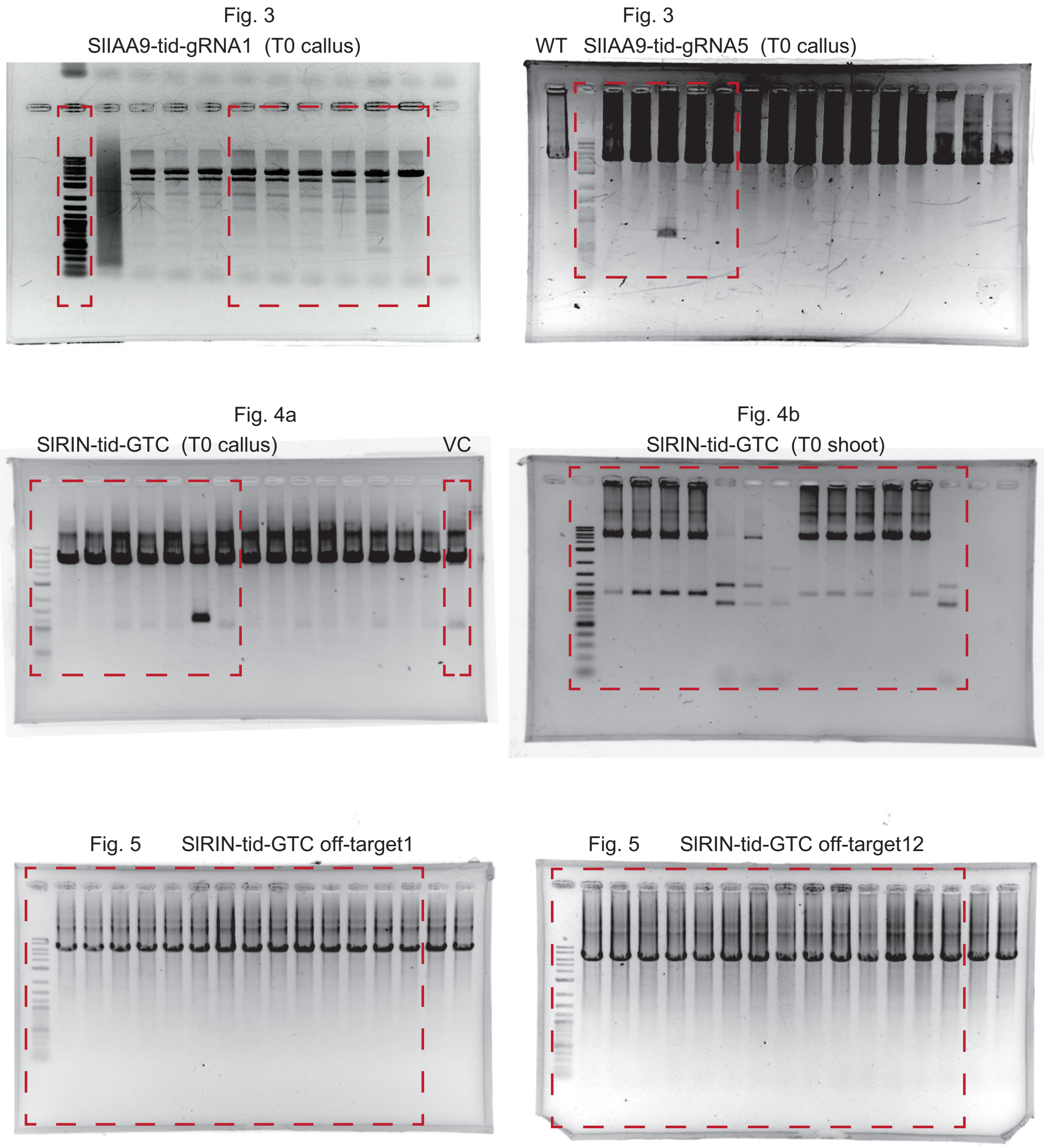

Supplementary Fig. 6. The mutations detected by long-range PCR in Micro-Tom calli and shoots. Gel images in red dashed boxes were used as representative data, as shown in **Fig. 3, Fig. 4a** and **4b**, and **Fig. 5d** lower panels.

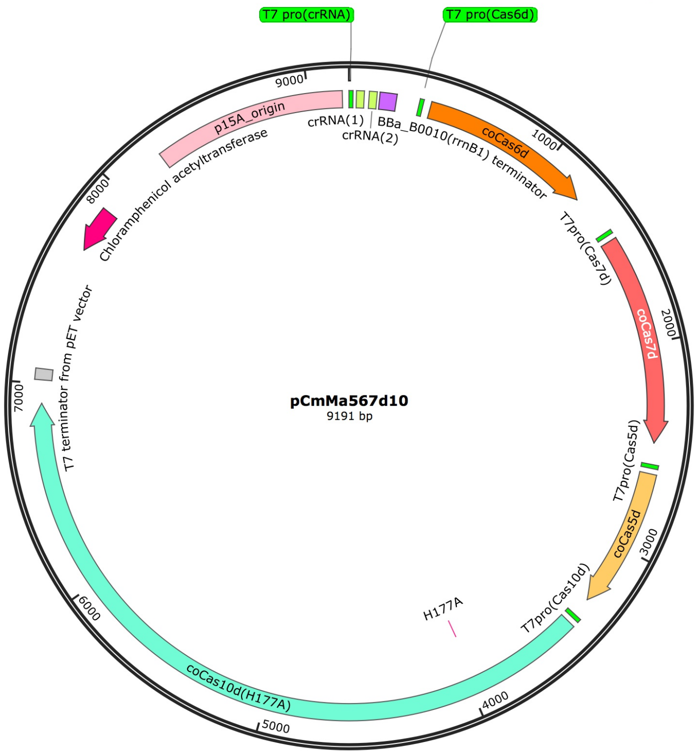

Supplementary Fig. 7. Plasmid vector pCmMa567d10.

1..19: T7 promoter
36..72: crRNA(1)
96..132: crRNA(2)
146..225: rrnB1 terminator
335..353: T7 promoter
390..1223: coCas6d
1430..1448T7 promoter
1475..2476: coCas7d
2579..2597: T7 promoter
2624..3298: coCas5d
3390..3408: T7 promoter
3435..6905: coCas10d
3963..3965: H177A
7017..7069: T7 terminator from pET vector
7671..7886 (complement): Chloramphenicol acetyltransferase gene
8248..9160: p15A origin

>pCmMa567d10
TAATACGACTCACTATAGGCTAGTGCTAGCACCTCGTTCCAATTAATCTTAAGCCCTATTAGGGATTGAAACGGAGACC
CTCAATTGTCGGTCTCGTTCCAATTAATCTTAAGCCCTATTAGGGATTGAAACTTTTTTTGTCGACCCAGGCATCAAATA
AAACGAAAGGCTCAGTCGAAAGACTGGGCCTTTCGTTTTATCTGTTGTTTGTCGGTGAACGCTCTCGTTGATTTTCCG
AACTTTAATGCGGTAGTTTATCACAGTTAAATTGCTAACGCAGTCAGGCACCGTGTATCGATCTCGAGCTTCGATCCGA
TAGACTAGCCGCTGGTAATAATACGACTCACTATAGGGAGAGATTTCTATTAGTACTTCATAAGGAGGACAGAATGCCG
TATAGCCTGGTGCTGAACCTGACCCCGCGCAGCCCGATTTATCCGAACTTCCTGACCGGCCGCCATCTGCATGCGCT
GTTCCTGACCCTGGTGAGCAGCGTGGATCAGGAACTGGGCAACATTCTGCATACCGCGGAAGCGGATAAAGCGTTC
ACCCTGAGCCCGCTGCAGATGCAGAGCGGCGGCAAAACCATTAACAGCCCGCAGTGGCGCCATGAACGCGAAATT
GCGAGCGAAACCCCGTGCTGGTGGCGCATTAGCCTGCTGGATGATCGCCTGTTCGGCAAACTGACCAGCCTGTGG
CTGAACCTGAACCCGAAACAGCCGTGGCATCTGGGCAGCGCGGATCTGGTGATTACCAGCGTGCTGGCGACCCCG
CAGAGCGTGCAGCCGTGGGCGAACAGCTGCACCTATCAGTATCTGTATGAAAACGCGAGCGAAACCAACCGCGAGT
TCGATTTCCTGTTCGCGACCCCGGTGACCTTCCGCCAGGGCAAATTCGATAGCGCGCTGCCGACCCGCGAACTGGT
GTTCAACAGCCTGCTGGGCCGCTGGAACCGCTATAGCGGCATTCCGTTCGATAGCATTGCGCTGGAAAGCATTTTCC
CGAGCTTCTTCGATATTCAGACCAAACTGGCGGATGAAGCGTATAAAAACCAGAGCATTGGCTGCGTGGGCGAAATT

CATTATCGCCTGCTGGGCGAAGTGGAACCGGCGAAAATTAAAGCGATTAACGCGCTGGCGGATTTCGCGCTGTATGC
GGGCGTGGGCCGCAAAACCACCATGGGCATGGGCATGACCCGCCGCATTAGCAAAGATAAACGCTAATAGCTTTCCT
GTGAGCAGCGAAAGAATTCCTAGCATAACCCCTTGGGGCCTCTAAACGGGTCTTGAGGGGTTTTTTGTTATACGCGA
GATAATCACTTGCATAGCTGCGTATGGAGGAAGCAACTCTTGAGTGTTAATATGTTGACCCCTGTATTAGGGATGCGG
GTAGTAGATCGGTCTGGATCCGCGTACTTCATTATGTATATTAATACGACTCACTATAGGGAGAAGATCTATAAAGGAGG
TAAATAATGACCTTCCTGACCAGCGTGGATGCGAAATTCTTCCATAGCGAAATTCCGTATAAACCGATGGGCAAATATG
TGCATTTCCTGACCATTCGCGTGACCGAAAGCTATCCGCTGTTCCAGACCGATGGCGAACTGAACAAAGCGCGCGT
GCGCGCGGGCATTGACAGCAAGAAAACCATTAGCCGCCTGAGCATGTTCAAACGCAAACAGAGCACCCCGGAACGC
CTGGTGGGCCGCGAACTGCTGCGCAACTATGGCCTGATTACCGCGGAAGAATGCGAATATAACGTGAAATTCGCGAT
GAACAACGCGGATTGCATTATTTATGGCTTCGCGATTGGCGATAGCGGCAGCGAAAAAAGCAAAGTGGTGGTGGATA
CCGCGTTCAGCATTACCCCGTTCGATGAAAGCCATGAGAGCTTCACCCTGAACGCGCCGTATGAAAACGGCACGATG
GCGAGCAAAGGCGAAAACAACACCAAAGTGGGCGAAGTGACCAGCCGCATTAACCAGCAGGATCATATTCGCCCGC
AGGTGTTCTTCCCGAGCATTGTGACCCTGAAAGATCCGACCGAAGCGAGCTTCCTGTATGTGTTCAACAACATTCTG
CGCACCCGCCATTATGGCGCGCAGACCACCCGCACCGGCCGCGTGCGCAACGAACTGATTGGCGTGATTTTCGCG
GATGGCGAAATTGTGAGCAACCTGCGCTGGACCCAGGCGATTTATGATCGCCTGCCGGATGAAGTGCTGCATAGCAT
TGATCCGCTGGATGAAGATCTGGTGATGGAAAAAGCGACCGAAGCGATTCAGGCGCTGATGGCGGAAGAATTTATTG
TGCATACCGATTTCATTGGCGAAAACTTCCAGCCGCTGCTGACCGAAGTGAAAACCCTGACCGGCACCGAAGCGGG
CATTCTGAGCGTGCTGGATCAGGCGAACAAAGAAAGCAAAAAATATTTCGAACAGTATATTGAAAAGAAAAAGGCGGA
AAAGAAATAAACGCGTGCTGCGTGGTCAAATGTGCGTAGACCAACCCCTTGCGGCCTCAATCGGGGGGGATGGGGT
TTTTTGTCGAGCTCGTCCTGTGTTAGCTGGAGGGTATAATACGACTCACTATAGGGAGAAAATTCATAAAGGAGGTAAA
TAATGGTGCATATTTATAGCTGCCAGCTGGAACTGCATGATAGCCTGTATTATGCGACCCGCGAAATTGGCCGCCTGTA
TGAAAGCGAACCGGTGATTCATAACTATGCGCTGTGCTATGCGCTGGGCCTGGTGAACAGCGATAGCTATCGCTATTT
CTGCAGCGAACAGATTCCGCAGTATCAGGAACATCTGAACCCGCTGAACGAAGAAAAAATTTATGTGACCCCGGCGC
GCGCGATTGCGCATACCGCGGTGCTGAACACCTGGAAATATGCGAACAACAACTATCATGTGGAAATGGAAAAAACC
CAGAAAAACATTCCGAGCTTCGGCCGCGCGAAAGAAATTGCGCCGGAAAGCATTTTCGAATGCTTCATTATTAGCCAT
CATCCGCTGCAGCTGCCGAAATGGATTCGCCTGGGCAAATGGATGAGCAAAGCGGAAGTGAAACTGACCGAACTGA
GCCTGAGCAAACAGAAAGAAGATCTGTTCATTTATCCGTATCCGCTGAACCCGCTGGATGTGATGTTCACCCATCAGG
TGATTGGCTATGATGTGATTAACATGCCGCCGGTGAGCCTGATTCGCAACGTGCGCATGCGCGGCGAATATTATCAGA
TTAGCGATCGCCCGGATCTGAAAATTCCGGCACGTCTGAGCTATCATTTCGGCTAATAGCTTACCTGGAGATCAAGGA
GATTACTCTAACCCCATCGGCCGTCTTAGGGGTTTTTTGTCAAGCTTGTCCTGTGTTAGCTGGAGGGTATAATACGAC
TCACTATAGGGAGACCCGGGATAAAGGAGGTAAATAATGCCTAAGAAGCAAAAGAAGCTGGAAGAAACCGGCCAACT
GAACCTGTTCGATAACACCACCGAAATCGATGATGAAGATCTGGATTTCGAGTTCGAAGATATCGATCTGGAATCCCT
GGTGTCCGAAGATCTGGGCATCACCGAATCCGTCTCCGATCGCCGCGTCGAAACCGTCCGCCAACTGCTGACCCTG
AAGCTGCTGCGCGAAGCCATCCGCGCCGAAAACCCTGATGATCGCGTCATGGCCGATTTCGCCGAAATGGTCCTGC
CTAACCTGCTGCGCCTGGCCATCGGCGTCACCGCCAAGGGCGGCAACTTCATCGAAGCCGTCGATCGCGGCCGCG
AACTGCGCAACAAGCCTAAGGCCAAGCGCGATAACGCCGGCGATCAATCCCTGAACACCCATCTGCTGAACGGCCT
GTTCCCTGCCAACCTGATCGAAAAGCGCCTGCAAAAGCTGAACACCACCGTCCGCCGCATCATCAAGGAGTTCGAA
CGCCGCCTGGCCATCGCCGGCTTCCTGGTCgccGATTTCGAAAAGTTCTCCTACGATCGCTTCCCTTCCATGTCCGAA
CGCTACATCCAAATCCAACGCGATTTCATCCAAGATCCTTTCAAGAACCAAGATCCTCGCAAGCTGTCCCGCGAAGAA
CATCGCGAAATCCTGCAAGTCCTGATCCCTGAACTGGGCCTGGATCGCTTCCTGTTCCCTGATAACCCTGAACGCTG
GCTGGAATACCTGGATGATCTGCTGTATATCGCCAAGAACACCCAACGCCGCAACGATACCGATCTGAACACCTCCG
AAGATGGCCTCAATGTCCGCCTGAACGATCGCGTCATCGAATCCCTGTGCGATCTGGCTTGCCTGGCTGATCGCCTG
GCCTCTATCATCAAGCATCCTCATGATGCCGAAAAGGCCTCCCTGCAGGACCTTCTGTATAGCCTGAGCGACGGTGA
GCTGAAATTCACCTATCATAGCATTGCGGAGAATCGTGGTGTGCTGACCAATGTGCTGAATAATGCGGTGATGGAGGC
GCATCAGGAGCTGGACTATCAGCCGCTGCTGTATCTGCCGACCGGTGTGGTGTATATTGCGCCGAAAAATGCGCCG
GAGGTGAGCCTGGAGACCCTGCCGAATCGTGTGGTGGACACCATTAAAAGCCTGTGCAGCGGTGAGCTGCAGCGT
AAACAGACCGGTTTCGGTCGTGACGGTAAAGGTATGAAATATGCGGACTATTATAGCCAGTTCTTCGACGACGCGGG
TCTGATGCGTGCGGCGCTGAATGCGACCCTGCGTATTCTGGGTGACAATAAAGCGAGCGTGGCGCGTAGCCGTGGT
GAGAATCTGATTAAATTCCAGCAGCAGGGTGTGCTGCCGACCGACTATGACTTCCATTGCGAGGACGACATTCGTATT
GACCGTCTGGCGGAGTTCGGTGACGTGGTGACCCGTAAAATTTGGGGTGACCGTCTGGAGAAAATTGAGCAGGCG
CGTAAACTGCAGAAAAACCTTCCGGCGCCGCCGGACCTGGACCTGATTAGCGAGATTGCGCATTATTGGAATCTGGA
GAATTATCTGCCGCAGATTCGTGCGATTAAACGTATTAATGAGAGCCTGAAAGAGCTGAAACTGAAAGGTAATACCGG
TGGTGTGCCGTATGAGTGGTATTATCTTGCCGCGCAGTATCTGAAACAGCATCCGGGTATTGAGGACATTCGTCCGGT
GGCGGAAGACCTGATTGCGTTCCTGGCAGCGAAAATTGCGGCGATTGTGGCGGGTTATAATCTGCCGGACGGTTGG
GAGGATCTGCGTGAGTGGGTGAATCAGGTGGTGCAGCTGCCGGGTCGTGAGCTGGCGCATAGCATTGAGACCTTC
CAAAAGGAACTGAATCATTATAATGCGGCGAAAAAACAGGGTCGTGGTCGTCAGCTGCTGTGCAGCATTAGCCATAG
CCCGTATAGCGTGAGCGAGCAGATGGAGAGCGCGGTGCTGTTCACCCCGCAGGTTTACACCAATAAACAGATGCTT
GCGGGTAGCAATGCGAAACGTAATATTAGCAGCATTGCGGGCACCGAGATGATGCTGCGTCAGATTCTGATGAATCA
GACCCAGGCGGTGGGTAAACGTTTCGAGGACGGTAAATATCGTTATCTGTATTTCTATCCGACCTATTATTTCACCCCG
GAGACCAATAGCTTCCTGCAGAAAGCGTATGCGAATATTGCGCAGACCCGTTTCGACAGCAGCATTAAACTGCATTTC

GTGGATAAAAATCTGGTGGCGAATTTCGACCGTACCCGTTATCAGAGCGTGGACAGCTTCCTGATTGACGAGAAACT
GCGTCAGAAAAAAGAGACCATTAATGAGGAGGAGGACGGTAAGAAAGACCGTACCTTCAAACTGAGCTATCCGGAG
GACAAGCCGCTGACCTTCTATTTCATGGCGCTGCCGCCGGGTCGTAATCCGACCGACACCGAGAGCTGGGTGATGC
CGGCGTGGCTGGGTCTGGCGTTCCCGATGATTCTGGACGTGAAAACCGTGGTGAGCGAGAGCCCGATTCCGCCGT
ATCGTGACGGTGCGGAGTTCGAGGAGACCGTGTTCCTGGACAGCGCGCCGCAGGCGATTCGTAGCCTGACCCGTT
GCGACCGTTTCCGTCTGGATCGCGTTCTGAATCCGTGGCAGGACAATGACGGCAAAAAGTATAGCGCGCCGCTGAA
TACCCTGACCGCGGCGTATAGCATTCACCTGGATGTGAATAGCAAACAGGGTAAAACCGGTTATGACCCGAATTGGG
GTAAACTGACGGAACTGGCGATTAATCTGGAGACCAGCCCGCTGTACGTGTTCCATTATTTCAAACAGTGGAAACGT
GGTAAAGACGCGGACATTCCGAGCGCGAATCGTATTGCCCTGTATCTGTATGACTTCTATCCGTGCTTCGACCCGTAT
GTGCAGGCGAATCGTACCAATCTGACCATTGACATGACCGCGGAGAGCCCGCTGAATCATCCGAAAAATCTGACCGA
GCTGTATCGTCAGTTCTATCGTGCGAAAAGCAGCAAAGGTAAACCGATTAAAGCGAATGCGATTCTGAAACCGATTGA
CGAGGCGGCGGACATTATTCTGAAAGCGGATAAAGCGATTAGCGACGACCTGACCAGCCTGGTGGCGGCGCGTCTG
TTCAAACTGATGGACCGTGTGCGTAGCCAGACCGCGGAGGGTCGTTATGTGATTAAAGAGCGTGACCAGGAGCGTG
AGAAAATTCTGGACTTCGCGAAATATTTCGTGAAAAATGTGTTCGAGGAGAGCTTCGAGAGCGACCGTGCGCGTCTG
GCGGGTCGTCAGCTGAATATTATTCGTGACACCTGCGAGTTCCTGTATCGTCTGGAGATGGACAAGGAGCGTCGTCA
GCGTCAGGTGCAGCCGCTGGACACCAGCAATAGCAGCAGCGAGGAGGAGGAGTAATCATGATTTCTTGTCGAACTG
GACAGTAGCAGAACCGCTAACGGGGGCGAAGGGGTTTTTTGTGGGTACCCAAAGGTGGTATACCAATGGTTACGTAA
AGACGTTTGTACATAACAAAGCCCGAAAGGAAGCTGAGTTGGCTGCTGCCACCGCTGAGCAATAACTAGCATAACCC
CTTGGGGCCTCTAAACGGGTCTTGAGGGGTTTTTTGAAGCCGGCGGCAGGCCTCGATATCGAATTTCTGCCATTCAT
CCGCTTATTATCACTTATTCAGGCGTAGCAACCAGGCGTTTAAGGGCACCAATAACTGCCTTAAAAAAATTACGCCCCG
CCCTGCCACTCATCGCAGTACTGTTGTAATTCATTAAGCATTCTGCCGACATGGAAGCCATCACAAACGGCATGATGA
ACCTGAATCGCCAGCGGCATCAGCACCTTGTCGCCTTGCGTATAATATTTGCCCATGGTGAAAACGGGGGCGAAGAA
GTTGTCCATATTGGCCACGTTTAAATCAAAACTGGTGAAACTCACCCAGGGATTGGCTGAGACGAAAAACATATTCTC
AATAAACCCTTTAGGGAAATAGGCCAGGTTTTCACCGTAACACGCCACATCTTGCGAATATATGTGTAGAAACTGCCG
GAAATCGTCGTGGTATTCACTCCAGAGCGATGAAAACGTTTCAGTTTGCTCATGGAAAACGGTGTAACAAGGGTGAA
CACTATCCCATATCACCAGCTCACCGTCTTTCATTGCCATACGGAATTCCGGATGAGCATTCATCAGGCGGGCAAGAA
TGTGAATAAAGGCCGGATAAAACTTGTGCTTATTTTTCTTTACGGTCTTTAAAAAGGCCGTAATATCCAGCTGAACGGT
CTGGTTATAGGTACATTGAGCAACTGACTGAAATGCCTCAAAATGTTCTTTACGATGCCATTGGGATATATCAACGGTG
GTATATCCAGTGATTTTTTTCTCCATTTTAGCTTCCTTAGCTCCTGAAAATCTCGATAACTCAAAAAATACGCCCGGTAG
TGATCTTATTTCATTATGGTGAAAGTTGGAACCTCTTACGTGCCGATCAACGTCTCATTTTCGCCAAAAGTTGGCCCAG
GGCTTCCCGGTATCAACAGGGACACCAGGATTTATTTATTCTGCGAAGTGATCTTCCGTCACAGGTATTTATTCGGCG
CAAAGTGCGTCGGGTGATGCTGCCAACTTACTGATTTAGTGTATGATGGTGTTTTTGAGGTGCTCCAGTGGCTTCTGT
TTCTATCAGCTGTCCCTCCTGTTCAGCTACTGACGGGGTGGTGCGTAACGGCAAAAGCACCGCCGGACATCAGCGC
TAGCGGAGTGTATACTGGCTTACTATGTTGGCACTGATGAGGGTGTCAGTGAAGTGCTTCATGTGGCAGGAGAAAAA
AGGCTGCACCGGTGCGTCAGCAGAATATGTGATACAGGATATATTCCGCTTCCTCGCTCACTGACTCGCTACGCTCG
GTCGTTCGACTGCGGCGAGCGGAAATGGCTTACGAACGGGGCGGAGATTTCCTGGAAGATGCCAGGAAGATACTTA
ACAGGGAAGTGAGAGGGCCGCGGCAAAGCCGTTTTTCCATAGGCTCCGCCCCCCTGACAAGCATCACGAAATCTGA
CGCTCAAATCAGTGGTGGCGAAACCCGACAGGACTATAAAGATACCAGGCGTTTCCCCCTGGCGGCTCCCTCGTGC
GCTCTCCTGTTCCTGCCTTTCGGTTTACCGGTGTCATTCCGCTGTTATGGCCGCGTTTGTCTCATTCCACGCCTGACA
CTCAGTTCCGGGTAGGCAGTTCGCTCCAAGCTGGACTGTATGCACGAACCCCCCGTTCAGTCCGACCGCTGCGCCT
TATCCGGTAACTATCGTCTTGAGTCCAACCCGGAAAGACATGCAAAAGCACCACTGGCAGCAGCCACTGGTAATTGAT
TTAGAGGAGTTAGTCTTGAAGTCATGCGCCGGTTAAGGCTAAACTGAAAGGACAAGTTTTGGTGACTGCGCTCCTCC
AAGCCAGTTACCTCGGTTCAAAGAGTTGGTAGCTCAGAGAACCTTCGAAAAACCGCCCTGCAAGGCGGTTTTTTCGT
TTTCAGAGCAAGAGATTACGCGCAGACCAAAACGATCTCAAGAAGATCATCTTATTAATCAGATAAAATATTTCTAGATT
TCAGTGCAATTTATCTCTTCAAATGTAGCACCTGAAGTCAGCCCCATACGATATAAGTTGTAATTCTCATGTTTGACAGC
TTATCGATGCAT

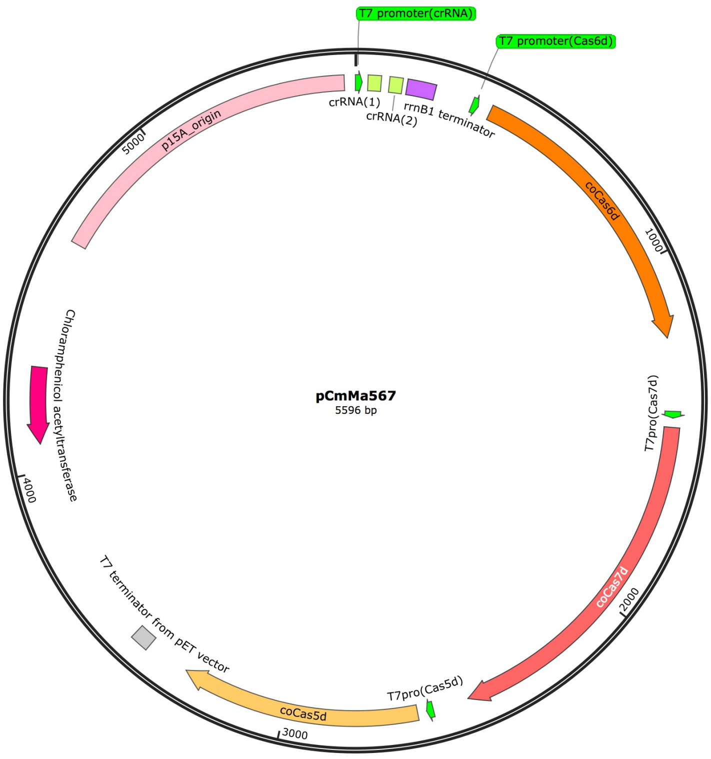

Supplementary Fig. 8. Plasmid vector pCmMa567.

1..19: T7 promoter
36..72: crRNA(1)
96..132: crRNA(2)
146..225: rrnB1 terminator
335..353: T7 promoter
390..1223: coCas6d
1430..1448T7 promoter
1475..2476: coCas7d
2579..2597: T7 promoter
2624..3298: coCas5d
3422..3474: T7 terminator from pET vector
4076..4291 (complement): Chloramphenicol acetyltransferase gene
4653..5565: p15A origin

>pCmMa567
TAATACGACTCACTATAGGCTAGTGCTAGCACCTCGTTCCAATTAATCTTAAGCCCTATTAGGGATTGAAACGGAGACC
CTCAATTGTCGGTCTCGTTCCAATTAATCTTAAGCCCTATTAGGGATTGAAACTTTTTTTGTCGACCCAGGCATCAAATA
AAACGAAAGGCTCAGTCGAAAGACTGGGCCTTTCGTTTTATCTGTTGTTTGTCGGTGAACGCTCTCGTTGATTTTCCG
AACTTTAATGCGGTAGTTTATCACAGTTAAATTGCTAACGCAGTCAGGCACCGTGTATCGATCTCGAGCTTCGATCCGA
TAGACTAGCCGCTGGTAATAATACGACTCACTATAGGGAGAGATTTCTATTAGTACTTCATAAGGAGGACAGAATGCCG
TATAGCCTGGTGCTGAACCTGACCCCGCGCAGCCCGATTTATCCGAACTTCCTGACCGGCCGCCATCTGCATGCGCT
GTTCCTGACCCTGGTGAGCAGCGTGGATCAGGAACTGGGCAACATTCTGCATACCGCGGAAGCGGATAAAGCGTTC
ACCCTGAGCCCGCTGCAGATGCAGAGCGGCGGCAAAACCATTAACAGCCCGCAGTGGCGCCATGAACGCGAAATT
GCGAGCGAAACCCCGTGCTGGTGGCGCATTAGCCTGCTGGATGATCGCCTGTTCGGCAAACTGACCAGCCTGTGG
CTGAACCTGAACCCGAAACAGCCGTGGCATCTGGGCAGCGCGGATCTGGTGATTACCAGCGTGCTGGCGACCCCG
CAGAGCGTGCAGCCGTGGGCGAACAGCTGCACCTATCAGTATCTGTATGAAAACGCGAGCGAAACCAACCGCGAGT
TCGATTTCCTGTTCGCGACCCCGGTGACCTTCCGCCAGGGCAAATTCGATAGCGCGCTGCCGACCCGCGAACTGGT
GTTCAACAGCCTGCTGGGCCGCTGGAACCGCTATAGCGGCATTCCGTTCGATAGCATTGCGCTGGAAAGCATTTTCC
CGAGCTTCTTCGATATTCAGACCAAACTGGCGGATGAAGCGTATAAAAACCAGAGCATTGGCTGCGTGGGCGAAATT
CATTATCGCCTGCTGGGCGAAGTGGAACCGGCGAAAATTAAAGCGATTAACGCGCTGGCGGATTTCGCGCTGTATGC
GGGCGTGGGCCGCAAAACCACCATGGGCATGGGCATGACCCGCCGCATTAGCAAAGATAAACGCTAATAGCTTTCCT
GTGAGCAGCGAAAGAATTCCTAGCATAACCCCTTGGGGCCTCTAAACGGGTCTTGAGGGGTTTTTTGTTATACGCGA
GATAATCACTTGCATAGCTGCGTATGGAGGAAGCAACTCTTGAGTGTTAATATGTTGACCCCTGTATTAGGGATGCGG

GTAGTAGATCGGTCTGGATCCGCGTACTTCATTATGTATATTAATACGACTCACTATAGGGAGAAGATCTATAAAGGAGG
TAAATAATGACCTTCCTGACCAGCGTGGATGCGAAATTCTTCCATAGCGAAATTCCGTATAAACCGATGGGCAAATATG
TGCATTTCCTGACCATTCGCGTGACCGAAAGCTATCCGCTGTTCCAGACCGATGGCGAACTGAACAAAGCGCGCGT
GCGCGCGGGCATTGACAGCAAGAAAACCATTAGCCGCCTGAGCATGTTCAAACGCAAACAGAGCACCCCGGAACGC
CTGGTGGGCCGCGAACTGCTGCGCAACTATGGCCTGATTACCGCGGAAGAATGCGAATATAACGTGAAATTCGCGAT
GAACAACGCGGATTGCATTATTTATGGCTTCGCGATTGGCGATAGCGGCAGCGAAAAAAGCAAAGTGGTGGTGGATA
CCGCGTTCAGCATTACCCCGTTCGATGAAAGCCATGAGAGCTTCACCCTGAACGCGCCGTATGAAAACGGCACGATG
GCGAGCAAAGGCGAAAACAACACCAAAGTGGGCGAAGTGACCAGCCGCATTAACCAGCAGGATCATATTCGCCCGC
AGGTGTTCTTCCCGAGCATTGTGACCCTGAAAGATCCGACCGAAGCGAGCTTCCTGTATGTGTTCAACAACATTCTG
CGCACCCGCCATTATGGCGCGCAGACCACCCGCACCGGCCGCGTGCGCAACGAACTGATTGGCGTGATTTTCGCG
GATGGCGAAATTGTGAGCAACCTGCGCTGGACCCAGGCGATTTATGATCGCCTGCCGGATGAAGTGCTGCATAGCAT
TGATCCGCTGGATGAAGATCTGGTGATGGAAAAAGCGACCGAAGCGATTCAGGCGCTGATGGCGGAAGAATTTATTG
TGCATACCGATTTCATTGGCGAAAACTTCCAGCCGCTGCTGACCGAAGTGAAAACCCTGACCGGCACCGAAGCGGG
CATTCTGAGCGTGCTGGATCAGGCGAACAAAGAAAGCAAAAAATATTTCGAACAGTATATTGAAAAGAAAAAGGCGGA
AAAGAAATAAACGCGTGCTGCGTGGTCAAATGTGCGTAGACCAACCCCTTGCGGCCTCAATCGGGGGGGATGGGGT
TTTTTGTCGAGCTCGTCCTGTGTTAGCTGGAGGGTATAATACGACTCACTATAGGGAGAAAATTCATAAAGGAGGTAAA
TAATGGTGCATATTTATAGCTGCCAGCTGGAACTGCATGATAGCCTGTATTATGCGACCCGCGAAATTGGCCGCCTGTA
TGAAAGCGAACCGGTGATTCATAACTATGCGCTGTGCTATGCGCTGGGCCTGGTGAACAGCGATAGCTATCGCTATTT
CTGCAGCGAACAGATTCCGCAGTATCAGGAACATCTGAACCCGCTGAACGAAGAAAAAATTTATGTGACCCCGGCGC
GCGCGATTGCGCATACCGCGGTGCTGAACACCTGGAAATATGCGAACAACAACTATCATGTGGAAATGGAAAAAACC
CAGAAAAACATTCCGAGCTTCGGCCGCGCGAAAGAAATTGCGCCGGAAAGCATTTTCGAATGCTTCATTATTAGCCAT
CATCCGCTGCAGCTGCCGAAATGGATTCGCCTGGGCAAATGGATGAGCAAAGCGGAAGTGAAACTGACCGAACTGA
GCCTGAGCAAACAGAAAGAAGATCTGTTCATTTATCCGTATCCGCTGAACCCGCTGGATGTGATGTTCACCCATCAGG
TGATTGGCTATGATGTGATTAACATGCCGCCGGTGAGCCTGATTCGCAACGTGCGCATGCGCGGCGAATATTATCAGA
TTAGCGATCGCCCGGATCTGAAAATTCCGGCACGTCTGAGCTATCATTTCGGCTAATAGCTTACCTGGAGATCAAGGA
GATTACTCTAACCCCATCGGCCGTCTTAGGGGTTTTTTGTCAAGCTTGGGAAAGGGTACCCAAAGGTGGTATACCAAT
GGTTACGTAAAGACGTTTGTACATAACAAAGCCCGAAAGGAAGCTGAGTTGGCTGCTGCCACCGCTGAGCAATAACT
AGCATAACCCCTTGGGGCCTCTAAACGGGTCTTGAGGGGTTTTTTGAAGCCGGCGGCAGGCCTCGATATCGAATTTC
TGCCATTCATCCGCTTATTATCACTTATTCAGGCGTAGCAACCAGGCGTTTAAGGGCACCAATAACTGCCTTAAAAAAA
TTACGCCCCGCCCTGCCACTCATCGCAGTACTGTTGTAATTCATTAAGCATTCTGCCGACATGGAAGCCATCACAAAC
GGCATGATGAACCTGAATCGCCAGCGGCATCAGCACCTTGTCGCCTTGCGTATAATATTTGCCCATGGTGAAAACGG
GGGCGAAGAAGTTGTCCATATTGGCCACGTTTAAATCAAAACTGGTGAAACTCACCCAGGGATTGGCTGAGACGAAA
AACATATTCTCAATAAACCCTTTAGGGAAATAGGCCAGGTTTTCACCGTAACACGCCACATCTTGCGAATATATGTGTAG
AAACTGCCGGAAATCGTCGTGGTATTCACTCCAGAGCGATGAAAACGTTTCAGTTTGCTCATGGAAAACGGTGTAACA
AGGGTGAACACTATCCCATATCACCAGCTCACCGTCTTTCATTGCCATACGGAATTCCGGATGAGCATTCATCAGGCG
GGCAAGAATGTGAATAAAGGCCGGATAAAACTTGTGCTTATTTTTCTTTACGGTCTTTAAAAAGGCCGTAATATCCAGC
TGAACGGTCTGGTTATAGGTACATTGAGCAACTGACTGAAATGCCTCAAAATGTTCTTTACGATGCCATTGGGATATAT
CAACGGTGGTATATCCAGTGATTTTTTTCTCCATTTTAGCTTCCTTAGCTCCTGAAAATCTCGATAACTCAAAAAATACG
CCCGGTAGTGATCTTATTTCATTATGGTGAAAGTTGGAACCTCTTACGTGCCGATCAACGTCTCATTTTCGCCAAAAGT
TGGCCCAGGGCTTCCCGGTATCAACAGGGACACCAGGATTTATTTATTCTGCGAAGTGATCTTCCGTCACAGGTATTT
ATTCGGCGCAAAGTGCGTCGGGTGATGCTGCCAACTTACTGATTTAGTGTATGATGGTGTTTTTGAGGTGCTCCAGTG
GCTTCTGTTTCTATCAGCTGTCCCTCCTGTTCAGCTACTGACGGGGTGGTGCGTAACGGCAAAAGCACCGCCGGAC
ATCAGCGCTAGCGGAGTGTATACTGGCTTACTATGTTGGCACTGATGAGGGTGTCAGTGAAGTGCTTCATGTGGCAG
GAGAAAAAAGGCTGCACCGGTGCGTCAGCAGAATATGTGATACAGGATATATTCCGCTTCCTCGCTCACTGACTCGCT
ACGCTCGGTCGTTCGACTGCGGCGAGCGGAAATGGCTTACGAACGGGGCGGAGATTTCCTGGAAGATGCCAGGAA
GATACTTAACAGGGAAGTGAGAGGGCCGCGGCAAAGCCGTTTTTCCATAGGCTCCGCCCCCCTGACAAGCATCACG
AAATCTGACGCTCAAATCAGTGGTGGCGAAACCCGACAGGACTATAAAGATACCAGGCGTTTCCCCCTGGCGGCTCC
CTCGTGCGCTCTCCTGTTCCTGCCTTTCGGTTTACCGGTGTCATTCCGCTGTTATGGCCGCGTTTGTCTCATTCCACG
CCTGACACTCAGTTCCGGGTAGGCAGTTCGCTCCAAGCTGGACTGTATGCACGAACCCCCCGTTCAGTCCGACCGC
TGCGCCTTATCCGGTAACTATCGTCTTGAGTCCAACCCGGAAAGACATGCAAAAGCACCACTGGCAGCAGCCACTGG
TAATTGATTTAGAGGAGTTAGTCTTGAAGTCATGCGCCGGTTAAGGCTAAACTGAAAGGACAAGTTTTGGTGACTGCG
CTCCTCCAAGCCAGTTACCTCGGTTCAAAGAGTTGGTAGCTCAGAGAACCTTCGAAAAACCGCCCTGCAAGGCGGT
TTTTTCGTTTTCAGAGCAAGAGATTACGCGCAGACCAAAACGATCTCAAGAAGATCATCTTATTAATCAGATAAAATATT
TCTAGATTTCAGTGCAATTTATCTCTTCAAATGTAGCACCTGAAGTCAGCCCCATACGATATAAGTTGTAATTCTCATGTT
TGACAGCTTATCGATGCAT

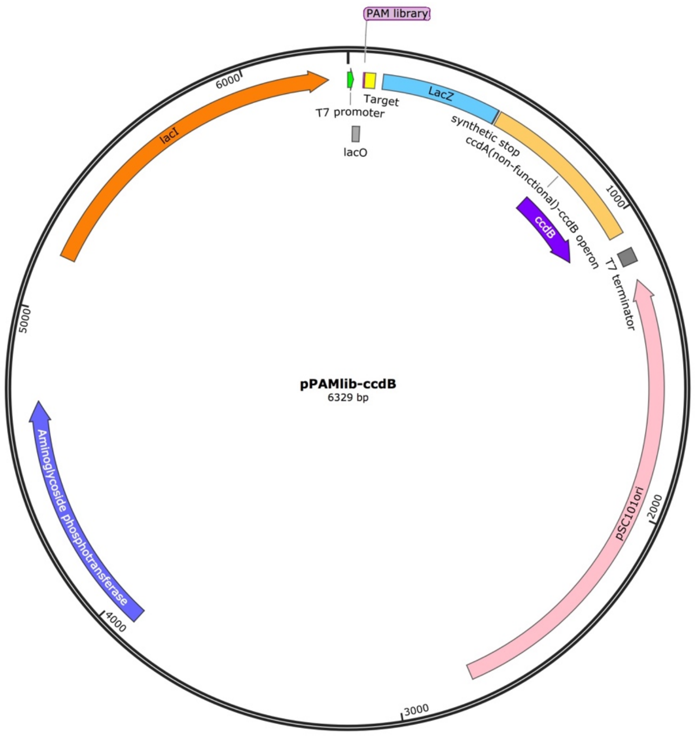

Supplementary Fig. 9. Plasmid vector pPAMlib-ccdB.

1..19: T7 promoter
19..46: lacO
56..90: Traget, 35nt
52..55: PAM library, 4nt
116..504: LacZ gene, #1 to #386 with synthetic stop codon
511..1063: ccdA(non-functional)-ccdB operon
758..1063: ccdB
1118..1164: T7 terminator
1221..2756(complement): pSC101ori
3912..4706: Aminoglycoside phosphotransferase
5185..6267: lacI

>pPAMlib-ccdB
TAATACGACTCACTATAGGGGAATTGTGAGCGGATAACAATTCCCCATCTANNNNCTGACAGCTAGCTCAGTCCTAGG
TATAATGCTAGCTTAACTTTAAGAAGGAGATATACATATGACCATGATTACGGATTCACTGGCCGTCGTTTTACAACGTC
GTGACTGGGAAAACCCTGGCGTTACCCAACTTAATCGCCTTGCAGCACATCCCCCTTTCGCCAGCTGGCGTAATAGC
GAAGAGGCACGCACCGATCGCCCTTCCCAACAGTTGCGCAGCTTAAATGGCGAATGGCGCTTTGCATGGTTCCCGG
CACAGAAACGGTTCCGGAAAGCTGGCTGGAGTGCGATCTTCCTGAGGCCGATACTGTCGTCGTCCCCTCAAACTGG
CAGATGCACGGTTACGATGCGCCCATCTACACCAACGTGACCTATCCCATTACGGTCAATCCGCCGTTTGTTCCCACG
GAGAATCCGACGGGTTGTTACTCGCTCACATTTAATTAGGGATCCGAAGTATGTCAAAAAGAGGTGTGCTATGAAGCA
GCGTATTACAGTGACAGTTGACAGCGACAGCTATCAGTTGCTCAAGGCATATATGATGTCAATATCTCCGGTCTGGTAA
GCACAACCATGCAGAATGAAGCCCGTCGTCTGCGTGCCGAACGCTGGAAAGCGGAAAATCAGGAAGGGATGGCTG
AGGTCGCCCGGTTTATTGAAATGAACGGCTCTTTTGCTGACGAGAACAGGGACTGGTGAAATGCAGTTTAAGGTTTA
CACCTATAAAAGAGAGAGCCGTTATCGTCTGTTTGTGGATGTACAGAGTGATATTATTGACACGCCCGGGCGACGGAT
GGTGATCCCCCTGGCCAGTGCACGTCTGCTGTCAGATAAAGTCTCCCGTGAACTTTACCCGGTGGTGCATATCGGG
GATGAAAGCTGGCGCATGATGACCACCGATATGGCCAGTGTGCCGGTCTCCGTTATCGGGGAAGAAGTGGCTGATC
TCAGCCACCGCGAAAATGACATCAAAAACGCCATTAACCTGATGTTCTGGGGAATATAATAGCAAGAGCTCGAAAGGA
AGCTGAGTTGGCTGCTGCCACCGCTGAGCAATAACTAGCATAACCCCTTGGGGCCTCTAAACGGGTCTTGAGGGGT
TTTTTGAAGCCGGCGGCAGGCCTCGAATTCTGAGTAGGACAAATCCGCCGCCCTAGACCTAGGGTACGGGTTTTGCT
GCCCGCAAACGGGCTGTTCTGGTGTTGCTAGTTTGTTATCAGAATCGCAGATCCGGCTTCAGCCGGTTTGCCGGCT
GAAAGCGCTATTTCTTCCAGAATTGCCATGATTTTTTCCCCACGGGAGGCGTCACTGGCTCCCGTGTTGTCGGCAGC
TTTGATTCGATAAGCAGCATCGCCTGTTTCAGGCTGTCTATGTGTGACTGTTGAGCTGTAACAAGTTGTCTCAGGTGT
TCAATTTCATGTTCTAGTTGCTTTGTTTTACTGGTTTCACCTGTTCTATTAGGTGTTACATGCTGTTCATCTGTTACATTG
TCGATCTGTTCATGGTGAACAGCTTTGAATGCACCAAAAACTCGTAAAAGCTCTGATGTATCTATCTTTTTTACACCGTT

TTCATCTGTGCATATGGACAGTTTTCCCTTTGATATGTAACGGTGAACAGTTGTTCTACTTTTGTTTGTTAGTCTTGATG
CTTCACTGATAGATACAAGAGCCATAAGAACCTCAGATCCTTCCGTATTTAGCCAGTATGTTCTCTAGTGTGGTTCGTT
GTTTTTGCGTGAGCCATGAGAACGAACCATTGAGATCATGCTTACTTTGCATGTCACTCAAAAATTTTGCCTCAAAACT
GGTGAGCTGAATTTTTGCAGTTAAAGCATCGTGTAGTGTTTTTCTTAGTCCGTTATGTAGGTAGGAATCTGATGTAATG
GTTGTTGGTATTTTGTCACCATTCATTTTTATCTGGTTGTTCTCAAGTTCGGTTACGAGATCCATTTGTCTATCTAGTTCA
ACTTGGAAAATCAACGTATCAGTCGGGCGGCCTCGCTTATCAACCACCAATTTCATATTGCTGTAAGTGTTTAAATCTT
TACTTATTGGTTTCAAAACCCATTGGTTAAGCCTTTTAAACTCATGGTAGTTATTTTCAAGCATTAACATGAACTTAAATT
CATCAAGGCTAATCTCTATATTTGCCTTGTGAGTTTTCTTTTGTGTTAGTTCTTTTAATAACCACTCATAAATCCTCATAG
AGTATTTGTTTTCAAAAGACTTAACATGTTCCAGATTATATTTTATGAATTTTTTTAACTGGAAAAGATAAGGCAATATCTC
TTCACTAAAAACTAATTCTAATTTTTCGCTTGAGAACTTGGCATAGTTTGTCCACTGGAAAATCTCAAAGCCTTTAACCA
AAGGATTCCTGATTTCCACAGTTCTCGTCATCAGCTCTCTGGTTGCTTTAGCTAATACACCATAAGCATTTTCCCTACT
GATGTTCATCATCTGAGCGTATTGGTTATAAGTGAACGATACCGTCCGTTCTTTCCTTGTAGGGTTTTCAATCGTGGGG
TTGAGTAGTGCCACACAGCATAAAATTAGCTTGGTTTCATGCTCCGTTAAGTCATAGCGACTAATCGCTAGTTCATTTG
CTTTGAAAACAACTAATTCAGACATACATCTCAATTGGTCTAGGTGATTTTAATCACTATACCAATTGAGATGGGCTAGT
CAATGATAATTACTAGTCCTTTTCCTTTGAGTTGTGGGTATCTGTAAATTCTGCTAGACCTTTGCTGGAAAACTTGTAAA
TTCTGCTAGACCCTCTGTAAATTCCGCTAGACCTTTGTGTGTTTTTTTTGTTTATATTCAAGTGGTTATAATTTATAGAATA
AAGAAAGAATAAAAAAAGATAAAAAGAATAGATCCCAGCCCTGTGTATAACTCACTACTTTAGTCAGTTCCGCAGTATTA
CAAAAGGATGTCGCAAACGCTGTTTGCTCCTCTACAAAACAGACCTTAAAACCCTAAAGGCTTAAGTAGCACCCTCGC
AAGCTCGGGCAAATCGCTGAATATTCCTTTTGTCTCCGACCATCAGGCACCTGAGTCGCTGTCTTTTTCGTGACATTC
AGTTCGCTGCGCTCACGGCTCTGGCAGTGAATGGGGGTAAATGGCACTACAGGCGCCTTTTATGGATTCATGCAAGG
AAACTACCCATAATACAAGAAAAGCCCGTCACGGGCTTCTCAGGGCGTTTTATGGCGGGTCTGCTATGTGGTGCTATC
TGACTTTTTGCTGTTCTGCAGTTCCTGCCCTCTGATTTTCCAGTCTGACCACTTCGGATTATCCCGTGACAGGTCATT
CAGACTGGCTAATGCACCCAGTAAGGCAGCGGTATCATCAACAGGCTTACCCGTCTTACTGTCAATTCTTGAAGACGA
AAGGGCCTCGTGATACGCCTATTTTTATAGGTTAATGTCATGATAATAATGGTTTCTTAGACGTCAGGTGGCACTTTTCG
GGGAAATGTGCGCGGAACCCCTATTTGTTTATTTTTCTAAATACATTCAAATATGTATCCGCTCATGAGACAATAACCCT
GATAAATGCTTCAATAATATTGAAAAAGGAAGAGTATGAGTATTCAACATTTCCGTGTCGCCCTTATTCCCTTTTTTGCG
GCATTTTGCCTTCCTGTTTTTGCTCACCCAGAAACGCTGGTGAAAGTAAAAGATGCTGAAGATCAGTTGGGTGCACG
AGTGGGTTACATCGAACTGGATCTCAACAGCGGTAAGATCCTTGAGAGTTTTCGCCCCGAAGAACGCTCATGTTTGA
CAGCTTATCATCGATATGCTTTAATGCGGTAGTGATCAAGAGACAGGATGAGGATCGTTTCGCATGATTGAACAAGATG
GATTGCACGCAGGTTCTCCGGCCGCTTGGGTGGAGAGGCTATTCGGCTATGACTGGGCACAACAGACAATCGGCTG
CTCTGATGCCGCCGTGTTCCGGCTGTCAGCGCAGGGGCGCCCGGTTCTTTTTGTCAAGACCGACCTGTCCGGTGC
CCTGAATGAACTGCAGGACGAGGCAGCGCGGCTATCGTGGCTGGCCACGACGGGCGTTCCTTGCGCAGCTGTGCT
CGACGTTGGCACTGAAGCGGGAAGGGACTGGCTGCTATTGGGCGAAGTGCCGGGGCAGGATCTCCTGTCATCTCA
CCTTGCTCCTGCCGAGAAAGTATCCATCATGGCTGATGCAATGCGGCGGCTGCATACGCTTGATCCGGCTACCTGCC
CATTCGACCACCAAGCGAAACATCGCATCGAGCGAGCACGTACTCGGATGGAAGCCGGTCTTGTCGATCAGGATGAT
CTGGACGAAGAGCATCAGGGGCTCGCGCCAGCCGAACTGTTCGCCAGGCTCAAGGCGCGCATGCCCGACGGCGA
GGATCTCGTCGTGACCCATGGCGATGCCTGCTTGCCGAATATCATGGTGGAAAATGGCCGCTTTTCTGGATTCATCG
ACTGTGGCCGGCTGGGTGTGGCGGACCGCTATCAGGACATAGCGTTGGCTACCCGTGATATTGCTGAAGAGCTTGG
CGGCGAATGGGCTGACCGCTTCCTCGTGCTTTACGGTATCGCCGCTCCCGATTCGCAGCGCATCGCCTTCTATCGC
CTTCTTGACGAGTTCTTCTGAGCGGGACTCTGGGGTTCGGCACACAGCCCAGCTTGGAGCGAACGACCTACACCGA
ACTGAGATACCTACAGCGTGAGCTATGAGAAAGATATCACGCGTACCGCTAGCCAGGAAGAGTTTGTAGAAACGCAA
AAAGGCCATCCGTCAGGATGGCCTTCTGCTTAGTTTGATGCCTGGCAGTTTATGGCGGGCGTCCTGCCCGCCACCC
TCCGGGCCGTTGCTTCACAACGTTCAAATCCGCTCCCGGCGGATTTGTCCTACTCAGGAGAGCGTTCACCGACAAA
CAACAGATAAAACGAAAGGCCCAGTCTTCCGACTGAGCCTTTCGTTTTATTTGATGCCTGGCAGTTCCCTACTCTCGC
GTTAACGCTAGCATGGATAAGCTTGCTTGGACTCCTGTTGATAGATCCACCGGTGCAAAACCTTTCGCGGTATGGCAT
GATAGCGCCCGGAAGAGAGTCAATTCAGGGTGGTGAATGTGAAACCAGTAACGTTATACGATGTCGCAGAGTATGCC
GGTGTCTCTTATCAGACCGTTTCCCGCGTGGTGAACCAGGCCAGCCACGTTTCTGCGAAAACGCGGGAAAAAGTGG
AAGCGGCGATGGCGGAGCTGAATTACATTCCCAACCGCGTGGCACAACAACTGGCGGGCAAACAGTCGTTGCTGAT
TGGCGTTGCCACCTCCAGTCTGGCCCTGCACGCGCCGTCGCAAATTGTCGCGGCGATTAAATCTCGCGCCGATCAA
CTGGGTGCCAGCGTGGTGGTGTCGATGGTAGAACGAAGCGGCGTCGAAGCCTGTAAAGCGGCGGTGCACAATCTT
CTCGCGCAACGCGTCAGTGGGCTGATCATTAACTATCCGCTGGATGACCAGGATGCCATTGCTGTGGAAGCTGCCTG
CACTAATGTTCCGGCGTTATTTCTTGATGTCTCTGACCAGACACCCATCAACAGTATTATTTTCTCCCATGAAGACGGT
ACGCGACTGGGCGTGGAGCATCTGGTCGCATTGGGTCACCAGCAAATCGCGCTGTTAGCGGGCCCATTAAGTTCTG
TCTCGGCGCGTCTGCGTCTGGCTGGCTGGCATAAATATCTCACTCGCAATCAAATTCAGCCGATAGCGGAACGGGAA
GGCGACTGGAGTGCCATGTCCGGTTTTCAACAAACCATGCAAATGCTGAATGAGGGCATCGTTCCCACTGCGATGCT
GGTTGCCAACGATCAGATGGCGCTGGGCGCAATGCGCGCCATTACCGAGTCCGGGCTGCGCGTTGGTGCGGACAT
CTCGGTAGTGGGATACGACGATACCGAAGACAGCTCATGTTATATCCCGCCGTTAACCACCATCAAACAGGATTTTCG
CCTGCTGGGGCAAACCAGCGTGGACCGCTTGCTGCAACTCTCTCAGGGCCAGGCGGTGAAGGGCAATCAGCTGTT
GCCCGTCTCACTGGTGAAAAGAAAAACCACCCTGGCGCCCAATACGCAAACCGCCTCTCCCCGCGCGTTGGCCGAT

TCATTAATGCAGCTGGCACGACAGGTTTCCCGACTGGAAAGCGGGCAGTGAGCGCAACGCTTAATTAATGTGAGTTA
GCTCACTCATTAGGCACCCTCTCGATCCCGCGAAAT

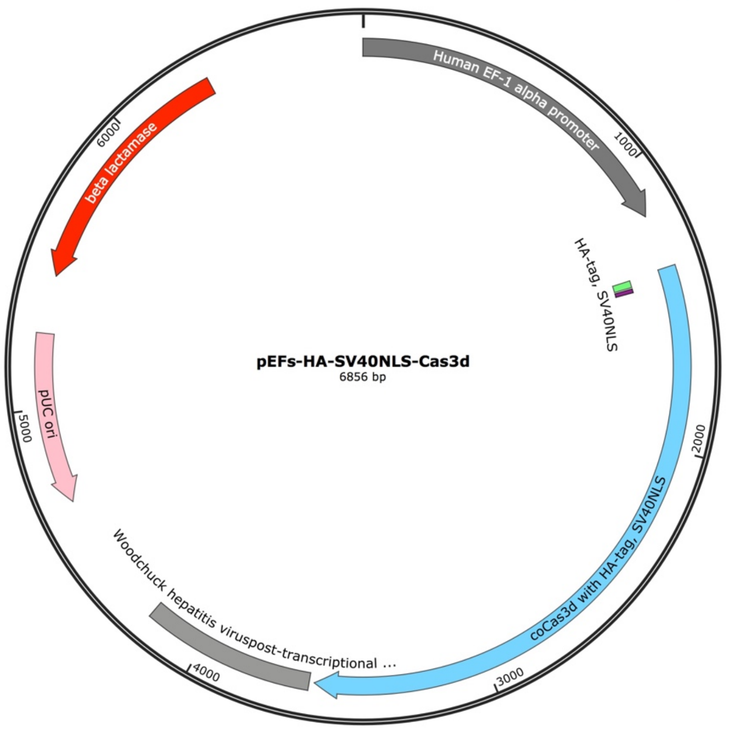

Supplementary Fig. 10. Plasmid vector pEFs-HA-SV40NLS-Cas3d.

1..1182: Human EF-1 alpha promoter
1370..3595: coCas3d with HA-tag, SV40NLS
1376..1402: HA-tag
1409..1423: SV40NLS
3613..4201: Woodchuck hepatitis virus post-transcriptionalregulatory element (WPRE)
5456..6316(complement): beta lactamase
4668..5256(complement): pUC ori

>pEFs-HA-SV40NLS-Cas3d
GCTCCGGTGCCCGTCAGTGGGCAGAGCGCACATCGCCCACAGTCCCCGAGAAGTTGGGGGGAGGGGTCGGCAAT
TGAACCGGTGCCTAGAGAAGGTGGCGCGGGGTAAACTGGGAAAGTGATGTCGTGTACTGGCTCCGCCTTTTTCCCG
AGGGTGGGGGAGAACCGTATATAAGTGCAGTAGTCGCCGTGAACGTTCTTTTTCGCAACGGGTTTGCCGCCAGAAC
ACAGGTAAGTGCCGTGTGTGGTTCCCGCGGGCCTGGCCTCTTTACGGGTTATGGCCCTTGCGTGCCTTGAATTACTT
CCACGCCCCTGGCTGCAGTACGTGATTCTTGATCCCGAGCTTCGGGTTGGAAGTGGGTGGGAGAGTTCGAGGCCTT
GCGCTTAAGGAGCCCCTTCGCCTCGTGCTTGAGTTGAGGCCTGGCCTGGGCGCTGGGGCCGCCGCGTGCGAATCT
GGTGGCACCTTCGCGCCTGTCTCGCTGCTTTCGATAAGTCTCTAGCCATTTAAAATTTTTGATGACCTGCTGCGACGC
TTTTTTTCTGGCAAGATAGTCTTGTAAATGCGGGCCAAGATCTGCACACTGGTATTTCGGTTTTTGGGGCCGCGGGC
GGCGACGGGGCCCGTGCGTCCCAGCGCACATGTTCGGCGAGGCGGGGCCTGCGAGCGCGGCCACCGAGAATCG
GACGGGGGTAGTCTCAAGCTGGCCGGCCTGCTCTGGTGCCTGGCCTCGCGCCGCCGTGTATCGCCCCGCCCTGG
GCGGCAAGGCTGGCCCGGTCGGCACCAGTTGCGTGAGCGGAAAGATGGCCGCTTCCCGGCCCTGCTGCAGGGAG
CTCAAAATGGAGGACGCGGCGCTCGGGAGAGCGGGCGGGTGAGTCACCCACACAAAGGAAAAGGGCCTTTCCGT
CCTCAGCCGTCGCTTCATGTGACTCCACGGAGTACCGGGCGCCGTCCAGGCACCTCGATTAGTTCTCGAGCTTTTG
GAGTACGTCGTCTTTAGGTTGGGGGGAGGGGTTTTATGCGATGGAGTTTCCCCACACTGAGTGGGTGGAGACTGAA
GTTAGGCCAGCTTGGCACTTGATGTAATTCTCCTTGGAATTTGCCCTTTTTGAGTTTGGATCTTGGTTCATTCTCAAGC
CTCAGACAGTGGTTCAAAGTTTTTTTCTTCCATTTCAGGTGTCGTGAATTGCGGAAGGCCGTCAAGGCCACGTGTCT
TGTCCAGAGCTCAGGTGTCGTGAATTGCGTAGCATTTAGGTGACACTATAGAACTCACCTATCTCCCCAACACCTAATA
ACATTCAATCACTCTTTCCACTAACCACCTATCTACATCACCACCCACCACCACCAGCCCGGGATATCAGCTGACACC
ATGGCGTACCCATACGATGTTCCAGATTACGCTGACCCTAAGAAGAAGCGCAAGGTTTCTGGAGGCAACTACCAAGT
CACCCTGAAGCCTGTCTACTCCTGCCCTGCCGATGAAATCCCTGATGGCATCAAGGTCCCTCAAGGCTGGCGCCTG
TCCTGGCATCAAGTCGAAACCTGGAAGGCCCTGAACGATCCTGATATCGATGTCATCTTCAACACCGCCATGACCGG
CGATGGCAAGTCCCTGGCCGCCTACCTGCGCACCCTGCAAGGCTACTTCCCTATCATGGGCCTGTACCCTACCAAC
GAACTGGCCCGCGATCAACGCGGCCAAATCGAAGCCTACATCCAACGCTTCCAACCTACCGATCAACCTCGCGTCAA
CCTGCTGACCGGCCCTGAACTGGAACTGTACGCCGAACGCGATGGCAAGACCAAGGCCATCGCCCTGGAAACCCG
CTCCAAGCAATCCGAAATCCTGCTGACCAACCCTGATATCTTCCATTACCTGCATCGCGCCGCCTACCTGACCCCTTA

CGATAACCCTGATCAACTGTGGAACCGCATCGATAAGCATTTCGATCTGTTCCTGTTCGATGAGTTCCATGTCTTCGG
CACCCCTCAAGTCGCCTCCATCATCAACACCATGCTGCTGATCCGCCGCGCCAACCGCGGCAAGCGCTACCTGTTC
CTGTCCGCCACCCCTGATGAAGGCCTGCTGAAGCGCCTGGATAAGGCCGGCTTCCGCTACCGCTCCATCGATCCTG
TCCGCGAAGGCAAGTACCGCTTCCCTGATACCCCTGAAGAAGCCAACTCCCTGGCCCAACAAGGCTGGCGCCAAGT
CACCTCCGAAATCGAACTGTCCTTCATCCCTCTGCCTTCCTCCTTCCAAACCTCCGAAAACTGGCTGAAGGAAAACA
AGGAACGCATCCTGGATTACTTCAAGCGCTACCCTGGCTCCAAGGGCGCCATCATCCTGAACTCCATCGCCTCCGTC
AAGCGCCTGCTGCCTATCTTCCGCGAACTGCTGGCCACCATCGGCCTGACCGTCGGCGAAAACACCGGCCTGTCC
GGCACCCGCGAAAAGCTGGCCTCCCTGAACCGCGATCTGGTCATCGGCACCTCCACCATCGATGTCGGCGTCGATT
TCAAGATCAACTTCCTGATCTTCGAATCCTCCGATGCCGGCAACTTCATCCAACGCTTCGGCCGCCTGGGCCGCCAT
TCCGGCTACGATCGCAAGGGCACCGCCGTCAAGTTCACCAACTTCACCGCCATCGCCCTGGTCCCTAAGTTCTTCCT
GGAACGCCTGTTCGAAAAGAAGGATGCCCCTCTGCAAGTCGGCGAACGCTACGATCGCATCCAACTGCAAGAAGCC
ATCAAGTCCAACTACCGCCATATCAACAACTTCGAAGGCTACTACCAACGCTGGGGCGCCGTCCAATCCTTCCAACT
GTGGTGGAACCTGGGCTCCCCTAAGATCAAGTCCCAATACGGCGAATCCCGCCAAAAGTTCCAACAAGAATGCGAA
GAAGTCTTCGATACCTCCCTGAAGCGCGTCGCCGGCCGCGTCAAGGGCTGGGCCGATGAATGGAAGGAACTGTCC
GGCAAGAACGGCAACCCTATCTTCGAAGATGCCTCCTCCTTCCGCGGCTCCTCCCCTCTGCTGTGCGGCCTGTACG
ATTCCACCGAACCTGAAGAATGCGATCGCTTCAAGACCTACGATCTGCCTTCCATCCTGGGCAACCTGGAAGTCGAA
GTCTGGCGCAAGGGCGAGTTCAAGCGCCAAATCGAAGCCACCAAGACCCCTATCGCCCGCCGCCGCTTCGATTACT
GCCTGGCCTTCCTGAACCTGAAGGGCTACCGCGAAGAACGCCTGAACTGGCGCTTCACCTACGATGGCGATCTGGG
CGAAATCGCCTCCGCCTGGAAGGTCCAAGTCCTGACCGGCATCGGCGTCTGGCAACCTGATAACCCTTGGCTGGAT
CGCATCTCCCGCGAACTGCGCGATCTGGCCCTGGTGTCCTTCGTCTTCGCCTACCCTGTCGCCGCCGTCCGCCAAC
GCCTGCAACTGCCTATGCATTTCGGCATCTACCCTATCTCCGATGAATCCTCCCTGCATTCCCCTCTGTCCCCTTACTC
CATCGCCATCGGCCAAGCCGCCCTGCTGCTGGATACCCTGGCCCATCGCTTCAAGGGCAAGGGCGGCGAAGTCTG
GATCTGCTGATCTAGAAATTCATCGACAATCAACCTCTGGATTACAAAATTTGTGAAAGATTGACTGGTATTCTTAACTA
TGTTGCTCCTTTTACGCTATGTGGATACGCTGCTTTAATGCCTTTGTATCATGCTATTGCTTCCCGTATGGCTTTCATTTT
CTCCTCCTTGTATAAATCCTGGTTGCTGTCTCTTTATGAGGAGTTGTGGCCCGTTGTCAGGCAACGTGGCGTGGTGT
GCACTGTGTTTGCTGACGCAACCCCCACTGGTTGGGGCATTGCCACCACCTGTCAGCTCCTTTCCGGGACTTTCGC
TTTCCCCCTCCCTATTGCCACGGCGGAACTCATCGCCGCCTGCCTTGCCCGCTGCTGGACAGGGGCTCGGCTGTTG
GGCACTGACAATTCCGTGGTGTTGTCGGGGAAGCTGACGTCCTTTCCATGGCTGCTCGCCTGTGTTGCCACCTGGA
TTCTGCGCGGGACGTCCTTCTGCTACGTCCCTTCGGCCCTCAATCCAGCGGACCTTCCTTCCCGCGGCCTGCTGCC
GGCTCTGCGGCCTCTTCCGCGTCTTCGCCTTCGCCCTCAGACGAGTCGGATCTCCCTTTGGGCCGCCTCCCCGCC
CAGGTTCCTTGAGCATCTGGAATTCTGCCTAATAAAAAACATTTATTTTCATTGCAATGATGTATTTAAATTATTTCTGAAT
ATTTTACTAAAAAGGGAATGTGGGAGGTCAGTGCATTTAAGACATAAAGAAATGAAGAGCTAGTTCAAACCTTGGGAA
AATACACTATATCTTAAACTCCATGAAAGAAGGTGAGGCTGCAAACAGCTAATGCACATTGGCAACAGCCCCTGATGC
CTCTGCCTTATTCATCCCTCAGAAAAGGATTCAAGGCATAGTGTTACCATCAACCACCTTAACTTCATTTTTCTTATTCA
ATACCTAGGTAGGTAGATGCTAGATTCTGGAAATAAAATATGAGTCTCAAGTGGTCCTTGTCCTCTCTCCCAGTCAAAT
TCTGAATCTAGTTGGCAAGATTCTGAAATCAAGGCATATAATCAGTAATAAGTGATGATAGAAGGGTATTTCCATAGGCT
CCGCCCCCCTGACGAGCATCACAAAAATCGACGCTCAAGTCAGAGGTGGCGAAACCCGACAGGACTATAAAGATAC
CAGGCGTTTCCCCCTGGAAGCTCCCTCGTGCGCTCTCCTGTTCCGACCCTGCCGCTTACCGGATACCTGTCCGCCT
TTCTCCCTTCGGGAAGCGTGGCGCTTTCTCATAGCTCACGCTGTAGGTATCTCAGTTCGGTGTAGGTCGTTCGCTCC
AAGCTGGGCTGTGTGCACGAACCCCCCGTTCAGCCCGACCGCTGCGCCTTATCCGGTAACTATCGTCTTGAGTCCA
ACCCGGTAAGACACGACTTATCGCCACTGGCAGCAGCCACTGGTAACAGGATTAGCAGAGCGAGGTATGTAGGCGG
TGCTACAGAGTTCTTGAAGTGGTGGCCTAACTACGGCTACACTAGAAGAACAGTATTTGGTATCTGCGCTCTGCTGAA
GCCAGTTACCTTCGGAAAAAGAGTTGGTAGCTCTTGATCCGGCAAACAAACCACCGCTGGTAGCGGTGGTTTTTTTG
TTTGCAAGCAGCAGATTACGCGCAGAAAAAAAGGATCTCAAGAAGATCCTTTGATCTTTTCTACGGGGTCTGACGCTC
AGTGGAACGAAAACTCACGTTAAGGGATTTTGGTCATGAGATTATCAAAAAGGATCTTCACCTAGATCCTTTTAAATTAA
AAATGAAGTTTTAAATCAATCTAAAGTATATATGAGTAAACTTGGTCTGACAGCGGCCGCAAATGCTAAACCACTGCAG
TGGTTACCAGTGCTTGATCAGTGAGGCACCGATCTCAGCGATCTGCCTATTTCGTTCGTCCATAGTGGCCTGACTCC
CCGTCGTGTAGATCACTACGATTCGTGAGGGCTTACCATCAGGCCCCAGCGCAGCAATGATGCCGCGAGAGCCGCG
TTCACCGGCCCCCGATTTGTCAGCAATGAACCAGCCAGCAGGGAGGGCCGAGCGAAGAAGTGGTCCTGCTACTTTG
TCCGCCTCCATCCAGTCTATGAGCTGCTGTCGTGATGCTAGAGTAAGAAGTTCGCCAGTGAGTAGTTTCCGAAGAGT
TGTGGCCATTGCTACTGGCATCGTGGTATCACGCTCGTCGTTCGGTATGGCTTCGTTCAACTCTGGTTCCCAGCGGT
CAAGCCGGGTCACATGATCACCCATATTATGAAGAAATGCAGTCAGCTCCTTAGGGCCTCCGATCGTTGTCAGAAGTA
AGTTGGCCGCGGTGTTGTCGCTCATGGTAATGGCAGCACTACACAATTCTCTTACCGTCATGCCATCCGTAAGATGCT
TTTCCGTGACCGGCGAGTACTCAACCAAGTCGTTTTGTGAGTAGTGTATACGGCGACCAAGCTGCTCTTGCCCGGCG
TCTATACGGGACAACACCGCGCCACATAGCAGTACTTTGAAAGTGCTCATCATCGGGAATCGTTCTTCGGGGCGGAA
AGACTCAAGGATCTTGCCGCTATTGAGATCCAGTTCGATATAGCCCACTCTTGCACCCAGTTGATCTTCAGCATCTTTT
ACTTTCACCAGCGTTTCGGGGTGTGCAAAAACAGGCAAGCAAAATGCCGCAAAGAAGGGAATGAGTGCGACACGAA
AATGTTGGATGCTCATACTCGTCCTTTTTCAATATTATTGAAGCATTTATCAGGGTTACTAGTACGTCTCTCAAGGATAA
GTAAGTAATATTAAGGTACGGGAGGTATTGGACAAGAGAAATGTTCTGGCACCTGCACTTGCACTGGGGACAGCCTAT
TTTGCTAGTTTGTTTTGTTTCGTTTTGTTTTGATGGAGAGCGTATGTTAGTACTATCGATTCACACAAAAAACCAACACA

CAGATGTAATGAAAATAAAGATATTTTATTGCGGCTGCATCTCAATTAGTCAGCAACCATAGTCCCGCCCCTAACTCCG
CCCATCCCGCCCCTAACTCCGCCCAGTTCCGCCCATTCTCCGCCCCATGGCTGACTAATTTTTTTTATTTATGCAGAG
GCCGAGGCCGCCTCGGCCTCTGAGCTATTCCAGAAGTAGTGAGGAGGCTTTTTTGGAGGCCTAGGCTTTTGCAAAA
AGCTTTGCAAAGATGGATAAAGTTTTAAACAGAGAGGAATCTTTGCAGCTAATGGACCTTCTAGGTCTTGAAAGGAGT
GCGTGAG

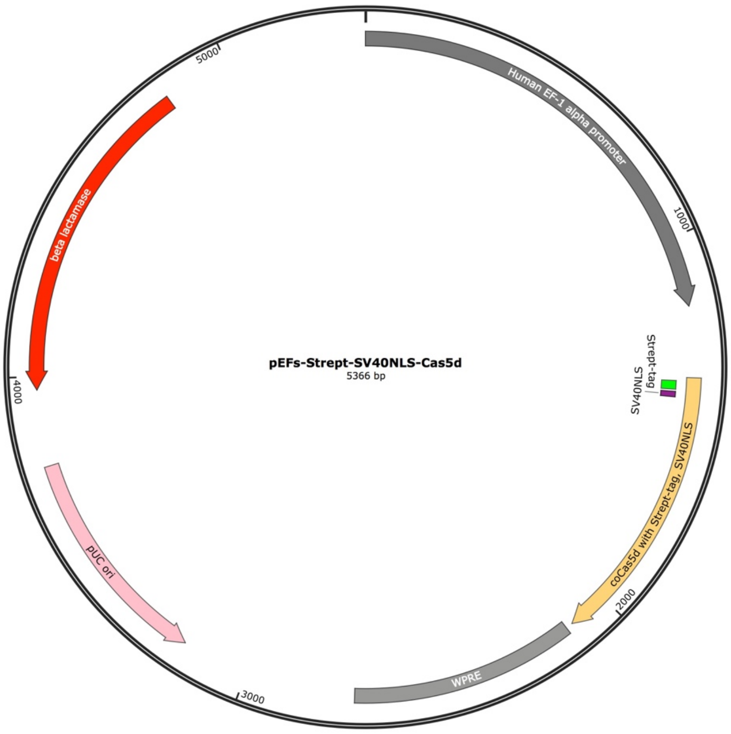

Supplementary Fig. 11. Plasmid vector pEFs-Strept-SV40NLS-Cas5d.

1..1182: Human EF-1 alpha promoter
1370..2104: coCas5d with Strept-tag, SV40NLS
1379..1402: Strept-tag
1409..1423: SV40NLS
2123..2711: Woodchuck hepatitis virus posttranscriptional regulatory element (WPRE)
3178..3766(complement): pUC ori
3966..4826(complement): beta lactamase

>pEFs-Strept-SV40NLS-Cas5d
GCTCCGGTGCCCGTCAGTGGGCAGAGCGCACATCGCCCACAGTCCCCGAGAAGTTGGGGGGAGGGGTCGGCAAT
TGAACCGGTGCCTAGAGAAGGTGGCGCGGGGTAAACTGGGAAAGTGATGTCGTGTACTGGCTCCGCCTTTTTCCCG
AGGGTGGGGGAGAACCGTATATAAGTGCAGTAGTCGCCGTGAACGTTCTTTTTCGCAACGGGTTTGCCGCCAGAAC
ACAGGTAAGTGCCGTGTGTGGTTCCCGCGGGCCTGGCCTCTTTACGGGTTATGGCCCTTGCGTGCCTTGAATTACTT
CCACGCCCCTGGCTGCAGTACGTGATTCTTGATCCCGAGCTTCGGGTTGGAAGTGGGTGGGAGAGTTCGAGGCCTT
GCGCTTAAGGAGCCCCTTCGCCTCGTGCTTGAGTTGAGGCCTGGCCTGGGCGCTGGGGCCGCCGCGTGCGAATCT
GGTGGCACCTTCGCGCCTGTCTCGCTGCTTTCGATAAGTCTCTAGCCATTTAAAATTTTTGATGACCTGCTGCGACGC
TTTTTTTCTGGCAAGATAGTCTTGTAAATGCGGGCCAAGATCTGCACACTGGTATTTCGGTTTTTGGGGCCGCGGGC
GGCGACGGGGCCCGTGCGTCCCAGCGCACATGTTCGGCGAGGCGGGGCCTGCGAGCGCGGCCACCGAGAATCG
GACGGGGGTAGTCTCAAGCTGGCCGGCCTGCTCTGGTGCCTGGCCTCGCGCCGCCGTGTATCGCCCCGCCCTGG
GCGGCAAGGCTGGCCCGGTCGGCACCAGTTGCGTGAGCGGAAAGATGGCCGCTTCCCGGCCCTGCTGCAGGGAG
CTCAAAATGGAGGACGCGGCGCTCGGGAGAGCGGGCGGGTGAGTCACCCACACAAAGGAAAAGGGCCTTTCCGT
CCTCAGCCGTCGCTTCATGTGACTCCACGGAGTACCGGGCGCCGTCCAGGCACCTCGATTAGTTCTCGAGCTTTTG
GAGTACGTCGTCTTTAGGTTGGGGGGAGGGGTTTTATGCGATGGAGTTTCCCCACACTGAGTGGGTGGAGACTGAA
GTTAGGCCAGCTTGGCACTTGATGTAATTCTCCTTGGAATTTGCCCTTTTTGAGTTTGGATCTTGGTTCATTCTCAAGC
CTCAGACAGTGGTTCAAAGTTTTTTTCTTCCATTTCAGGTGTCGTGAATTGCGGAAGGCCGTCAAGGCCACGTGTCT
TGTCCAGAGCTCAGGTGTCGTGAATTGCGTAGCATTTAGGTGACACTATAGAACTCACCTATCTCCCCAACACCTAATA
ACATTCAATCACTCTTTCCACTAACCACCTATCTACATCACCACCCACCACCACCAGCCCGGGATATCAGCTGCCACC
ATGGCTAGGTGGAGCCACCCGCAGTTCGAAAAGGACCCTAAGAAAAAGCGGAAGGTGAGCGGAGTCCATATCTACT
CCTGCCAACTGGAACTGCATGATTCCCTGTACTACGCCACCCGCGAAATCGGCCGCCTGTACGAATCCGAACCTGTC
ATCCATAACTACGCCCTGTGCTACGCCCTGGGCCTGGTCAACTCCGATTCCTACCGCTACTTCTGCTCCGAACAAATC
CCTCAATACCAAGAACATCTGAACCCTCTGAACGAAGAAAAGATCTACGTCACCCCTGCCCGCGCCATCGCCCATAC
CGCCGTCCTGAACACCTGGAAGTACGCCAACAACAACTACCATGTCGAAATGGAAAAGACCCAAAAGAACATCCCTT
CCTTCGGCCGCGCCAAGGAAATCGCCCCTGAATCCATCTTCGAATGCTTCATCATCTCCCATCATCCTCTGCAACTGC
CTAAGTGGATTCGCCTGGGCAAGTGGATGTCCAAGGCCGAAGTCAAGCTGACCGAACTGTCCCTGTCCAAGCAAAA

GGAAGATCTGTTCATCTACCCTTACCCTCTGAACCCTCTGGATGTCATGTTCACCCATCAAGTCATCGGCTACGATGT
CATCAACATGCCTCCTGTCTCCCTGATCCGCAACGTCCGCATGCGCGGCGAATACTACCAAATCTCCGATCGCCCTG
ATCTGAAGATCCCTGCCCGCCTGTCCTACCATTTCGGCTGATTCTAGAAATTCATCGACAATCAACCTCTGGATTACAA
AATTTGTGAAAGATTGACTGGTATTCTTAACTATGTTGCTCCTTTTACGCTATGTGGATACGCTGCTTTAATGCCTTTGTA
TCATGCTATTGCTTCCCGTATGGCTTTCATTTTCTCCTCCTTGTATAAATCCTGGTTGCTGTCTCTTTATGAGGAGTTGT
GGCCCGTTGTCAGGCAACGTGGCGTGGTGTGCACTGTGTTTGCTGACGCAACCCCCACTGGTTGGGGCATTGCCA
CCACCTGTCAGCTCCTTTCCGGGACTTTCGCTTTCCCCCTCCCTATTGCCACGGCGGAACTCATCGCCGCCTGCCTT
GCCCGCTGCTGGACAGGGGCTCGGCTGTTGGGCACTGACAATTCCGTGGTGTTGTCGGGGAAGCTGACGTCCTTT
CCATGGCTGCTCGCCTGTGTTGCCACCTGGATTCTGCGCGGGACGTCCTTCTGCTACGTCCCTTCGGCCCTCAATC
CAGCGGACCTTCCTTCCCGCGGCCTGCTGCCGGCTCTGCGGCCTCTTCCGCGTCTTCGCCTTCGCCCTCAGACGA
GTCGGATCTCCCTTTGGGCCGCCTCCCCGCCCAGGTTCCTTGAGCATCTGGAATTCTGCCTAATAAAAAACATTTATT
TTCATTGCAATGATGTATTTAAATTATTTCTGAATATTTTACTAAAAAGGGAATGTGGGAGGTCAGTGCATTTAAGACATA
AAGAAATGAAGAGCTAGTTCAAACCTTGGGAAAATACACTATATCTTAAACTCCATGAAAGAAGGTGAGGCTGCAAAC
AGCTAATGCACATTGGCAACAGCCCCTGATGCCTCTGCCTTATTCATCCCTCAGAAAAGGATTCAAGGCATAGTGTTA
CCATCAACCACCTTAACTTCATTTTTCTTATTCAATACCTAGGTAGGTAGATGCTAGATTCTGGAAATAAAATATGAGTCT
CAAGTGGTCCTTGTCCTCTCTCCCAGTCAAATTCTGAATCTAGTTGGCAAGATTCTGAAATCAAGGCATATAATCAGTA
ATAAGTGATGATAGAAGGGTATTTCCATAGGCTCCGCCCCCCTGACGAGCATCACAAAAATCGACGCTCAAGTCAGAG
GTGGCGAAACCCGACAGGACTATAAAGATACCAGGCGTTTCCCCCTGGAAGCTCCCTCGTGCGCTCTCCTGTTCCG
ACCCTGCCGCTTACCGGATACCTGTCCGCCTTTCTCCCTTCGGGAAGCGTGGCGCTTTCTCATAGCTCACGCTGTAG
GTATCTCAGTTCGGTGTAGGTCGTTCGCTCCAAGCTGGGCTGTGTGCACGAACCCCCCGTTCAGCCCGACCGCTGC
GCCTTATCCGGTAACTATCGTCTTGAGTCCAACCCGGTAAGACACGACTTATCGCCACTGGCAGCAGCCACTGGTAA
CAGGATTAGCAGAGCGAGGTATGTAGGCGGTGCTACAGAGTTCTTGAAGTGGTGGCCTAACTACGGCTACACTAGAA
GAACAGTATTTGGTATCTGCGCTCTGCTGAAGCCAGTTACCTTCGGAAAAAGAGTTGGTAGCTCTTGATCCGGCAAAC
AAACCACCGCTGGTAGCGGTGGTTTTTTTGTTTGCAAGCAGCAGATTACGCGCAGAAAAAAAGGATCTCAAGAAGAT
CCTTTGATCTTTTCTACGGGGTCTGACGCTCAGTGGAACGAAAACTCACGTTAAGGGATTTTGGTCATGAGATTATCA
AAAAGGATCTTCACCTAGATCCTTTTAAATTAAAAATGAAGTTTTAAATCAATCTAAAGTATATATGAGTAAACTTGGTCT
GACAGCGGCCGCAAATGCTAAACCACTGCAGTGGTTACCAGTGCTTGATCAGTGAGGCACCGATCTCAGCGATCTG
CCTATTTCGTTCGTCCATAGTGGCCTGACTCCCCGTCGTGTAGATCACTACGATTCGTGAGGGCTTACCATCAGGCCC
CAGCGCAGCAATGATGCCGCGAGAGCCGCGTTCACCGGCCCCCGATTTGTCAGCAATGAACCAGCCAGCAGGGAG
GGCCGAGCGAAGAAGTGGTCCTGCTACTTTGTCCGCCTCCATCCAGTCTATGAGCTGCTGTCGTGATGCTAGAGTAA
GAAGTTCGCCAGTGAGTAGTTTCCGAAGAGTTGTGGCCATTGCTACTGGCATCGTGGTATCACGCTCGTCGTTCGGT
ATGGCTTCGTTCAACTCTGGTTCCCAGCGGTCAAGCCGGGTCACATGATCACCCATATTATGAAGAAATGCAGTCAGC
TCCTTAGGGCCTCCGATCGTTGTCAGAAGTAAGTTGGCCGCGGTGTTGTCGCTCATGGTAATGGCAGCACTACACAA
TTCTCTTACCGTCATGCCATCCGTAAGATGCTTTTCCGTGACCGGCGAGTACTCAACCAAGTCGTTTTGTGAGTAGTG
TATACGGCGACCAAGCTGCTCTTGCCCGGCGTCTATACGGGACAACACCGCGCCACATAGCAGTACTTTGAAAGTGC
TCATCATCGGGAATCGTTCTTCGGGGCGGAAAGACTCAAGGATCTTGCCGCTATTGAGATCCAGTTCGATATAGCCCA
CTCTTGCACCCAGTTGATCTTCAGCATCTTTTACTTTCACCAGCGTTTCGGGGTGTGCAAAAACAGGCAAGCAAAATG
CCGCAAAGAAGGGAATGAGTGCGACACGAAAATGTTGGATGCTCATACTCGTCCTTTTTCAATATTATTGAAGCATTTA
TCAGGGTTACTAGTACGTCTCTCAAGGATAAGTAAGTAATATTAAGGTACGGGAGGTATTGGACAAGAGAAATGTTCTG
GCACCTGCACTTGCACTGGGGACAGCCTATTTTGCTAGTTTGTTTTGTTTCGTTTTGTTTTGATGGAGAGCGTATGTTA
GTACTATCGATTCACACAAAAAACCAACACACAGATGTAATGAAAATAAAGATATTTTATTGCGGCTGCATCTCAATTAG
TCAGCAACCATAGTCCCGCCCCTAACTCCGCCCATCCCGCCCCTAACTCCGCCCAGTTCCGCCCATTCTCCGCCCCA
TGGCTGACTAATTTTTTTTATTTATGCAGAGGCCGAGGCCGCCTCGGCCTCTGAGCTATTCCAGAAGTAGTGAGGAGG
CTTTTTTGGAGGCCTAGGCTTTTGCAAAAAGCTTTGCAAAGATGGATAAAGTTTTAAACAGAGAGGAATCTTTGCAGC
TAATGGACCTTCTAGGTCTTGAAAGGAGTGCGTGAG

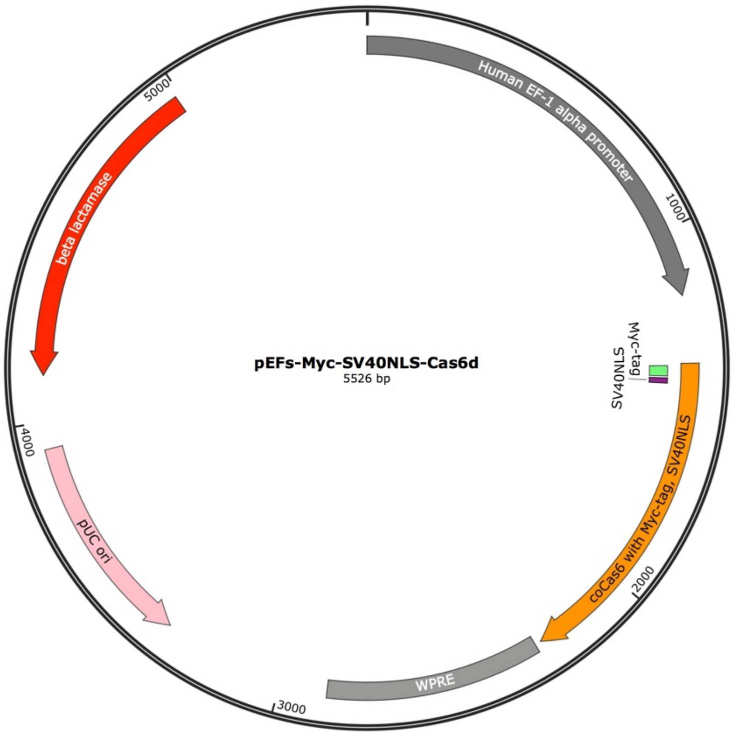

Supplementary Fig. 12. Plasmid vector pEFs-myc-SV40NLS-Cas6d.

1..1182: Human EF-1 alpha promoter
1370..2104: coCas5d with Strept-tag, SV40NLS
1375..1404: Myc-tag
1411..1425: SV40NLS
2283..2871: Woodchuck hepatitis virus posttranscriptional regulatory element (WPRE)
3338..3926(complement): pUC ori
4126..4986(complement): beta lactamase

>pEFs-Myc-SV40NLS-Cas6d
GCTCCGGTGCCCGTCAGTGGGCAGAGCGCACATCGCCCACAGTCCCCGAGAAGTTGGGGGGAGGGGTCGGCAAT
TGAACCGGTGCCTAGAGAAGGTGGCGCGGGGTAAACTGGGAAAGTGATGTCGTGTACTGGCTCCGCCTTTTTCCCG
AGGGTGGGGGAGAACCGTATATAAGTGCAGTAGTCGCCGTGAACGTTCTTTTTCGCAACGGGTTTGCCGCCAGAAC
ACAGGTAAGTGCCGTGTGTGGTTCCCGCGGGCCTGGCCTCTTTACGGGTTATGGCCCTTGCGTGCCTTGAATTACTT
CCACGCCCCTGGCTGCAGTACGTGATTCTTGATCCCGAGCTTCGGGTTGGAAGTGGGTGGGAGAGTTCGAGGCCTT
GCGCTTAAGGAGCCCCTTCGCCTCGTGCTTGAGTTGAGGCCTGGCCTGGGCGCTGGGGCCGCCGCGTGCGAATCT
GGTGGCACCTTCGCGCCTGTCTCGCTGCTTTCGATAAGTCTCTAGCCATTTAAAATTTTTGATGACCTGCTGCGACGC
TTTTTTTCTGGCAAGATAGTCTTGTAAATGCGGGCCAAGATCTGCACACTGGTATTTCGGTTTTTGGGGCCGCGGGC
GGCGACGGGGCCCGTGCGTCCCAGCGCACATGTTCGGCGAGGCGGGGCCTGCGAGCGCGGCCACCGAGAATCG
GACGGGGGTAGTCTCAAGCTGGCCGGCCTGCTCTGGTGCCTGGCCTCGCGCCGCCGTGTATCGCCCCGCCCTGG
GCGGCAAGGCTGGCCCGGTCGGCACCAGTTGCGTGAGCGGAAAGATGGCCGCTTCCCGGCCCTGCTGCAGGGAG
CTCAAAATGGAGGACGCGGCGCTCGGGAGAGCGGGCGGGTGAGTCACCCACACAAAGGAAAAGGGCCTTTCCGT
CCTCAGCCGTCGCTTCATGTGACTCCACGGAGTACCGGGCGCCGTCCAGGCACCTCGATTAGTTCTCGAGCTTTTG
GAGTACGTCGTCTTTAGGTTGGGGGGAGGGGTTTTATGCGATGGAGTTTCCCCACACTGAGTGGGTGGAGACTGAA
GTTAGGCCAGCTTGGCACTTGATGTAATTCTCCTTGGAATTTGCCCTTTTTGAGTTTGGATCTTGGTTCATTCTCAAGC
CTCAGACAGTGGTTCAAAGTTTTTTTCTTCCATTTCAGGTGTCGTGAATTGCGGAAGGCCGTCAAGGCCACGTGTCT
TGTCCAGAGCTCAGGTGTCGTGAATTGCGTAGCATTTAGGTGACACTATAGAACTCACCTATCTCCCCAACACCTAATA
ACATTCAATCACTCTTTCCACTAACCACCTATCTACATCACCACCCACCACCACCAGCCCGGGATATCAGCTGCACCA
TGGCGGAACAAAAACTCATCTCAGAAGAGGATCTGGACCCGAAAAAGAAGCGAAAGGTTTCTGGTCCTTACTCCCTG
GTCCTGAACCTGACCCCTCGCTCCCCTATCTACCCTAACTTCCTGACCGGCCGCCATCTGCATGCCCTGTTCCTGAC
CCTGGTTTCCTCCGTCGATCAAGAACTGGGCAACATCCTGCATACCGCCGAAGCCGATAAGGCCTTCACCCTGTCCC
CTCTGCAAATGCAATCCGGCGGCAAGACCATCAACTCCCCTCAATGGCGCCATGAACGCGAAATCGCCTCCGAAAC
CCCTTGCTGGTGGCGCATCTCCCTGCTGGATGATCGCCTGTTCGGCAAGCTGACCTCCCTGTGGCTGAACCTGAAC
CCTAAGCAACCTTGGCATCTGGGCTCCGCCGATCTGGTCATCACCTCCGTCCTGGCCACCCCTCAATCCGTCCAAC

CTTGGGCCAACTCCTGCACCTACCAATACCTGTACGAAAACGCCTCCGAAACCAACCGCGAGTTCGATTTCCTGTTC
GCCACCCCTGTCACCTTCCGCCAAGGCAAGTTCGATTCCGCCCTGCCTACCCGCGAACTGGTCTTCAACTCCCTGC
TGGGCCGCTGGAACCGCTACTCCGGCATCCCTTTCGATTCCATCGCCCTGGAATCCATCTTCCCTTCCTTCTTCGATA
TCCAAACCAAGCTGGCCGATGAAGCCTACAAGAACCAATCCATCGGCTGCGTCGGCGAAATCCATTACCGCCTGCTG
GGCGAAGTCGAACCTGCCAAGATCAAGGCCATCAACGCTCTGGCTGATTTCGCCCTGTACGCCGGAGTCGGCCGCA
AGACCACTATGGGCATGGGCATGACCCGCCGCATCTCCAAGGATAAGCGCTGATCTAGAAATTCATCGACAATCAACC
TCTGGATTACAAAATTTGTGAAAGATTGACTGGTATTCTTAACTATGTTGCTCCTTTTACGCTATGTGGATACGCTGCTT
TAATGCCTTTGTATCATGCTATTGCTTCCCGTATGGCTTTCATTTTCTCCTCCTTGTATAAATCCTGGTTGCTGTCTCTTT
ATGAGGAGTTGTGGCCCGTTGTCAGGCAACGTGGCGTGGTGTGCACTGTGTTTGCTGACGCAACCCCCACTGGTTG
GGGCATTGCCACCACCTGTCAGCTCCTTTCCGGGACTTTCGCTTTCCCCCTCCCTATTGCCACGGCGGAACTCATCG
CCGCCTGCCTTGCCCGCTGCTGGACAGGGGCTCGGCTGTTGGGCACTGACAATTCCGTGGTGTTGTCGGGGAAGC
TGACGTCCTTTCCATGGCTGCTCGCCTGTGTTGCCACCTGGATTCTGCGCGGGACGTCCTTCTGCTACGTCCCTTCG
GCCCTCAATCCAGCGGACCTTCCTTCCCGCGGCCTGCTGCCGGCTCTGCGGCCTCTTCCGCGTCTTCGCCTTCGC
CCTCAGACGAGTCGGATCTCCCTTTGGGCCGCCTCCCCGCCCAGGTTCCTTGAGCATCTGGAATTCTGCCTAATAAA
AAACATTTATTTTCATTGCAATGATGTATTTAAATTATTTCTGAATATTTTACTAAAAAGGGAATGTGGGAGGTCAGTGCA
TTTAAGACATAAAGAAATGAAGAGCTAGTTCAAACCTTGGGAAAATACACTATATCTTAAACTCCATGAAAGAAGGTGA
GGCTGCAAACAGCTAATGCACATTGGCAACAGCCCCTGATGCCTCTGCCTTATTCATCCCTCAGAAAAGGATTCAAG
GCATAGTGTTACCATCAACCACCTTAACTTCATTTTTCTTATTCAATACCTAGGTAGGTAGATGCTAGATTCTGGAAATAA
AATATGAGTCTCAAGTGGTCCTTGTCCTCTCTCCCAGTCAAATTCTGAATCTAGTTGGCAAGATTCTGAAATCAAGGCA
TATAATCAGTAATAAGTGATGATAGAAGGGTATTTCCATAGGCTCCGCCCCCCTGACGAGCATCACAAAAATCGACGCT
CAAGTCAGAGGTGGCGAAACCCGACAGGACTATAAAGATACCAGGCGTTTCCCCCTGGAAGCTCCCTCGTGCGCTC
TCCTGTTCCGACCCTGCCGCTTACCGGATACCTGTCCGCCTTTCTCCCTTCGGGAAGCGTGGCGCTTTCTCATAGCT
CACGCTGTAGGTATCTCAGTTCGGTGTAGGTCGTTCGCTCCAAGCTGGGCTGTGTGCACGAACCCCCCGTTCAGCC
CGACCGCTGCGCCTTATCCGGTAACTATCGTCTTGAGTCCAACCCGGTAAGACACGACTTATCGCCACTGGCAGCAG
CCACTGGTAACAGGATTAGCAGAGCGAGGTATGTAGGCGGTGCTACAGAGTTCTTGAAGTGGTGGCCTAACTACGGC
TACACTAGAAGAACAGTATTTGGTATCTGCGCTCTGCTGAAGCCAGTTACCTTCGGAAAAAGAGTTGGTAGCTCTTGA
TCCGGCAAACAAACCACCGCTGGTAGCGGTGGTTTTTTTGTTTGCAAGCAGCAGATTACGCGCAGAAAAAAAGGATC
TCAAGAAGATCCTTTGATCTTTTCTACGGGGTCTGACGCTCAGTGGAACGAAAACTCACGTTAAGGGATTTTGGTCAT
GAGATTATCAAAAAGGATCTTCACCTAGATCCTTTTAAATTAAAAATGAAGTTTTAAATCAATCTAAAGTATATATGAGTAA
ACTTGGTCTGACAGCGGCCGCAAATGCTAAACCACTGCAGTGGTTACCAGTGCTTGATCAGTGAGGCACCGATCTCA
GCGATCTGCCTATTTCGTTCGTCCATAGTGGCCTGACTCCCCGTCGTGTAGATCACTACGATTCGTGAGGGCTTACCA
TCAGGCCCCAGCGCAGCAATGATGCCGCGAGAGCCGCGTTCACCGGCCCCCGATTTGTCAGCAATGAACCAGCCA
GCAGGGAGGGCCGAGCGAAGAAGTGGTCCTGCTACTTTGTCCGCCTCCATCCAGTCTATGAGCTGCTGTCGTGATG
CTAGAGTAAGAAGTTCGCCAGTGAGTAGTTTCCGAAGAGTTGTGGCCATTGCTACTGGCATCGTGGTATCACGCTCG
TCGTTCGGTATGGCTTCGTTCAACTCTGGTTCCCAGCGGTCAAGCCGGGTCACATGATCACCCATATTATGAAGAAAT
GCAGTCAGCTCCTTAGGGCCTCCGATCGTTGTCAGAAGTAAGTTGGCCGCGGTGTTGTCGCTCATGGTAATGGCAG
CACTACACAATTCTCTTACCGTCATGCCATCCGTAAGATGCTTTTCCGTGACCGGCGAGTACTCAACCAAGTCGTTTT
GTGAGTAGTGTATACGGCGACCAAGCTGCTCTTGCCCGGCGTCTATACGGGACAACACCGCGCCACATAGCAGTACT
TTGAAAGTGCTCATCATCGGGAATCGTTCTTCGGGGCGGAAAGACTCAAGGATCTTGCCGCTATTGAGATCCAGTTC
GATATAGCCCACTCTTGCACCCAGTTGATCTTCAGCATCTTTTACTTTCACCAGCGTTTCGGGGTGTGCAAAAACAGG
CAAGCAAAATGCCGCAAAGAAGGGAATGAGTGCGACACGAAAATGTTGGATGCTCATACTCGTCCTTTTTCAATATTAT
TGAAGCATTTATCAGGGTTACTAGTACGTCTCTCAAGGATAAGTAAGTAATATTAAGGTACGGGAGGTATTGGACAAGA
GAAATGTTCTGGCACCTGCACTTGCACTGGGGACAGCCTATTTTGCTAGTTTGTTTTGTTTCGTTTTGTTTTGATGGA
GAGCGTATGTTAGTACTATCGATTCACACAAAAAACCAACACACAGATGTAATGAAAATAAAGATATTTTATTGCGGCTG
CATCTCAATTAGTCAGCAACCATAGTCCCGCCCCTAACTCCGCCCATCCCGCCCCTAACTCCGCCCAGTTCCGCCCA
TTCTCCGCCCCATGGCTGACTAATTTTTTTTATTTATGCAGAGGCCGAGGCCGCCTCGGCCTCTGAGCTATTCCAGAA
GTAGTGAGGAGGCTTTTTTGGAGGCCTAGGCTTTTGCAAAAAGCTTTGCAAAGATGGATAAAGTTTTAAACAGAGAGG
AATCTTTGCAGCTAATGGACCTTCTAGGTCTTGAAAGGAGTGCGTGAG

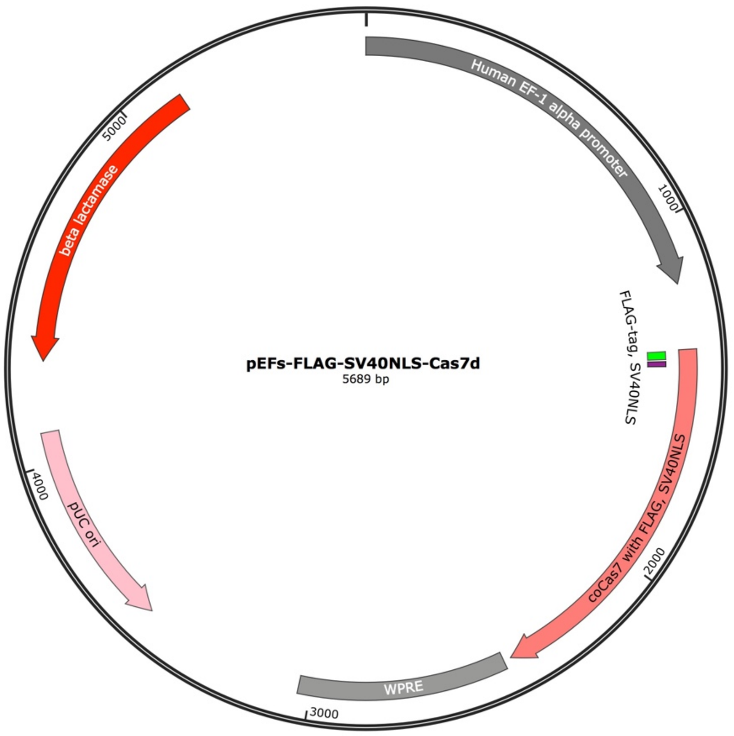

Supplementary Fig. 13. Plasmid vector pEFs-FLAG-SV40NLS-Cas7d.

1..1182: Human EF-1 alpha promoter
1369..2424: coCas7d with FLAG-tag, SV40NLS
1372..1395: FLAG-tag
1402..1416: SV40NLS
2446..3034: Woodchuck hepatitis virus posttranscriptional regulatory element (WPRE)
3501..4089(complement): pUC ori
4289..5149(complement): beta lactamase

>pEFs-FLAG-SV40NLS-Cas7d
GCTCCGGTGCCCGTCAGTGGGCAGAGCGCACATCGCCCACAGTCCCCGAGAAGTTGGGGGGAGGGGTCGGCAAT
TGAACCGGTGCCTAGAGAAGGTGGCGCGGGGTAAACTGGGAAAGTGATGTCGTGTACTGGCTCCGCCTTTTTCCCG
AGGGTGGGGGAGAACCGTATATAAGTGCAGTAGTCGCCGTGAACGTTCTTTTTCGCAACGGGTTTGCCGCCAGAAC
ACAGGTAAGTGCCGTGTGTGGTTCCCGCGGGCCTGGCCTCTTTACGGGTTATGGCCCTTGCGTGCCTTGAATTACTT
CCACGCCCCTGGCTGCAGTACGTGATTCTTGATCCCGAGCTTCGGGTTGGAAGTGGGTGGGAGAGTTCGAGGCCTT
GCGCTTAAGGAGCCCCTTCGCCTCGTGCTTGAGTTGAGGCCTGGCCTGGGCGCTGGGGCCGCCGCGTGCGAATCT
GGTGGCACCTTCGCGCCTGTCTCGCTGCTTTCGATAAGTCTCTAGCCATTTAAAATTTTTGATGACCTGCTGCGACGC
TTTTTTTCTGGCAAGATAGTCTTGTAAATGCGGGCCAAGATCTGCACACTGGTATTTCGGTTTTTGGGGCCGCGGGC
GGCGACGGGGCCCGTGCGTCCCAGCGCACATGTTCGGCGAGGCGGGGCCTGCGAGCGCGGCCACCGAGAATCG
GACGGGGGTAGTCTCAAGCTGGCCGGCCTGCTCTGGTGCCTGGCCTCGCGCCGCCGTGTATCGCCCCGCCCTGG
GCGGCAAGGCTGGCCCGGTCGGCACCAGTTGCGTGAGCGGAAAGATGGCCGCTTCCCGGCCCTGCTGCAGGGAG
CTCAAAATGGAGGACGCGGCGCTCGGGAGAGCGGGCGGGTGAGTCACCCACACAAAGGAAAAGGGCCTTTCCGT
CCTCAGCCGTCGCTTCATGTGACTCCACGGAGTACCGGGCGCCGTCCAGGCACCTCGATTAGTTCTCGAGCTTTTG
GAGTACGTCGTCTTTAGGTTGGGGGGAGGGGTTTTATGCGATGGAGTTTCCCCACACTGAGTGGGTGGAGACTGAA
GTTAGGCCAGCTTGGCACTTGATGTAATTCTCCTTGGAATTTGCCCTTTTTGAGTTTGGATCTTGGTTCATTCTCAAGC
CTCAGACAGTGGTTCAAAGTTTTTTTCTTCCATTTCAGGTGTCGTGAATTGCGGAAGGCCGTCAAGGCCACGTGTCT
TGTCCAGAGCTCAGGTGTCGTGAATTGCGTAGCATTTAGGTGACACTATAGAACTCACCTATCTCCCCAACACCTAATA
ACATTCAATCACTCTTTCCACTAACCACCTATCTACATCACCACCCACCACCACCAGCCCGGGATATCAGCTGCACCA
TGGACTACAAGGATGACGATGACAAGGATCCAAAGAAGAAACGCAAAGTCTCCGGAACCTTCCTGACCTCCGTCGAT
GCCAAGTTCTTCCATTCCGAAATCCCTTACAAGCCTATGGGCAAGTACGTCCATTTCCTGACCATCCGCGTCACCGAA
TCCTACCCTCTGTTCCAAACCGATGGCGAACTGAACAAGGCCCGCGTCCGCGCCGGCATCGATTCCAAGAAGACCA
TCTCCCGCCTGTCCATGTTCAAGCGCAAGCAATCCACCCCTGAACGCCTGGTCGGCCGCGAACTGCTGCGCAACTA
CGGCCTGATCACCGCCGAAGAATGCGAATACAACGTCAAGTTCGCCATGAACAACGCCGATTGCATCATCTACGGCT
TCGCCATCGGCGATTCCGGCTCCGAAAAGTCCAAGGTCGTCGTCGATACCGCCTTCTCCATCACCCCTTTCGATGAA

TCCCATGAATCCTTCACACTGAATGCTCCTTACGAAAACGGCACTATGGCCTCCAAGGGCGAAAACAACACCAAGGT
CGGCGAAGTCACCTCCCGCATCAACCAACAAGATCATATCCGCCCTCAAGTCTTCTTCCCTTCCATCGTCACCCTGAA
GGACCCTACCGAAGCCTCCTTCCTGTACGTCTTCAACAACATCCTGCGCACCCGCCATTACGGCGCCCAAACCACC
CGCACCGGCCGCGTCCGCAACGAACTGATCGGCGTCATCTTCGCCGATGGCGAAATCGTCTCCAACCTGCGCTGG
ACCCAAGCCATCTACGATCGCCTGCCTGATGAAGTCCTGCATTCCATCGATCCTCTGGATGAAGATCTGGTCATGGAA
AAGGCCACCGAAGCCATCCAAGCCCTGATGGCCGAAGAGTTCATCGTCCATACCGATTTCATCGGCGAAAACTTCCA
ACCTCTGCTGACCGAAGTCAAGACCCTGACCGGCACCGAAGCCGGCATCCTGTCCGTCCTGGATCAAGCCAACAAG
GAATCCAAGAAGTACTTCGAACAATACATCGAAAAGAAGAAGGCCGAAAAGAAGTAGTGATTCTAGAAATTCATCGAC
AATCAACCTCTGGATTACAAAATTTGTGAAAGATTGACTGGTATTCTTAACTATGTTGCTCCTTTTACGCTATGTGGATA
CGCTGCTTTAATGCCTTTGTATCATGCTATTGCTTCCCGTATGGCTTTCATTTTCTCCTCCTTGTATAAATCCTGGTTGC
TGTCTCTTTATGAGGAGTTGTGGCCCGTTGTCAGGCAACGTGGCGTGGTGTGCACTGTGTTTGCTGACGCAACCCC
CACTGGTTGGGGCATTGCCACCACCTGTCAGCTCCTTTCCGGGACTTTCGCTTTCCCCCTCCCTATTGCCACGGCG
GAACTCATCGCCGCCTGCCTTGCCCGCTGCTGGACAGGGGCTCGGCTGTTGGGCACTGACAATTCCGTGGTGTTG
TCGGGGAAGCTGACGTCCTTTCCATGGCTGCTCGCCTGTGTTGCCACCTGGATTCTGCGCGGGACGTCCTTCTGCT
ACGTCCCTTCGGCCCTCAATCCAGCGGACCTTCCTTCCCGCGGCCTGCTGCCGGCTCTGCGGCCTCTTCCGCGTC
TTCGCCTTCGCCCTCAGACGAGTCGGATCTCCCTTTGGGCCGCCTCCCCGCCCAGGTTCCTTGAGCATCTGGAATT
CTGCCTAATAAAAAACATTTATTTTCATTGCAATGATGTATTTAAATTATTTCTGAATATTTTACTAAAAAGGGAATGTGGG
AGGTCAGTGCATTTAAGACATAAAGAAATGAAGAGCTAGTTCAAACCTTGGGAAAATACACTATATCTTAAACTCCATGA
AAGAAGGTGAGGCTGCAAACAGCTAATGCACATTGGCAACAGCCCCTGATGCCTCTGCCTTATTCATCCCTCAGAAA
AGGATTCAAGGCATAGTGTTACCATCAACCACCTTAACTTCATTTTTCTTATTCAATACCTAGGTAGGTAGATGCTAGAT
TCTGGAAATAAAATATGAGTCTCAAGTGGTCCTTGTCCTCTCTCCCAGTCAAATTCTGAATCTAGTTGGCAAGATTCTG
AAATCAAGGCATATAATCAGTAATAAGTGATGATAGAAGGGTATTTCCATAGGCTCCGCCCCCCTGACGAGCATCACAA
AAATCGACGCTCAAGTCAGAGGTGGCGAAACCCGACAGGACTATAAAGATACCAGGCGTTTCCCCCTGGAAGCTCC
CTCGTGCGCTCTCCTGTTCCGACCCTGCCGCTTACCGGATACCTGTCCGCCTTTCTCCCTTCGGGAAGCGTGGCGC
TTTCTCATAGCTCACGCTGTAGGTATCTCAGTTCGGTGTAGGTCGTTCGCTCCAAGCTGGGCTGTGTGCACGAACCC
CCCGTTCAGCCCGACCGCTGCGCCTTATCCGGTAACTATCGTCTTGAGTCCAACCCGGTAAGACACGACTTATCGCC
ACTGGCAGCAGCCACTGGTAACAGGATTAGCAGAGCGAGGTATGTAGGCGGTGCTACAGAGTTCTTGAAGTGGTGG
CCTAACTACGGCTACACTAGAAGAACAGTATTTGGTATCTGCGCTCTGCTGAAGCCAGTTACCTTCGGAAAAAGAGTT
GGTAGCTCTTGATCCGGCAAACAAACCACCGCTGGTAGCGGTGGTTTTTTTGTTTGCAAGCAGCAGATTACGCGCAG
AAAAAAAGGATCTCAAGAAGATCCTTTGATCTTTTCTACGGGGTCTGACGCTCAGTGGAACGAAAACTCACGTTAAGG
GATTTTGGTCATGAGATTATCAAAAAGGATCTTCACCTAGATCCTTTTAAATTAAAAATGAAGTTTTAAATCAATCTAAAG
TATATATGAGTAAACTTGGTCTGACAGCGGCCGCAAATGCTAAACCACTGCAGTGGTTACCAGTGCTTGATCAGTGAG
GCACCGATCTCAGCGATCTGCCTATTTCGTTCGTCCATAGTGGCCTGACTCCCCGTCGTGTAGATCACTACGATTCGT
GAGGGCTTACCATCAGGCCCCAGCGCAGCAATGATGCCGCGAGAGCCGCGTTCACCGGCCCCCGATTTGTCAGCA
ATGAACCAGCCAGCAGGGAGGGCCGAGCGAAGAAGTGGTCCTGCTACTTTGTCCGCCTCCATCCAGTCTATGAGCT
GCTGTCGTGATGCTAGAGTAAGAAGTTCGCCAGTGAGTAGTTTCCGAAGAGTTGTGGCCATTGCTACTGGCATCGTG
GTATCACGCTCGTCGTTCGGTATGGCTTCGTTCAACTCTGGTTCCCAGCGGTCAAGCCGGGTCACATGATCACCCAT
ATTATGAAGAAATGCAGTCAGCTCCTTAGGGCCTCCGATCGTTGTCAGAAGTAAGTTGGCCGCGGTGTTGTCGCTCA
TGGTAATGGCAGCACTACACAATTCTCTTACCGTCATGCCATCCGTAAGATGCTTTTCCGTGACCGGCGAGTACTCAA
CCAAGTCGTTTTGTGAGTAGTGTATACGGCGACCAAGCTGCTCTTGCCCGGCGTCTATACGGGACAACACCGCGCCA
CATAGCAGTACTTTGAAAGTGCTCATCATCGGGAATCGTTCTTCGGGGCGGAAAGACTCAAGGATCTTGCCGCTATTG
AGATCCAGTTCGATATAGCCCACTCTTGCACCCAGTTGATCTTCAGCATCTTTTACTTTCACCAGCGTTTCGGGGTGT
GCAAAAACAGGCAAGCAAAATGCCGCAAAGAAGGGAATGAGTGCGACACGAAAATGTTGGATGCTCATACTCGTCCT
TTTTCAATATTATTGAAGCATTTATCAGGGTTACTAGTACGTCTCTCAAGGATAAGTAAGTAATATTAAGGTACGGGAGGT
ATTGGACAAGAGAAATGTTCTGGCACCTGCACTTGCACTGGGGACAGCCTATTTTGCTAGTTTGTTTTGTTTCGTTTT
GTTTTGATGGAGAGCGTATGTTAGTACTATCGATTCACACAAAAAACCAACACACAGATGTAATGAAAATAAAGATATTT
TATTGCGGCTGCATCTCAATTAGTCAGCAACCATAGTCCCGCCCCTAACTCCGCCCATCCCGCCCCTAACTCCGCCC
AGTTCCGCCCATTCTCCGCCCCATGGCTGACTAATTTTTTTTATTTATGCAGAGGCCGAGGCCGCCTCGGCCTCTGA
GCTATTCCAGAAGTAGTGAGGAGGCTTTTTTGGAGGCCTAGGCTTTTGCAAAAAGCTTTGCAAAGATGGATAAAGTTT
TAAACAGAGAGGAATCTTTGCAGCTAATGGACCTTCTAGGTCTTGAAAGGAGTGCGTGAG

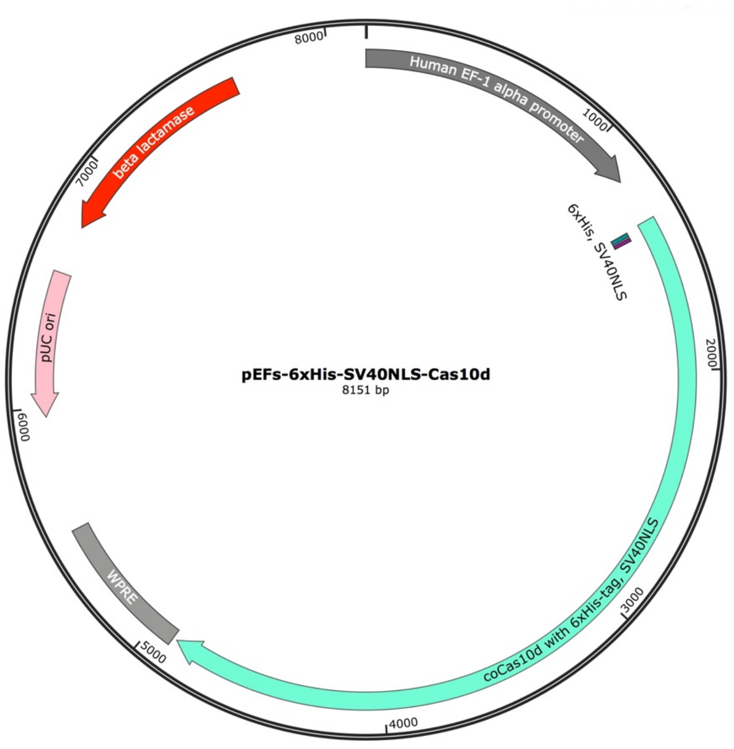

Supplementary Fig. 14. Plasmid vector pEFs-6xHis-SV40NLS-Cas10d.

1..1182: Human EF-1 alpha promoter
1369..4890: coCas10d with 6xHis-tag, SV40NLS
1375..1392: 6xHis-tag
1399..1413: SV40NLS
4908..5496: Woodchuck hepatitis virus posttranscriptional regulatory element (WPRE)
5963..6551(complement): pUC ori
6751..7611(complement): beta lactamase

>pEFs-6xHis-SV40NLS-Cas10d
GCTCCGGTGCCCGTCAGTGGGCAGAGCGCACATCGCCCACAGTCCCCGAGAAGTTGGGGGGAGGGGTCGGCAAT
TGAACCGGTGCCTAGAGAAGGTGGCGCGGGGTAAACTGGGAAAGTGATGTCGTGTACTGGCTCCGCCTTTTTCCCG
AGGGTGGGGGAGAACCGTATATAAGTGCAGTAGTCGCCGTGAACGTTCTTTTTCGCAACGGGTTTGCCGCCAGAAC
ACAGGTAAGTGCCGTGTGTGGTTCCCGCGGGCCTGGCCTCTTTACGGGTTATGGCCCTTGCGTGCCTTGAATTACTT
CCACGCCCCTGGCTGCAGTACGTGATTCTTGATCCCGAGCTTCGGGTTGGAAGTGGGTGGGAGAGTTCGAGGCCTT
GCGCTTAAGGAGCCCCTTCGCCTCGTGCTTGAGTTGAGGCCTGGCCTGGGCGCTGGGGCCGCCGCGTGCGAATCT
GGTGGCACCTTCGCGCCTGTCTCGCTGCTTTCGATAAGTCTCTAGCCATTTAAAATTTTTGATGACCTGCTGCGACGC
TTTTTTTCTGGCAAGATAGTCTTGTAAATGCGGGCCAAGATCTGCACACTGGTATTTCGGTTTTTGGGGCCGCGGGC
GGCGACGGGGCCCGTGCGTCCCAGCGCACATGTTCGGCGAGGCGGGGCCTGCGAGCGCGGCCACCGAGAATCG
GACGGGGGTAGTCTCAAGCTGGCCGGCCTGCTCTGGTGCCTGGCCTCGCGCCGCCGTGTATCGCCCCGCCCTGG
GCGGCAAGGCTGGCCCGGTCGGCACCAGTTGCGTGAGCGGAAAGATGGCCGCTTCCCGGCCCTGCTGCAGGGAG
CTCAAAATGGAGGACGCGGCGCTCGGGAGAGCGGGCGGGTGAGTCACCCACACAAAGGAAAAGGGCCTTTCCGT
CCTCAGCCGTCGCTTCATGTGACTCCACGGAGTACCGGGCGCCGTCCAGGCACCTCGATTAGTTCTCGAGCTTTTG
GAGTACGTCGTCTTTAGGTTGGGGGGAGGGGTTTTATGCGATGGAGTTTCCCCACACTGAGTGGGTGGAGACTGAA
GTTAGGCCAGCTTGGCACTTGATGTAATTCTCCTTGGAATTTGCCCTTTTTGAGTTTGGATCTTGGTTCATTCTCAAGC
CTCAGACAGTGGTTCAAAGTTTTTTTCTTCCATTTCAGGTGTCGTGAATTGCGGAAGGCCGTCAAGGCCACGTGTCT
TGTCCAGAGCTCAGGTGTCGTGAATTGCGTAGCATTTAGGTGACACTATAGAACTCACCTATCTCCCCAACACCTAATA
ACATTCAATCACTCTTTCCACTAACCACCTATCTACATCACCACCCACCACCACCAGCCCGGGATATCAGCTGCACCA
TGGCGCATCACCATCACCATCACGACCCTAAGAAGAAGCGCAAGGTTTCTGGACCTAAGAAGCAAAAGAAGCTGGAA
GAAACCGGCCAACTGAACCTGTTCGATAACACCACCGAAATCGATGATGAAGATCTGGATTTCGAGTTCGAAGATATC
GATCTGGAATCCCTGGTGTCCGAAGATCTGGGCATCACCGAATCCGTCTCCGATCGCCGCGTCGAAACCGTCCGCC
AACTGCTGACCCTGAAGCTGCTGCGCGAAGCCATCCGCGCCGAAAACCCTGATGATCGCGTCATGGCCGATTTCGC
CGAAATGGTCCTGCCTAACCTGCTGCGCCTGGCCATCGGCGTCACCGCCAAGGGCGGCAACTTCATCGAAGCCGT
CGATCGCGGCCGCGAACTGCGCAACAAGCCTAAGGCCAAGCGCGATAACGCCGGCGATCAATCCCTGAACACCCAT

CTGCTGAACGGCCTGTTCCCTGCCAACCTGATCGAAAAGCGCCTGCAAAAGCTGAACACCACCGTCCGCCGCATCA
TCAAGGAGTTCGAACGCCGCCTGGCCATCGCCGGCTTCCTGGTCCATGATTTCGAAAAGTTCTCCTACGATCGCTTC
CCTTCCATGTCCGAACGCTACATCCAAATCCAACGCGATTTCATCCAAGATCCTTTCAAGAACCAAGATCCTCGCAAG
CTGTCCCGCGAAGAACATCGCGAAATCCTGCAAGTCCTGATCCCTGAACTGGGCCTGGATCGCTTCCTGTTCCCTGA
TAACCCTGAACGCTGGCTGGAATACCTGGATGATCTGCTGTATATCGCCAAGAACACCCAACGCCGCAACGATACCG
ATCTGAACACCTCCGAAGATGGCCTCAATGTCCGCCTGAACGATCGCGTCATCGAATCCCTGTGCGATCTGGCTTGC
CTGGCTGATCGCCTGGCCTCTATCATCAAGCATCCTCATGATGCCGAAAAGGCCTCCCTGCAAGATCTGCTGTACTC
CCTGTCCGATGGCGAACTGAAGTTCACCTACCATTCCATCGCCGAAAACCGCGGCGTCCTGACCAACGTCCTGAAC
AACGCCGTCATGGAAGCCCATCAAGAACTGGATTACCAACCTCTGCTGTACCTGCCTACCGGCGTCGTCTACATCGC
CCCTAAGAACGCCCCTGAAGTCTCCCTGGAAACCCTGCCTAACCGCGTCGTCGATACCATCAAGTCCCTGTGCTCC
GGCGAACTGCAACGCAAGCAAACCGGCTTCGGCCGCGATGGCAAGGGCATGAAGTACGCCGATTACTACTCCCAAT
TCTTCGATGATGCCGGCCTGATGCGCGCCGCCCTGAACGCCACCCTGCGCATCCTGGGCGATAACAAGGCCTCCGT
CGCCCGCTCCCGCGGCGAAAACCTGATCAAGTTCCAACAACAAGGCGTCCTGCCTACCGATTACGATTTCCATTGCG
AAGATGATATCCGCATCGATCGCCTGGCCGAGTTCGGCGATGTCGTCACCCGCAAGATCTGGGGCGATCGCCTGGA
AAAGATCGAACAAGCCCGCAAGCTGCAAAAGAACCTGCCTGCCCCTCCTGATCTGGATCTGATCTCCGAAATCGCCC
ATTACTGGAACCTGGAAAACTACCTGCCTCAAATCCGCGCCATCAAGCGCATCAACGAATCCCTGAAGGAACTGAAG
CTGAAGGGCAACACCGGCGGCGTCCCTTACGAATGGTACTACCTGGCCGCCCAATACCTGAAGCAACATCCTGGCA
TCGAAGATATCCGCCCTGTCGCCGAAGATCTGATCGCCTTCCTGGCCGCCAAGATCGCCGCCATCGTCGCCGGCTA
CAACCTGCCTGATGGCTGGGAAGATCTGCGCGAATGGGTCAACCAAGTCGTCCAACTGCCTGGCCGCGAACTGGC
CCATTCCATCGAAACCTTCCAAAAGGAACTGAACCATTACAACGCCGCCAAGAAGCAAGGCCGCGGCCGCCAACTG
CTGTGCTCCATCTCCCATTCCCCTTACTCCGTCTCCGAACAAATGGAATCCGCCGTCCTGTTCACCCCTCAAGTCTAC
ACCAACAAGCAAATGCTGGCCGGCTCCAACGCCAAGCGCAACATCTCCTCCATCGCCGGCACCGAAATGATGCTGC
GCCAAATCCTGATGAACCAAACCCAAGCCGTCGGCAAGCGCTTCGAAGATGGCAAGTACCGCTACCTGTACTTCTAC
CCTACCTACTACTTCACCCCTGAAACCAACTCCTTCCTGCAAAAGGCCTACGCCAACATCGCCCAAACCCGCTTCGAT
TCCTCCATCAAGCTGCATTTCGTCGATAAGAACCTGGTCGCCAACTTCGATCGCACCCGCTACCAATCCGTCGATTCC
TTCCTGATCGATGAAAAGCTGCGCCAAAAGAAGGAAACCATCAACGAAGAAGAAGATGGCAAGAAGGATCGCACCTT
CAAGCTGTCCTACCCTGAAGATAAGCCTCTGACCTTCTACTTCATGGCCCTGCCTCCTGGCCGCAACCCTACCGATA
CCGAATCCTGGGTCATGCCTGCCTGGCTGGGCCTGGCCTTCCCTATGATCCTGGATGTCAAGACCGTCGTCTCCGA
ATCCCCTATCCCTCCTTACCGCGATGGCGCCGAGTTCGAAGAAACCGTCTTCCTGGATTCCGCCCCTCAAGCCATCC
GCTCCCTGACCCGCTGCGATCGCTTCCGCCTGGATCGCGTCCTGAACCCTTGGCAAGATAACGATGGCAAGAAGTA
CTCCGCCCCTCTGAACACCCTGACCGCCGCCTACTCCATCCATCTGGATGTCAACTCCAAGCAAGGCAAGACCGGC
TACGATCCTAACTGGGGCAAGCTGACCGAACTGGCCATCAACCTGGAAACCTCCCCTCTGTACGTCTTCCATTACTTC
AAGCAATGGAAGCGCGGCAAGGATGCCGATATCCCTTCCGCCAACCGCATCGCCCTGTACCTGTACGATTTCTACCC
TTGCTTCGATCCTTACGTCCAAGCCAACCGCACCAACCTGACCATCGATATGACCGCCGAATCCCCTCTGAACCATC
CTAAGAACCTGACCGAACTGTACCGCCAATTCTACCGCGCCAAGTCCTCCAAGGGCAAGCCTATCAAGGCCAACGC
CATCCTGAAGCCTATCGATGAAGCCGCCGATATCATCCTGAAGGCCGATAAGGCCATCTCCGATGATCTGACCTCCCT
GGTCGCCGCCCGCCTGTTCAAGCTGATGGATCGCGTCCGCTCCCAAACCGCCGAAGGCCGCTACGTCATCAAGGA
ACGCGATCAAGAACGCGAAAAGATCCTGGATTTCGCCAAGTACTTCGTCAAGAACGTCTTCGAAGAATCCTTCGAATC
CGATCGCGCCCGCCTGGCCGGCCGCCAACTGAACATCATCCGCGATACCTGCGAGTTCCTGTACCGCCTGGAAATG
GATAAGGAACGCCGCCAACGCCAAGTCCAACCTCTGGATACCTCCAACTCCTCCTCCGAAGAAGAAGAATAGTCTAG
AAATTCATCGACAATCAACCTCTGGATTACAAAATTTGTGAAAGATTGACTGGTATTCTTAACTATGTTGCTCCTTTTAC
GCTATGTGGATACGCTGCTTTAATGCCTTTGTATCATGCTATTGCTTCCCGTATGGCTTTCATTTTCTCCTCCTTGTATAA
ATCCTGGTTGCTGTCTCTTTATGAGGAGTTGTGGCCCGTTGTCAGGCAACGTGGCGTGGTGTGCACTGTGTTTGCTG
ACGCAACCCCCACTGGTTGGGGCATTGCCACCACCTGTCAGCTCCTTTCCGGGACTTTCGCTTTCCCCCTCCCTATT
GCCACGGCGGAACTCATCGCCGCCTGCCTTGCCCGCTGCTGGACAGGGGCTCGGCTGTTGGGCACTGACAATTCC
GTGGTGTTGTCGGGGAAGCTGACGTCCTTTCCATGGCTGCTCGCCTGTGTTGCCACCTGGATTCTGCGCGGGACGT
CCTTCTGCTACGTCCCTTCGGCCCTCAATCCAGCGGACCTTCCTTCCCGCGGCCTGCTGCCGGCTCTGCGGCCTCT
TCCGCGTCTTCGCCTTCGCCCTCAGACGAGTCGGATCTCCCTTTGGGCCGCCTCCCCGCCCAGGTTCCTTGAGCAT
CTGGAATTCTGCCTAATAAAAAACATTTATTTTCATTGCAATGATGTATTTAAATTATTTCTGAATATTTTACTAAAAAGGG
AATGTGGGAGGTCAGTGCATTTAAGACATAAAGAAATGAAGAGCTAGTTCAAACCTTGGGAAAATACACTATATCTTAA
ACTCCATGAAAGAAGGTGAGGCTGCAAACAGCTAATGCACATTGGCAACAGCCCCTGATGCCTCTGCCTTATTCATC
CCTCAGAAAAGGATTCAAGGCATAGTGTTACCATCAACCACCTTAACTTCATTTTTCTTATTCAATACCTAGGTAGGTAG
ATGCTAGATTCTGGAAATAAAATATGAGTCTCAAGTGGTCCTTGTCCTCTCTCCCAGTCAAATTCTGAATCTAGTTGGC
AAGATTCTGAAATCAAGGCATATAATCAGTAATAAGTGATGATAGAAGGGTATTTCCATAGGCTCCGCCCCCCTGACGA
GCATCACAAAAATCGACGCTCAAGTCAGAGGTGGCGAAACCCGACAGGACTATAAAGATACCAGGCGTTTCCCCCTG
GAAGCTCCCTCGTGCGCTCTCCTGTTCCGACCCTGCCGCTTACCGGATACCTGTCCGCCTTTCTCCCTTCGGGAAG
CGTGGCGCTTTCTCATAGCTCACGCTGTAGGTATCTCAGTTCGGTGTAGGTCGTTCGCTCCAAGCTGGGCTGTGTGC
ACGAACCCCCCGTTCAGCCCGACCGCTGCGCCTTATCCGGTAACTATCGTCTTGAGTCCAACCCGGTAAGACACGA
CTTATCGCCACTGGCAGCAGCCACTGGTAACAGGATTAGCAGAGCGAGGTATGTAGGCGGTGCTACAGAGTTCTTGA
AGTGGTGGCCTAACTACGGCTACACTAGAAGAACAGTATTTGGTATCTGCGCTCTGCTGAAGCCAGTTACCTTCGGAA

AAAGAGTTGGTAGCTCTTGATCCGGCAAACAAACCACCGCTGGTAGCGGTGGTTTTTTTGTTTGCAAGCAGCAGATT
ACGCGCAGAAAAAAAGGATCTCAAGAAGATCCTTTGATCTTTTCTACGGGGTCTGACGCTCAGTGGAACGAAAACTC
ACGTTAAGGGATTTTGGTCATGAGATTATCAAAAAGGATCTTCACCTAGATCCTTTTAAATTAAAAATGAAGTTTTAAATC
AATCTAAAGTATATATGAGTAAACTTGGTCTGACAGCGGCCGCAAATGCTAAACCACTGCAGTGGTTACCAGTGCTTG
ATCAGTGAGGCACCGATCTCAGCGATCTGCCTATTTCGTTCGTCCATAGTGGCCTGACTCCCCGTCGTGTAGATCACT
ACGATTCGTGAGGGCTTACCATCAGGCCCCAGCGCAGCAATGATGCCGCGAGAGCCGCGTTCACCGGCCCCCGATT
TGTCAGCAATGAACCAGCCAGCAGGGAGGGCCGAGCGAAGAAGTGGTCCTGCTACTTTGTCCGCCTCCATCCAGTC
TATGAGCTGCTGTCGTGATGCTAGAGTAAGAAGTTCGCCAGTGAGTAGTTTCCGAAGAGTTGTGGCCATTGCTACTG
GCATCGTGGTATCACGCTCGTCGTTCGGTATGGCTTCGTTCAACTCTGGTTCCCAGCGGTCAAGCCGGGTCACATGA
TCACCCATATTATGAAGAAATGCAGTCAGCTCCTTAGGGCCTCCGATCGTTGTCAGAAGTAAGTTGGCCGCGGTGTTG
TCGCTCATGGTAATGGCAGCACTACACAATTCTCTTACCGTCATGCCATCCGTAAGATGCTTTTCCGTGACCGGCGAG
TACTCAACCAAGTCGTTTTGTGAGTAGTGTATACGGCGACCAAGCTGCTCTTGCCCGGCGTCTATACGGGACAACAC
CGCGCCACATAGCAGTACTTTGAAAGTGCTCATCATCGGGAATCGTTCTTCGGGGCGGAAAGACTCAAGGATCTTGC
CGCTATTGAGATCCAGTTCGATATAGCCCACTCTTGCACCCAGTTGATCTTCAGCATCTTTTACTTTCACCAGCGTTTC
GGGGTGTGCAAAAACAGGCAAGCAAAATGCCGCAAAGAAGGGAATGAGTGCGACACGAAAATGTTGGATGCTCATA
CTCGTCCTTTTTCAATATTATTGAAGCATTTATCAGGGTTACTAGTACGTCTCTCAAGGATAAGTAAGTAATATTAAGGTA
CGGGAGGTATTGGACAAGAGAAATGTTCTGGCACCTGCACTTGCACTGGGGACAGCCTATTTTGCTAGTTTGTTTTGT
TTCGTTTTGTTTTGATGGAGAGCGTATGTTAGTACTATCGATTCACACAAAAAACCAACACACAGATGTAATGAAAATAA
AGATATTTTATTGCGGCTGCATCTCAATTAGTCAGCAACCATAGTCCCGCCCCTAACTCCGCCCATCCCGCCCCTAACT
CCGCCCAGTTCCGCCCATTCTCCGCCCCATGGCTGACTAATTTTTTTTATTTATGCAGAGGCCGAGGCCGCCTCGGC
CTCTGAGCTATTCCAGAAGTAGTGAGGAGGCTTTTTTGGAGGCCTAGGCTTTTGCAAAAAGCTTTGCAAAGATGGATA
AAGTTTTAAACAGAGAGGAATCTTTGCAGCTAATGGACCTTCTAGGTCTTGAAAGGAGTGCGTGAG

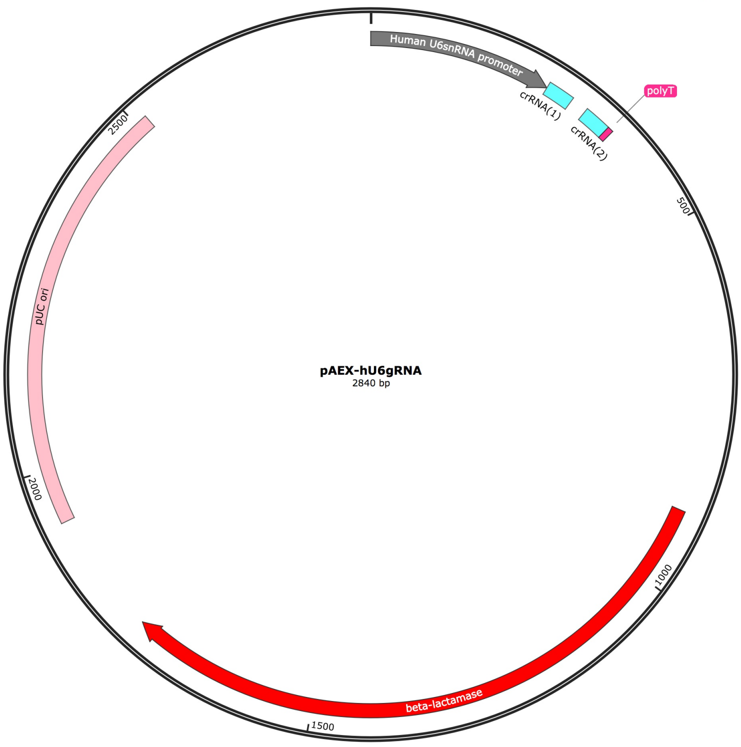

Supplementary Fig. 15. Plasmid vector pAEX-hU6gRNA.

1..249: Human U6 snRNA promoter
250..286: crRNA(1)
310..346: crRNA(2)
347..354: polyT
897..1757: beta-lactamase
1928..2516: pUC ori

>pAEX-hU6gRNA
GAGGGCCTATTTCCCATGATTCCTTCATATTTGCATATACGATACAAGGCTGTTAGAGAGATAATTGGAATTAATTTGACT
GTAAACACAAAGATATTAGTACAAAATACGTGACGTAGAAAGTAATAATTTCTTGGGTAGTTTGCAGTTTTAAAATTATGT
TTTAAAATGGACTATCATATGCTTACCGTAACTTGAAAGTATTTCGATTTCTTGGCTTTATATATCTTGTGGAAAGGACGA
AACACCGTTCCAATTAATCTTAAGCCCTATTAGGGATTGAAACGGAGACCCTCAATTGTCGGTCTCGTTCCAATTAATC
TTAAGCCCTATTAGGGATTGAAACTTTTTTTTGCAAAATTTTCCAGATCGATTTCTTCTTCCTCTGTTCTTCGGCGTTCA
ATTTCTGGGGTTTTCTCTTCGTTTTCTGTAACTGAAACCTAAAATTTGAATGGCGCGCCAAGCTTGCCAACATGAATTG
GTGCACTCTCAGTACAATCTGCTCTGATGCCGCATAGTTAAGCCAGCCCCGACACCCGCCAACACCCGCTGACGCG
CCCTGACGGGCTTGTCTGCTCCCGGCATCCGCTTACAGACAAGCTGTGACCGTCTCCGGAAAATCAAAGGATCTTCT
TGAGATCCTTTTTTTGAGCTGCATGTGTCAGAGGTTTTCACCGTCATCACCGAAACGCGCGAGACGAAAGGGCCTCG
TGATACGCCTATTTTTATAGGTTAATGTCATGATAATAATGGTTTCTTAGACGTCAGGTGGCACTTTTCGGGGAAATGTG
CGCGGAACCCCTATTTGTTTATTTTTCTAAATACATTCAAATATGTATCCGCTCATGAGACAATAACCCTGATAAATGCTT
CAATAATATTGAAAAAGGAAGAGTATGAGTATTCAACATTTCCGTGTCGCCCTTATTCCCTTTTTTGCGGCATTTTGCCT
TCCTGTTTTTGCTCACCCAGAAACGCTGGTGAAAGTAAAAGATGCTGAAGATCAGTTGGGTGCACGAGTGGGTTACA
TCGAACTGGATCTCAACAGCGGTAAGATCCTTGAGAGTTTTCGCCCCGAAGAACGTTTTCCAATGATGAGCACTTTTA
AAGTTCTGCTATGTGGCGCGGTATTATCCCGTATTGACGCCGGGCAAGAGCAACTCGGTCGCCGCATACACTATTCTC
AGAATGACTTGGTTGAGTACTCACCAGTCACAGAAAAGCATCTTACGGATGGCATGACAGTAAGAGAATTATGCAGTG
CTGCCATAACCATGAGTGATAACACTGCGGCCAACTTACTTCTGACAACGATCGGAGGACCGAAGGAGCTAACCGCT
TTTTTGCACAACATGGGGGATCATGTAACTCGCCTTGATCGTTGGGAACCGGAGCTGAATGAAGCCATACCAAACGA
CGAGCGTGACACCACGATGCCTGTAGCAATGGCAACAACGTTGCGCAAACTATTAACTGGCGAACTACTTACTCTAG
CTTCCCGGCAACAATTAATAGACTGGATGGAGGCGGATAAAGTTGCAGGACCACTTCTGCGCTCGGCCCTTCCGGCT
GGCTGGTTTATTGCTGATAAATCTGGAGCCGGTGAGCGTGGGAGTCGCGGTATCATTGCAGCACTGGGGCCAGATG
GTAAGCCCTCCCGTATCGTAGTTATCTACACGACGGGGAGTCAGGCAACTATGGATGAACGAAATAGACAGATCGCTG
AGATAGGTGCCTCACTGATTAAGCATTGGTAACTGTCAGACCAAGTTTACTCATATATACTTTAGATTGATTTAAAACTTC
ATTTTTAATTTAAAAGGATCTAGGTGAAGATCCTTTTTGATAATCTCATGACCAAAATCCCTTAACGTGAGTTTTCGTTCC
ACTGAGCGTCAGACCCCGTAGAAAAGATCAAAGGATCTTCTTGAGATCCTTTTTTTCTGCGCGTAATCTGCTGCTTGC
AAACAAAAAAACCACCGCTACCAGCGGTGGTTTGTTTGCCGGATCAAGAGCTACCAACTCTTTTTCCGAAGGTAACT

GGCTTCAGCAGAGCGCAGATACCAAATACTGTTCTTCTAGTGTAGCCGTAGTTAGGCCACCACTTCAAGAACTCTGTA
GCACCGCCTACATACCTCGCTCTGCTAATCCTGTTACCAGTGGCTGCTGCCAGTGGCGATAAGTCGTGTCTTACCGG
GTTGGACTCAAGACGATAGTTACCGGATAAGGCGCAGCGGTCGGGCTGAACGGGGGGTTCGTGCACACAGCCCAG
CTTGGAGCGAACGACCTACACCGAACTGAGATACCTACAGCGTGAGCTATGAGAAAGCGCCACGCTTCCCGAAGGG
AGAAAGGCGGACAGGTATCCGGTAAGCGGCAGGGTCGGAACAGGAGAGCGCACGAGGGAGCTTCCAGGGGGAAA
CGCCTGGTATCTTTATAGTCCTGTCGGGTTTCGCCACCTCTGACTTGAGCGTCGATTTTTGTGATGCTCGTCAGGGG
GGCGGAGCCTATGGAAAAACGCCAGCAACGCGGCCTTTTTACGGTTCCTGGCCTTTTGCTGGCCTTTTGCTCACATG
TTCTTTCCTGCGTTATCCCCTGATTCTGTGGATAACCGTATTACCGCCTTTGAGTGAGCTGATACCGCTCGCCGCAGC
CGAACGACCGAGCGCAGCGAGTCAGTGAGCGAGGAAGCGGAAGAGCAAATAATAAAAAAGCCGGATTAATAATCTG
GCTTTTTATATTCTCTGCCCAATACGCAAACCGCCTCTCCCCGCGCGTTGGCCGATTCATTAATGCAGCTGGCACGAC
AGGCCAGTGCCAAGCTCCAATTAGGGCCCCAA

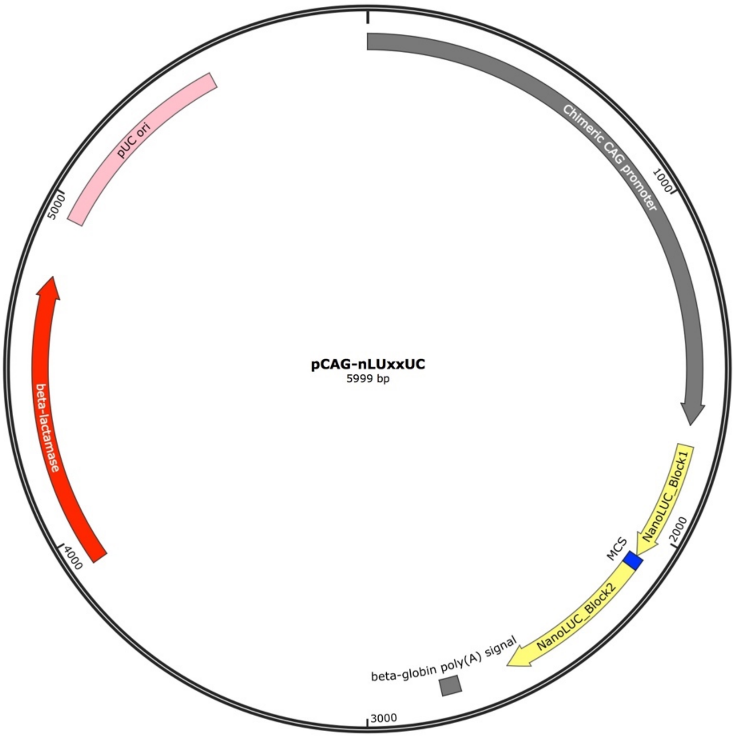

Supplementary Fig. 16. Plasmid vector pCAG-nLUxxUC.

1..1665: Chimeric CAG promoter
1726..2079: NanoLUC_Block1 (#1 to #351 of NanoLUC with synthetic stop codon)
2080..2115: MCS
2116..2580: NanoLUC_Block2 (#52 to the original stop codon of NanoLUC)
2729..2784: rabbit beta-globin poly(A) signal
3912..4772: beta lactamase
4943..5531: pUC ori

>pCAG-nLUxxUC
GACATTGATTATTGACTAGTTATTAATAGTAATCAATTACGGGGTCATTAGTTCATAGCCCATATATGGAGTTCCGCGTTA
CATAACTTACGGTAAATGGCCCGCCTGGCTGACCGCCCAACGACCCCCGCCCATTGACGTCAATAATGACGTATGTT
CCCATAGTAACGCCAATAGGGACTTTCCATTGACGTCAATGGGTGGACTATTTACGGTAAACTGCCCACTTGGCAGTA
CATCAAGTGTATCATATGCCAAGTACGCCCCCTATTGACGTCAATGACGGTAAATGGCCCGCCTGGCATTATGCCCAG
TACATGACCTTATGGGACTTTCCTACTTGGCAGTACATCTACGTATTAGTCATCGCTATTACCATGGTCGAGGTGAGC
CCCACGTTCTGCTTCACTCTCCCCATCTCCCCCCCCTCCCCACCCCCAATTTTGTATTTATTTATTTTTTAATTATTTTGT
GCAGCGATGGGGGCGGGGGGGGGGGGGGCGCGCGCCAGGCGGGGCGGGGCGGGGCGAGGGGCGGGGCGGG
GCGAGGCGGAGAGGTGCGGCGGCAGCCAATCAGAGCGGCGCGCTCCGAAAGTTTCCTTTTATGGCGAGGCGGCG
GCGGCGGCGGCCCTATAAAAAGCGAAGCGCGCGGCGGGCGGGAGTCGCTGCGTTGCCTTCGCCCCGTGCCCCGC
TCCGCGCCGCCTCGCGCCGCCCGCCCCGGCTCTGACTGACCGCGTTACTCCCACAGGTGAGCGGGCGGGACGGC
CCTTCTCCTCCGGGCTGTAATTAGCGCTTGGTTTAATGACGGCTCGTTTCTTTTCTGTGGCTGCGTGAAAGCCTTAAA
GGGCTCCGGGAGGGCCCTTTGTGCGGGGGGGAGCGGCTCGGGGGGTGCGTGCGTGTGTGTGTGCGTGGGGAGC
GCCGCGTGCGGCCCGCGCTGCCCGGCGGCTGTGAGCGCTGCGGGCGCGGCGCGGGGCTTTGTGCGCTCCGCGT
GTGCGCGAGGGGAGCGCGGCCGGGGGCGGTGCCCCGCGGTGCGGGGGGGCTGCGAGGGGAACAAAGGCTGCG
TGCGGGGTGTGTGCGTGGGGGGGTGAGCAGGGGGTGTGGGCGCGGCGGTCGGGCTGTAACCCCCCCCTGCACC
CCCCTCCCCGAGTTGCTGAGCACGGCCCGGCTTCGGGTGCGGGGCTCCGTGCGGGGCGTGGCGCGGGGCTCGC
CGTGCCGGGCGGGGGGTGGCGGCAGGTGGGGGTGCCGGGCGGGGCGGGGCCGCCTCGGGCCGGGGAGGGCT
CGGGGGAGGGGCGCGGCGGCCCCGGAGCGCCGGCGGCTGTCGAGGCGCGGCGAGCCGCAGCCATTGCCTTTTA
TGGTAATCGTGCGAGAGGGCGCAGGGACTTCCTTTGTCCCAAATCTGGCGGAGCCGAAATCTGGGAGGCGCCGCC
GCACCCCCTCTAGCGGGCGCGGGCGAAGCGGTGCGGCGCCGGCAGGAAGGAAATGGGCGGGGAGGGCCTTCGT
GCGTCGCCGCGCCGCCGTCCCCTTCTCCATCTCCAGCCTCGGGGCTGCCGCAGGGGGACGGCTGCCTTCGGGGG
GGACGGGGCAGGGCGGGGTTCGGCTTCTGGCGTGTGACCGGCGGCTCTAGCCTCTGCTAACCATGTTCATGCCTT
CTTCTTTTTCCTACAGCTCCTGGGCAACGTGCTGGTTATTGTGCTGTCTCATCATTTTGGCAAATCTAGAGCCGCCAT
GGTCTTCACACTCGAAGATTTCGTTGGGGACTGGCGACAGACAGCCGGCTACAACCTGGACCAAGTCCTTGAACAG

GGAGGTGTGTCCAGTTTGTTTCAGAATCTCGGGGTGTCCGTAACTCCGATCCAAAGGATTGTCCTGAGCGGTGAAAA
TGGGCTGAAGATCGACATCCATGTCATCATCCCGTATGAAGGTCTGAGCGGCGACCAAATGGGCCAGATCGAAAAAA
TTTTTAAGGTGGTGTACCCTGTGGATGATCATCACTTTAAGGTGATCCTGCACTATGGCACACTGGTAATCGACGGGG
TTACGCCGAACATGATCGACTATTTCGGACGGCCGTATGAATGAGGATCCGCTAGCCTGCAGGTCGACGAATTCGATA
TCTACAACCTGGACCAAGTCCTTGAACAGGGAGGTGTGTCCAGTTTGTTTCAGAATCTCGGGGTGTCCGTAACTCCG
ATCCAAAGGATTGTCCTGAGCGGTGAAAATGGGCTGAAGATCGACATCCATGTCATCATCCCGTATGAAGGTCTGAG
CGGCGACCAAATGGGCCAGATCGAAAAAATTTTTAAGGTGGTGTACCCTGTGGATGATCATCACTTTAAGGTGATCCT
GCACTATGGCACACTGGTAATCGACGGGGTTACGCCGAACATGATCGACTATTTCGGACGGCCGTATGAAGGCATCG
CCGTGTTCGACGGCAAAAAGATCACTGTAACAGGGACCCTGTGGAACGGCAACAAAATTATCGACGAGCGCCTGATC
AACCCCGACGGCTCCCTGCTGTTCCGAGTAACCATCAACGGAGTGACCGGCTGGCGGCTGTGCGAACGCATTCTG
GCGTAACTCGAGACTCCTCAGGTGCAGGCTGCCTATCAGAAGGTGGTGGCTGGTGTGGCCAATGCCCTGGCTCACA
AATACCACTGAGATCTTTTTCCCTCTGCCAAAAATTATGGGGACATCATGAAGCCCCTTGAGCATCTGACTTCTGGCTA
ATAAAGGAAATTTATTTTCATTGCAATAGTGTGTTGGAATTTTTTGTGTCTCTCACTCGGAAGGACATATGGGAGGGCA
AATCATTTAAAACATCAGAATGAGTATTTGGTTTAGAGTTTGGCAACATATGCCCATATGCTGGCTGCCATGAACAAAG
GTTGGCTATAAAGAGGTCATCAGTATATGAAACAGCCCCCTGCTGTCCATTCCTTATTCCATAGAAAAGCCTTGACTTG
AGGTTAGATTTTTTTTATATTTTGTTTTGTGTTATTTTTTTCTTTAACATCCCTAAAATTTTCCTTACATGTTTTACTAGCCA
GATTTTTCCTCCTCTCCTGACTACTCCCAGTCATAGCTGTCCCTCTTCTCTTATGAAGATCCCTCGACTTAATTAAGGTA
CCCAATTCGCCCTATAGTGAGTCGTATTACGCGCGCTCACTGGCCGTCGTTTTACAACGTCGTGACTGGGAAAACCC
TGGCGTTACCCAACTTAATCGCCTTGCAGCACATCCCCCTTTCGCCAGCTGGCGTAATAGCGAAGAGGCCCGCACC
GATCGCCCTTCCCAACAGTTGCGCAGCCTGAATGGCGAATGGGACGCGCCCTGTAGCGGCGCATTAAGCGCGGCG
GGTGTGGTGGTTACGCGCAGCGTGACCGCTACACTTGCCAGCGCCCTAGCGCCCGCTCCTTTCGCTTTCTTCCCTT
CCTTTCTCGCCACGTTCGCCGGCTTTCCCCGTCAAGCTCTAAATCGGGGGCTCCCTTTAGGGTTCCGATTTAGTGCT
TTACGGCACCTCGACCCCAAAAAACTTGATTAGGGTGATGGTTCACGTAGTGGGCCATCGCCCTGATAGACGGTTTT
TCGCCCTTTGACGTTGGAGTCCACGTTCTTTAATAGTGGACTCTTGTTCCAAACTGGAACAACACTCAACCCTATCTC
GGTCTATTCTTTTGATTTATAAGGGATTTTGCCGATTTCGGCCTATTGGTTAAAAAATGAGCTGATTTAACAAAAATTTAA
CGCGAATTTTAACAAAATATTAACGCTTACAATTTAGGTGGCACTTTTCGGGGAAATGTGCGCGGAACCCCTATTTGTT
TATTTTTCTAAATACATTCAAATATGTATCCGCTCATGAGACAATAACCCTGATAAATGCTTCAATAATATTGAAAAAGGAA
GAGTATGAGTATTCAACATTTCCGTGTCGCCCTTATTCCCTTTTTTGCGGCATTTTGCCTTCCTGTTTTTGCTCACCCA
GAAACGCTGGTGAAAGTAAAAGATGCTGAAGATCAGTTGGGTGCACGAGTGGGTTACATCGAACTGGATCTCAACAG
CGGTAAGATCCTTGAGAGTTTTCGCCCCGAAGAACGTTTTCCAATGATGAGCACTTTTAAAGTTCTGCTATGTGGCGC
GGTATTATCCCGTATTGACGCCGGGCAAGAGCAACTCGGTCGCCGCATACACTATTCTCAGAATGACTTGGTTGAGTA
CTCACCAGTCACAGAAAAGCATCTTACGGATGGCATGACAGTAAGAGAATTATGCAGTGCTGCCATAACCATGAGTGA
TAACACTGCGGCCAACTTACTTCTGACAACGATCGGAGGACCGAAGGAGCTAACCGCTTTTTTGCACAACATGGGGG
ATCATGTAACTCGCCTTGATCGTTGGGAACCGGAGCTGAATGAAGCCATACCAAACGACGAGCGTGACACCACGATG
CCTGTAGCAATGGCAACAACGTTGCGCAAACTATTAACTGGCGAACTACTTACTCTAGCTTCCCGGCAACAATTAATAG
ACTGGATGGAGGCGGATAAAGTTGCAGGACCACTTCTGCGCTCGGCCCTTCCGGCTGGCTGGTTTATTGCTGATAAA
TCTGGAGCCGGTGAGCGTGGGTCTCGCGGTATCATTGCAGCACTGGGGCCAGATGGTAAGCCCTCCCGTATCGTAG
TTATCTACACGACGGGGAGTCAGGCAACTATGGATGAACGAAATAGACAGATCGCTGAGATAGGTGCCTCACTGATTA
AGCATTGGTAACTGTCAGACCAAGTTTACTCATATATACTTTAGATTGATTTAAAACTTCATTTTTAATTTAAAAGGATCTA
GGTGAAGATCCTTTTTGATAATCTCATGACCAAAATCCCTTAACGTGAGTTTTCGTTCCACTGAGCGTCAGACCCCGT
AGAAAAGATCAAAGGATCTTCTTGAGATCCTTTTTTTCTGCGCGTAATCTGCTGCTTGCAAACAAAAAAACCACCGCTA
CCAGCGGTGGTTTGTTTGCCGGATCAAGAGCTACCAACTCTTTTTCCGAAGGTAACTGGCTTCAGCAGAGCGCAGAT
ACCAAATACTGTCCTTCTAGTGTAGCCGTAGTTAGGCCACCACTTCAAGAACTCTGTAGCACCGCCTACATACCTCGC
TCTGCTAATCCTGTTACCAGTGGCTGCTGCCAGTGGCGATAAGTCGTGTCTTACCGGGTTGGACTCAAGACGATAGT
TACCGGATAAGGCGCAGCGGTCGGGCTGAACGGGGGGTTCGTGCACACAGCCCAGCTTGGAGCGAACGACCTACA
CCGAACTGAGATACCTACAGCGTGAGCTATGAGAAAGCGCCACGCTTCCCGAAGGGAGAAAGGCGGACAGGTATCC
GGTAAGCGGCAGGGTCGGAACAGGAGAGCGCACGAGGGAGCTTCCAGGGGGAAACGCCTGGTATCTTTATAGTCC
TGTCGGGTTTCGCCACCTCTGACTTGAGCGTCGATTTTTGTGATGCTCGTCAGGGGGGCGGAGCCTATGGAAAAAC
GCCAGCAACGCGGCCTTTTTACGGTTCCTGGCCTTTTGCTGGCCTTTTGCTCACATGTTCTTTCCTGCGTTATCCCCT
GATTCTGTGGATAACCGTATTACCGCCTTTGAGTGAGCTGATACCGCTCGCCGCAGCCGAACGACCGAGCGCAGCG
AGTCAGTGAGCGAGGAAGCGGAAGAGCGCCCAATACGCAAACCGCCTCTCCCCGCGCGTTGGCCGATTCATTAATG
CAGCTGGCACGACAGGTTTCCCGACTGGAAAGCGGGCAGTGAGCGCAACGCAATTAATGTGAGTTAGCTCACTCAT
TAGGCACCCCAGGCTTTACACTTTATGCTTCCGGCTCGTATGTTGTGTGGAATTGTGAGCGGATAACAATTTCACACA
GGAAACAGCTATGACCATGATTACGCCAAGCGCGCAATTAACCCTCACTAAAGGGAACAAAAGCTGGAGCTCATCGA
TCTC

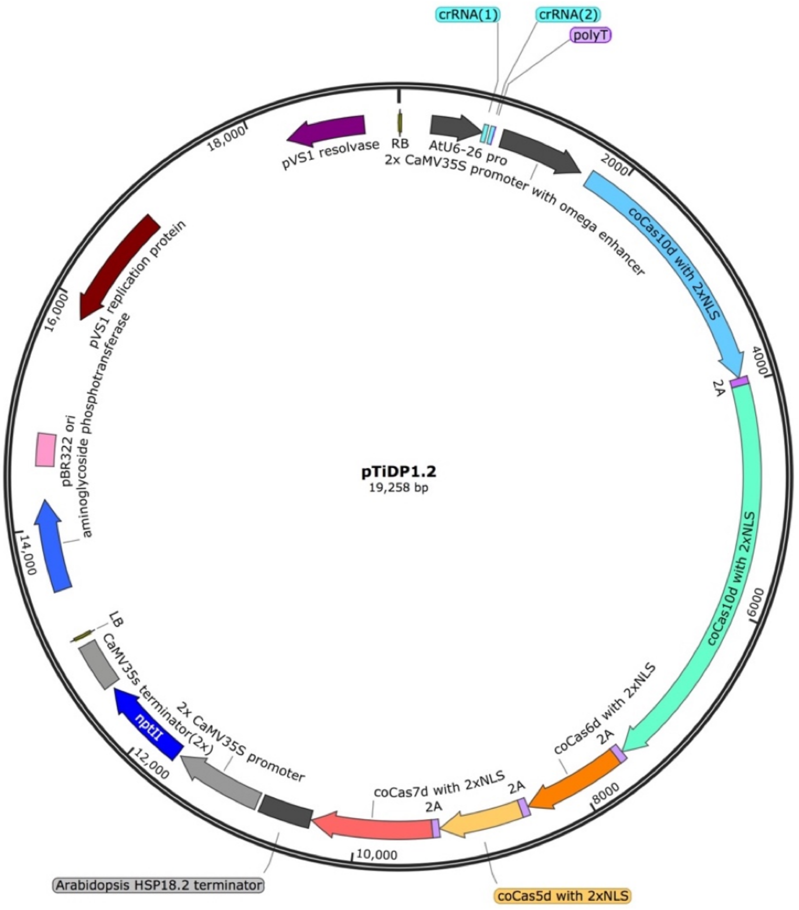

Supplementary Fig. 17. Plasmid vector pTiDP1.2.

1..26: RB, T-DNA right border
287..734: Arabidopsis U6 snRNA-26 promoter
735..771: crRNA(1)
795..831: crRNA(2)
832..839: polyT
908..1656: 2x CaMV35S promoter with omega translational enhancer
1737..3953: coCas10d with 2xNLS
3954..4019: 2A self-cleavage peptide
4020..7538: coCas10d with 2xNLS
7539..7604: 2A self-cleavage peptide
7605..8486: coCas6 with 2xNLS-
8487..8552: 2A self-cleavage peptide
8553..9275: coCas5d with 2xNLS
9276..9338: 2A self-cleavage peptide
9339..10391: coCas7d with 2xNLS
10398..10844: Arabidopsis HSP18.2 terminator
10880..11659: 2x CaMV35S promoter
11690..12484: nptII
12522..12933: CaMV35s terminator (2x)
13000..13025: LB, T-DNA Left border
13450..14244: Aminoglycoside phosphotransferase
14535..14815: pBR322 ori
15849..16921 (complement): pVS1 replication protein
18278..18964 (complement): pVS1 resolvase

>pTiDP1.2
TAAACGCTCTTTTCTCTTAGGTTTACCCGCCAATATATCCTGTCAAACACTGATAGTTTAAACTGAAGGCGGGAAACGA
CAATCTGATCCAAGCTCAAGCTGCTCTAGCATTCGCCATTCAGGCTGCGCAACTGTTGGGAAGGGCGATCGGTGCG

GGCCTCTTCGCTATTACGCCAGCTGGCGAAAGGGGGATGTGCTGCAAGGCGATTAAGTTGGGTAACGCCAGGGTTT
TCCCAGTCACGACGTTGTAAAACGACGGCCAGTGCCAAGCTCCAATTAGGGCCCCAAGCTTCGTTGAACAACGGAA
ACTCGACTTGCCTTCCGCACAATACATCATTTCTTCTTAGCTTTTTTTCTTCTTCTTCGTTCATACAGTTTTTTTTTGTTT
ATCAGCTTACATTTTCTTGAACCGTAGCTTTCGTTTTCTTCTTTTTAACTTTCCATTCGGAGTTTTTGTATCTTGTTTCAT
AGTTTGTCCCAGGATTAGAATGATTAGGCATCGAACCTTCAAGAATTTGATTGAATAAAACATCTTCATTCTTAAGATAT
GAAGATAATCTTCAAAAGGCCCCTGGGAATCTGAAAGAAGAGAAGCAGGCCCATTTATATGGGAAAGAACAATAGTAT
TTCTTATATAGGCCCATTTAAGTTGAAAACAATCTTCAAAAGTCCCACATCGCTTAGATAAGAAAACGAAGCTGAGTTTA
TATACAGCTAGAGTCGAAGTAGTGATTGTTCCAATTAATCTTAAGCCCTATTAGGGATTGAAACGGAGACCCTCAATTG
TCGGTCTCGTTCCAATTAATCTTAAGCCCTATTAGGGATTGAAACTTTTTTTTGCAAAATTTTCCAGATCGATTTCTTCTT
CCTCTGTTCTTCGGCGTTCAATTTCTGGCGCGCCAAGCTTGCCAACATGGTGGAGCACGACACTCTCGTCTACTCCA
AGAATATCAAAGATACAGTCTCAGAAGACCAAAGGGCTATTGAGACTTTTCAACAAAGGGTAATATCGGGAAACCTCC
TCGGATTCCATTGCCCAGCTATCTGTCACTTCATCAAAAGGACAGTAGAAAAGGAAGGTGGCACCTACAAATGCCATC
ATTGCGATAAAGGAAAGGCTATCGTTCAAGATGCCTCTGCCGACAGTGGTCCCAAAGATGGACCCCCACCCACGAG
GAGCATCGTGGAAAAAGAAGACGTTCCAACCACGTCTTCAAAGCAAGTGGATTGATGTGAACATGGTGGAGCACGA
CACTCTCGTCTACTCCAAGAATATCAAAGATACAGTCTCAGAAGACCAAAGGGCTATTGAGACTTTTCAACAAAGGGT
AATATCGGGAAACCTCCTCGGATTCCATTGCCCAGCTATCTGTCACTTCATCAAAAGGACAGTAGAAAAGGAAGGTGG
CACCTACAAATGCCATCATTGCGATAAAGGAAAGGCTATCGTTCAAGATGCCTCTGCCGACAGTGGTCCCAAAGATGG
ACCCCCACCCACGAGGAGCATCGTGGAAAAAGAAGACGTTCCAACCACGTCTTCAAAGCAAGTGGATTGATGTGATA
TCTCCACTGACGTAAGGGATGACGCACAATCCCACTATCCTTCGCAAGACCCTTCCTCTATATAAGGAAGTTCATTTCA
TTTGGAGAGGCCGGTCTAGAGTATTTTTACAACAATTACCAACAACAACAAACAACAAACAACATTACAATTACTATTTA
CAATTACAAGCACCATGGACCCAAAGAAGAAGCGGAAGGTAGACCCTAAGAAGAAGCGCAAGGTTTCTGGAGGAAA
CTATCAGGTGACTCTTAAGCCAGTGTATTCTTGCCCAGCTGACGAGATCCCAGACGGAATCAAGGTGCCACAGGGAT
GGAGACTTTCTTGGCACCAGGTGGAGACTTGGAAGGCTCTTAACGACCCAGACATCGACGTGATCTTCAACACTGCT
ATGACTGGAGACGGAAAGTCTCTTGCTGCTTATCTTAGAACTCTTCAGGGATATTTCCCAATCATGGGACTTTATCCAA
CTAACGAGCTTGCTAGAGATCAGAGAGGACAGATCGAGGCTTATATCCAGAGATTCCAGCCAACTGACCAGCCAAGA
GTGAACCTTCTTACTGGACCAGAGCTTGAGCTTTATGCTGAGAGAGACGGAAAGACTAAGGCTATCGCTCTTGAGAC
TAGATCTAAGCAGTCTGAGATCCTTCTTACTAACCCAGACATCTTCCACTATCTTCACAGAGCTGCTTATCTTACTCCAT
ATGACAACCCAGACCAGCTTTGGAACAGAATCGACAAGCACTTCGACCTTTTCCTTTTCGACGAGTTCCACGTGTTC
GGAACTCCACAGGTGGCTTCTATCATCAACACTATGCTTCTTATCAGAAGAGCTAACAGAGGAAAGAGATATCTTTTCC
TTTCTGCTACTCCAGACGAGGGACTTCTTAAGAGACTTGACAAGGCTGGATTCAGATATAGATCTATCGACCCAGTGA
GAGAGGGAAAGTATAGATTCCCAGACACTCCAGAGGAGGCTAACTCTCTTGCTCAGCAGGGATGGAGACAGGTGAC
TTCTGAGATCGAGCTTTCTTTCATCCCACTTCCATCTTCTTTCCAGACTTCTGAGAACTGGCTTAAGGAGAACAAGGA
GAGAATCCTTGACTATTTCAAGAGATATCCAGGATCTAAGGGAGCTATCATCCTTAACTCTATCGCTTCTGTGAAGAGA
CTTCTTCCAATCTTCAGAGAGCTTCTTGCTACTATCGGACTTACTGTGGGAGAGAACACTGGACTTTCTGGAACTAGA
GAGAAACTTGCTTCTCTTAACAGAGATCTTGTGATCGGAACTTCTACTATCGACGTGGGAGTGGACTTCAAGATCAAC
TTCCTTATCTTCGAGTCTTCTGACGCTGGAAACTTCATCCAGAGATTCGGAAGACTTGGAAGACACTCTGGATATGAC
AGAAAGGGAACTGCTGTGAAGTTCACTAACTTCACTGCTATCGCTCTTGTGCCAAAGTTCTTCCTTGAGAGACTTTTC
GAGAAGAAGGACGCTCCACTTCAGGTGGGAGAGAGATATGACAGAATCCAGCTTCAGGAGGCTATCAAGTCTAACTA
TAGACACATCAACAACTTCGAGGGATATTATCAGAGATGGGGAGCTGTGCAGTCTTTCCAGCTTTGGTGGAACCTTG
GATCTCCAAAGATCAAGTCTCAGTATGGAGAGTCAAGACAGAAGTTCCAGCAGGAGTGCGAGGAGGTGTTCGACAC
TTCTCTTAAGAGAGTGGCTGGAAGAGTGAAGGGATGGGCTGACGAGTGGAAGGAGCTTTCTGGAAAGAACGGAAAC
CCAATCTTCGAGGACGCTTCTTCTTTCAGAGGATCTTCTCCACTTCTTTGCGGACTTTATGACTCTACTGAGCCAGAG
GAGTGCGACAGATTCAAGACTTATGACCTTCCATCTATCCTTGGAAACCTTGAGGTGGAGGTGTGGAGAAAGGGAGA
GTTCAAGAGACAGATCGAGGCTACTAAGACTCCAATCGCTAGAAGAAGATTCGACTATTGCCTTGCTTTCCTTAACCTT
AAGGGATATAGAGAGGAGAGACTTAACTGGAGATTCACTTATGACGGAGATCTTGGAGAGATCGCTTCTGCTTGGAA
GGTGCAGGTGCTTACTGGAATCGGAGTGTGGCAGCCAGACAACCCTTGGCTTGACAGAATCTCAAGAGAGCTTAGA
GATCTTGCTCTTGTGTCTTTCGTGTTCGCTTATCCAGTGGCTGCTGTGAGACAGAGACTTCAGCTTCCAATGCACTTC
GGAATCTATCCAATCTCTGACGAGTCTTCTCTTCACTCTCCACTTTCTCCATATTCTATCGCTATCGGACAGGCTGCTC
TTCTTCTTGACACTCTTGCTCACAGATTCAAGGGAAAGGGAGGAGAGGTGTGGATCTGCGGCTCTGAGGGCAGAGG
CAGCCTGCTGACCTGCGGCGACGTGGAGGAAAACCCTGGCCCTGAATTCGACCCAAAGAAGAAGCGGAAGGTAGA
CCCTAAGAAGAAGCGCAAGGTTTCTGGACCAAAGAAGCAGAAGAAGCTTGAGGAGACTGGACAGCTTAACCTTTTC
GACAACACTACTGAGATCGACGACGAGGACCTTGACTTCGAGTTCGAGGACATCGACCTTGAGTCTCTTGTGTCTGA
GGACCTTGGAATCACTGAGTCTGTGTCTGACAGAAGAGTGGAGACTGTGAGACAGCTTCTTACTCTTAAGCTTCTTA
GAGAGGCTATCAGAGCTGAGAACCCAGACGACAGAGTGATGGCTGACTTCGCTGAGATGGTGCTTCCAAACCTTCT
TAGACTTGCTATCGGAGTGACTGCTAAGGGAGGAAACTTCATCGAGGCTGTGGACAGAGGAAGAGAGCTTAGAAACA
AGCCAAAGGCTAAGAGAGACAACGCTGGAGATCAGTCTCTTAACACTCACCTTCTTAACGGACTTTTCCCAGCTAAC
CTTATCGAGAAGAGACTTCAGAAGCTTAACACTACTGTGAGAAGAATCATCAAGGAGTTCGAGAGAAGACTTGCTATC
GCTGGATTCCTTGTGCACGACTTCGAGAAGTTCTCTTATGACAGATTCCCATCTATGTCTGAGAGATATATCCAGATCC
AGAGAGACTTCATCCAGGACCCATTCAAGAACCAGGACCCAAGAAAGCTTTCAAGAGAGGAGCACAGAGAGATCCT
TCAGGTGCTTATCCCAGAGCTTGGACTTGACAGATTCCTTTTCCCAGACAACCCAGAGAGATGGCTTGAGTATCTTGA

CGACCTTCTTTATATCGCTAAGAACACTCAGAGAAGAAACGACACTGACCTTAACACTTCTGAGGACGGACTTAACGT
GAGACTTAACGACAGAGTGATCGAGTCTCTTTGCGACCTTGCTTGCCTTGCTGACAGACTTGCTTCTATCATCAAGCA
CCCACACGACGCTGAGAAGGCTTCTCTTCAGGACCTTCTTTATTCTCTTTCTGACGGAGAGCTTAAGTTCACTTATCA
CTCTATCGCTGAGAACAGAGGAGTGCTTACTAACGTGCTTAACAACGCTGTGATGGAGGCTCACCAGGAGCTTGACT
ATCAGCCACTTCTTTATCTTCCAACTGGAGTGGTGTATATCGCTCCAAAGAACGCTCCAGAGGTGTCTCTTGAGACTC
TTCCAAACAGAGTGGTGGACACTATCAAGTCTCTTTGCTCTGGAGAGCTTCAGAGAAAGCAGACTGGATTCGGAAGA
GACGGAAAGGGtATGAAGTATGCTGACTATTATTCTCAGTTCTTCGACGACGCTGGACTTATGAGAGCTGCTCTTAACG
CTACTCTTAGAATCCTTGGAGACAACAAGGCTTCTGTGGCTAGATCTAGAGGAGAGAACCTTATCAAGTTCCAGCAGC
AGGGAGTGCTTCCAACTGACTATGACTTCCACTGCGAGGACGACATCAGAATCGACAGACTTGCTGAGTTCGGAGA
CGTGGTGACTAGAAAGATCTGGGGAGACAGACTTGAGAAGATCGAGCAGGCTAGAAAGCTTCAGAAGAACCTTCCA
GCTCCACCAGACCTTGACCTTATCTCTGAGATCGCTCACTATTGGAACCTTGAGAACTATCTTCCACAGATCAGAGCT
ATCAAGAGAATCAACGAGTCTCTTAAGGAGCTTAAGCTTAAGGGAAACACTGGAGGAGTGCCATATGAGTGGTATTAT
CTTGCTGCTCAGTATCTTAAGCAGCACCCAGGAATCGAGGACATCAGACCAGTGGCTGAGGACCTTATCGCTTTCCT
TGCTGCTAAGATCGCTGCTATCGTGGCTGGATATAACCTTCCAGACGGATGGGAGGACCTTAGAGAGTGGGTGAACC
AGGTGGTGCAGCTTCCAGGAAGAGAGCTTGCTCACTCTATCGAGACTTTCCAGAAGGAGCTTAACCACTATAACGCT
GCTAAGAAGCAGGGAAGAGGAAGACAGCTTCTTTGCTCTATCTCTCACTCTCCATATTCTGTGTCTGAGCAGATGGAG
TCTGCTGTGCTTTTCACTCCACAGGTGTATACTAACAAGCAGATGCTTGCTGGATCTAACGCTAAGAGAAACATCTCTT
CTATCGCTGGAACTGAGATGATGCTTAGACAGATCCTTATGAACCAGACTCAGGCTGTGGGAAAGAGATTCGAGGAC
GGAAAGTATAGATATCTTTATTTCTATCCAACTTATTATTTCACTCCAGAGACTAACTCTTTCCTTCAGAAGGCTTATGCT
AACATCGCTCAGACTAGATTCGACTCTTCTATCAAGCTTCACTTCGTGGACAAGAACCTTGTGGCTAACTTCGACAGA
ACTAGATATCAGTCTGTGGACTCTTTCCTTATCGACGAGAAGCTTAGACAGAAGAAGGAGACTATCAACGAGGAGGAG
GACGGAAAGAAGGACAGAACTTTCAAGCTTTCTTATCCAGAGGACAAGCCACTTACTTTCTATTTCATGGCTCTTCCA
CCAGGAAGAAACCCAACTGACACTGAGTCTTGGGTAATGCCAGCTTGGCTTGGACTTGCTTTCCCAATGATCCTTGA
CGTGAAGACTGTGGTGTCTGAGTCTCCAATCCCACCATATAGAGACGGAGCTGAGTTCGAGGAGACTGTGTTCCTTG
ACTCTGCTCCACAGGCTATCAGATCTCTTACTAGATGCGACAGATTCAGACTTGACAGAGTGCTTAACCCTTGGCAGG
ACAACGACGGAAAGAAGTATTCTGCTCCACTTAACACTCTTACTGCTGCTTATTCTATCCACCTTGACGTGAACTCTAA
GCAGGGAAAGACTGGATATGACCCAAACTGGGGAAAGCTTACTGAGCTTGCTATCAACCTTGAGACTTCTCCACTTTA
TGTGTTCCACTATTTCAAGCAGTGGAAGAGAGGAAAGGACGCTGACATCCCATCTGCTAACAGAATCGCTCTTTATCT
TTATGACTTCTATCCATGCTTCGACCCATATGTGCAGGCTAACAGAACTAACCTTACTATCGACATGACTGCTGAGTCT
CCACTTAACCACCCAAAGAACCTTACTGAGCTTTATAGACAGTTCTATAGAGCTAAGTCTTCTAAGGGAAAGCCAATCA
AGGCTAACGCTATCCTTAAGCCAATCGACGAGGCTGCTGACATCATCCTTAAGGCTGACAAGGCTATCTCTGACGACC
TTACTTCTCTTGTGGCTGCTAGACTTTTCAAGCTTATGGACAGAGTGAGATCTCAGACTGCTGAGGGAAGATATGTGA
TCAAGGAGAGAGATCAGGAGAGAGAGAAGATCCTTGACTTCGCTAAGTATTTCGTGAAGAACGTGTTCGAGGAGTCT
TTCGAGTCTGACAGAGCTAGACTTGCTGGAAGACAGCTTAACATCATCAGAGACACTTGCGAGTTCCTTTATAGACTT
GAGATGGACAAGGAGAGAAGACAGAGACAGGTGCAGCCACTTGACACTTCTAACTCTTCTTCTGAGGAGGAGGAGG
GGTCTGAGGGACGCGGCTCCCTGCTCACCTGTGGAGATGTGGAAGAGAACCCAGGCCCCAAGCTTGACCCAAAGA
AGAAGCGGAAGGTAGACCCTAAGAAGAAGCGCAAGGTTTCTGGACCATATTCTCTTGTGCTTAACCTTACTCCAAGAT
CTCCAATCTATCCAAACTTCCTTACTGGAAGACACCTTCACGCTCTTTTCCTTACTCTTGTGTCTTCTGTGGACCAGGA
GCTTGGAAACATCCTTCACACTGCTGAGGCTGACAAGGCTTTCACTCTTTCTCCACTTCAGATGCAGTCTGGAGGAA
AGACTATCAACTCTCCACAGTGGAGACACGAGAGAGAGATCGCTTCTGAGACTCCATGCTGGTGGAGAATCTCTCTT
CTTGACGACAGACTTTTCGGAAAGCTTACTTCTCTTTGGCTTAACCTTAACCCAAAGCAGCCTTGGCACCTTGGATCT
GCTGACCTTGTGATCACTTCTGTGCTTGCTACTCCACAGTCTGTGCAGCCTTGGGCTAACTCTTGCACTTATCAGTAT
CTTTATGAGAACGCTTCTGAGACTAACAGAGAGTTCGACTTCCTTTTCGCTACTCCAGTGACTTTCAGACAGGGAAAG
TTCGACTCTGCTCTTCCAACTAGAGAGCTTGTGTTCAACTCTCTTCTTGGAAGATGGAACAGATATTCTGGAATCCCAT
TCGACTCTATCGCTCTTGAGTCTATCTTCCCATCTTTCTTCGACATCCAGACTAAGCTTGCTGACGAGGCTTATAAGAA
CCAGTCTATCGGATGCGTGGGAGAGATCCACTATAGACTTCTTGGAGAGGTGGAGCCAGCTAAGATCAAGGCTATCA
ACGCTCTTGCTGACTTCGCTCTTTATGCTGGAGTGGGAAGAAAGACTACTATGGGAATGGGAATGACTAGAAGAATCT
CTAAGGACAAGAGAGGTTCTGAAGGCAGAGGCTCTCTGCTGACATGTGGGGATGTGGAGGAAAATCCTGGCCCTAC
GCGTGACCCAAAGAAGAAGCGGAAGGTAGACCCTAAGAAGAAGCGCAAGGTTTCTGGAGTGCACATCTATTCTTGC
CAGCTTGAGCTTCACGACTCTCTTTATTATGCTACTAGAGAGATCGGAAGACTTTATGAGTCTGAGCCAGTGATCCAC
AACTATGCTCTTTGCTATGCTCTTGGACTTGTGAACTCTGACTCTTATAGATATTTCTGCTCTGAGCAGATCCCACAGTA
TCAGGAGCACCTTAACCCACTTAACGAGGAGAAGATCTATGTGACTCCAGCTAGAGCTATCGCTCACACTGCTGTGC
TTAACACTTGGAAGTATGCTAACAACAACTATCACGTGGAGATGGAGAAGACTCAGAAGAACATCCCATCTTTCGGAA
GAGCTAAGGAGATCGCTCCAGAGTCTATCTTCGAGTGCTTCATCATCTCTCACCACCCACTTCAGCTTCCAAAGTGGA
TCAGACTTGGAAAGTGGATGTCTAAGGCTGAGGTGAAGCTTACTGAGCTTTCTCTTTCTAAGCAGAAGGAGGACCTT
TTCATCTATCCATATCCACTTAACCCACTTGACGTGATGTTCACTCACCAGGTTATCGGATATGACGTGATCAACATGCC
ACCAGTGTCTCTTATCAGAAACGTGAGAATGAGAGGAGAGTATTATCAGATCTCTGACAGACCAGACCTTAAGATCCC
AGCTAGACTTTCTTATCACTTCGGAGGATCCGGAGAGGGCAGAGGAAGTCTGCTAACATGCGGTGACGTTGAGGAG
AATCCCGGGCCAGACCCAAAGAAGAAGCGGAAGGTAGACCCTAAGAAGAAGCGCAAGGTTTCTGGAACTTTCCTTA
CTTCTGTGGACGCTAAGTTCTTCCACTCTGAGATCCCATATAAGCCAATGGGAAAGTATGTGCACTTCCTTACTATCAG

AGTGACTGAGTCTTATCCACTTTTCCAGACTGACGGAGAGCTTAACAAGGCTAGAGTGAGAGCTGGAATCGACTCTA
AGAAGACTATCTCAAGACTTTCTATGTTCAAGAGAAAGCAGTCTACTCCAGAGAGACTTGTGGGAAGAGAGCTTCTTA
GAAACTATGGACTTATCACTGCTGAGGAGTGCGAGTATAACGTGAAGTTCGCTATGAACAACGCTGACTGCATCATCT
ATGGATTCGCTATCGGAGACTCTGGATCTGAGAAGTCTAAGGTGGTGGTGGACACTGCTTTCTCTATCACTCCATTCG
ACGAGTCTCACGAGTCTTTCACTCTTAACGCTCCATATGAGAACGGAACTATGGCTTCTAAGGGAGAGAACAACACTA
AGGTGGGAGAGGTGACTTCAAGAATCAACCAGCAGGACCACATCAGACCACAGGTGTTCTTCCCATCTATCGTGACT
CTTAAGGACCCAACTGAGGCTTCTTTCCTTTATGTGTTCAACAACATCCTTAGAACTAGACACTATGGTGCTCAGACTA
CTAGAACTGGAAGAGTGAGAAACGAGCTTATCGGAGTGATCTTCGCTGACGGAGAGATCGTGTCTAACCTTAGATGG
ACTCAGGCTATCTATGACAGACTTCCAGACGAGGTGCTTCACTCTATCGACCCACTTGACGAGGACCTTGTGATGGA
GAAGGCTACTGAGGCTATCCAGGCTCTTATGGCTGAGGAGTTCATCGTGCACACTGACTTCATCGGAGAGAACTTCC
AGCCACTTCTTACTGAGGTGAAGACTCTTACTGGAACTGAGGCTGGAATCCTTTCTGTGCTTGACCAGGCTAACAAG
GAGTCTAAGAAGTATTTCGAGCAGTATATCGAGAAGAAGAAGGCTGAGAAGAAGTAAGAGCTCATATGAAGATGAAGA
TGAAATATTTGGTGTGTCAAATAAAAAGCTTGTGTGCTTAAGTTTGTGTTTTTTTCTTGGCTTGTTGTGTTATGAATTTGT
GGCTTTTTCTAATATTAAATGAATGTAAGATCTCATTATAATGAATAAACAAATGTTTCTATAATCCATTGTGAATGTTTTGT
TGGATCTCTTCTGCAGCATATAACTACTGTATGTGCTATGGTATGGACTATGGAATATGATTAAAGATAAGATGGGCTCA
TAGAGTAAAACGAGGCGAGGGACCTATAAACCTCCCTTCATCATGCTATTTCATGATCTATTTTATAAAATAAAGATGTA
GAAAAAAGTAAGCGTAATAACCGCAAAACAAATGATTTAAAACATGGCACATAATGAGGAGATTAAGTTCGGTTTACGT
TTATTTTAGTACTAATTGTAACGTGAGACTACGTATCGGGAATTCTTAAGAGCAGCTTGCCAACATGGTGGAGCACGAC
ACTCTCGTCTACTCCAAGAATATCAAAGATACAGTCTCAGAAGACCAAAGGGCTATTGAGACTTTTCAACAAAGGGTAA
TATCGGGAAACCTCCTCGGATTCCATTGCCCAGCTATCTGTCACTTCATCAAAAGGACAGTAGAAAAGGAAGGTGGC
ACCTACAAATGCCATCATTGCGATAAAGGAAAGGCTATCGTTCAAGATGCCTCTGCCGACAGTGGTCCCAAAGATGGA
CCCCCACCCACGAGGAGCATCGTGGAAAAAGAAGACGTTCCAACCACGTCTTCAAAGCAAGTGGATTGATGTGAAC
ATGGTGGAGCACGACACTCTCGTCTACTCCAAGAATATCAAAGATACAGTCTCAGAAGACCAAAGGGCTATTGAGACT
TTTCAACAAAGGGTAATATCGGGAAACCTCCTCGGATTCCATTGCCCAGCTATCTGTCACTTCATCAAAAGGACAGTA
GAAAAGGAAGGTGGCACCTACAAATGCCATCATTGCGATAAAGGAAAGGCTATCGTTCAAGATGCCTCTGCCGACAG
TGGTCCCAAAGATGGACCCCCACCCACGAGGAGCATCGTGGAAAAAGAAGACGTTCCAACCACGTCTTCAAAGCAA
GTGGATTGATGTGATATCTCCACTGACGTAAGGGATGACGCACAATCCCACTATCCTTCGCAAGACCcTTCCTCTATAT
AAGGAAGTTCATTTCATTTGGAGAGGACACGCTGAAATCACCAGTCTCTCTCTACAAATCTATCTCTCTCGAGCTTTCG
CAGATCTGTCGATCGACCATGATTGAACAAGATGGATTGCACGCAGGTTCTCCGGCCGCTTGGGTGGAGAGGCTATT
CGGCTATGACTGGGCACAACAGACAATCGGCTGCTCTGATGCCGCCGTGTTtCGGCTGTCAGCGCAGGGGCGCCCG
GTTCTTTTTGTCAAGACCGACCTGTCCGGTGCCCTGAATGAACTCCAGGACGAGGCAGCGCGGCTATCGTGGCTGG
CCACGACGGGCGTTCCTTGCGCAGCTGTGCTCGACGTTGTCACTGAAGCGGGAAGGGACTGGCTGCTATTGGGCG
AAGTGCCGGGGCAGGATCTCCTGTCATCTCACCTTGCTCCTGCCGAGAAAGTATCCATCATGGCTGATGCAATGCGG
CGGCTGCATACGCTTGATCCGGCTACCTGCCCATTCGACCACCAAGCGAAACATCGCATCGAGCGAGCACGTACTC
GGATGGAAGCCGGTCTTGTCGATCAGGATGATCTGGACGAAGAGCATCAGGGGCTCGCGCCAGCCGAACTGTTCG
CCAGGCTCAAGGCGCGCATGCCCGACGGCGAGGATCTCGTCGTGACACATGGCGATGCCTGCTTGCCGAATATCAT
GGTGGAAAATGGCCGCTTTTCTGGATTCATCGACTGTGGCCGGCTGGGTGTGGCGGACCGCTATCAGGACATAGCG
TTGGCTACCCGTGATATTGCTGAAGAGCTTGGCGGCGAATGGGCTGACCGCTTCCTCGTGCTTTACGGTATCGCCGC
TCCCGATTCGCAGCGCATCGCCTTCTATCGCCTTCTTGACGAGTTCTTCTGAGCGGGACTCTGGGGTTCGGATCGAT
CCTCTAGCTAGAGTCGATCGACAAGCTCGAGTTTCTCCATAATAATGTGTGAGTAGTTCCCAGATAAGGGAATTAGGG
TTCCTATAGGGTTTCGCTCATGTGTTGAGCATATAAGAAACCCTTAGTATGTATTTGTATTTGTAAAATACTTCTATCAATA
AAATTTCTAATTCCTAAAACCAAAATCCAGTACTAAAATCCAGATCCCCCGAATTAATTAACACGTCTTCTCGAATTAGTT
TCTCCATAATAATGTGTGAGTAGTTCCCAGATAAGGGAATTAGGGTTCCTATAGGGTTTCGCTCATGTGTTGAGCATATA
AGAAACCCTTAGTATGTATTTGTATTTGTAAAATACTTCTATCAATAAAATTTCTAATTCCTAAAACCAAAATCCAGTACTA
AAATCCAGATCCCCCGAATTAATTCGGCGTTAATTCAGTACATTAAAAACGTCCGCAATGTGTTATTAAGTTGTCTAAGC
GTCAATTTGTTTACACCACAATATATCCTGCCACCAGCCAGCCAACAGCTCCCCGACCGGCAGCTCGGCACAAAATC
ACCACTCGATACAGGCAGCCCATCAGTCCGGGACGGCGTCAGCGGGAGAGCCGTTGTAAGGCGGCAGACTTTGCT
CATGTTACCGATGCTATTCGGAAGAACGGCAACTAAGCTGCCGGGTTTGAAACACGGATGATCTCGCGGAGGGTAGC
ATGTTGATTGTAACGATGACAGAGCGTTGCTGCCTGTGATCACCGCGGTTTCAAAATCGGCTCCGTCGATACTATGTT
ATACGCCAACTTTGAAAACAACTTTGAAAAAGCTGTTTTCTGGTATTTAAGGTTTTAGAATGCAAGGAACAGTGAATTG
GAGTTCGTCTTGTTATAATTAGCTTCTTGGGGTATCTTTAAATACTGTAGAAAAGAGGAAGGAAATAATAAATGGCTAAA
ATGAGAATATCACCGGAATTGAAAAAACTGATCGAAAAATACCGCTGCGTAAAAGATACGGAAGGAATGTCTCCTGCTA
AGGTATATAAGCTGGTGGGAGAAAATGAAAACCTATATTTAAAAATGACGGACAGCCGGTATAAAGGGACCACCTATGA
TGTGGAACGGGAAAAGGACATGATGCTATGGCTGGAAGGAAAGCTGCCTGTTCCAAAGGTCCTGCACTTTGAACGG
CATGATGGCTGGAGCAATCTGCTCATGAGTGAGGCCGATGGCGTCCTTTGCTCGGAAGAGTATGAAGATGAACAAAG
CCCTGAAAAGATTATCGAGCTGTATGCGGAGTGCATCAGGCTCTTTCACTCCATCGACATATCGGATTGTCCCTATACG
AATAGCTTAGACAGCCGCTTAGCCGAATTGGATTACTTACTGAATAACGATCTGGCCGATGTGGATTGCGAAAACTGG
GAAGAAGACACTCCATTTAAAGATCCGCGCGAGCTGTATGATTTTTTAAAGACGGAAAAGCCCGAAGAGGAACTTGTC
TTTTCCCACGGCGACCTGGGAGACAGCAACATCTTTGTGAAAGATGGCAAAGTAAGTGGCTTTATTGATCTTGGGAG
AAGCGGCAGGGCGGACAAGTGGTATGACATTGCCTTCTGCGTCCGGTCGATCAGGGAGGATATCGGGGAAGAACAG

TATGTCGAGCTATTTTTTGACTTACTGGGGATCAAGCCTGATTGGGAGAAAATAAAATATTATATTTTACTGGATGAATTG
TTTTAGTACCTAGAATGCATGACCAAAATCCCTTAACGTGAGTTTTCGTTCCACTGAGCGTCAGACCCCGTAGAAAAG
ATCAAAGGATCTTCTTGAGATCCTTTTTTTCTGCGCGTAATCTGCTGCTTGCAAACAAAAAAACCACCGCTACCAGCG
GTGGTTTGTTTGCCGGATCAAGAGCTACCAACTCTTTTTCCGAAGGTAACTGGCTTCAGCAGAGCGCAGATACCAAAT
ACTGTCCTTCTAGTGTAGCCGTAGTTAGGCCACCACTTCAAGAACTCTGTAGCACCGCCTACATACCTCGCTCTGCTA
ATCCTGTTACCAGTGGCTGCTGCCAGTGGCGATAAGTCGTGTCTTACCGGGTTGGACTCAAGACGATAGTTACCGGA
TAAGGCGCAGCGGTCGGGCTGAACGGGGGGTTCGTGCACACAGCCCAGCTTGGAGCGAACGACCTACACCGAACT
GAGATACCTACAGCGTGAGCTATGAGAAAGCGCCACGCTTCCCGAAGGGAGAAAGGCGGACAGGTATCCGGTAAGC
GGCAGGGTCGGAACAGGAGAGCGCACGAGGGAGCTTCCAGGGGGAAACGCCTGGTATCTTTATAGTCCTGTCGGG
TTTCGCCACCTCTGACTTGAGCGTCGATTTTTGTGATGCTCGTCAGGGGGGCGGAGCCTATGGAAAAACGCCAGCA
ACGCGGCCTTTTTACGGTTCCTGGCCTTTTGCTGGCCTTTTGCTCACATGTTCTTTCCTGCGTTATCCCCTGATTCTG
TGGATAACCGTATTACCGCCTTTGAGTGAGCTGATACCGCTCGCCGCAGCCGAACGACCGAGCGCAGCGAGTCAGT
GAGCGAGGAAGCGGAAGAGCGCCTGATGCGGTATTTTCTCCTTACGCATCTGTGCGGTATTTCACACCGCATATGGT
GCACTCTCAGTACAATCTGCTCTGATGCCGCATAGTTAAGCCAGTATACACTCCGCTATCGCTACGTGACTGGGTCAT
GGCTGCGCCCCGACACCCGCCAACACCCGCTGACGCGCCCTGACGGGCTTGTCTGCTCCCGGCATCCGCTTACAG
ACAAGCTGTGACCGTCTCCGGGAGCTGCATGTGTCAGAGGTTTTCACCGTCATCACCGAAACGCGCGAGGCAGGG
TGCCTTGATGTGGGCGCCGGCGGTCGAGTGGCGACGGCGCGGCTTGTCCGCGCCCTGGTAGATTGCCTGGCCGTA
GGCCAGCCATTTTTGAGCGGCCAGCGGCCGCGATAGGCCGACGCGAAGCGGCGGGGCGTAGGGAGCGCAGCGAC
CGAAGGGTAGGCGCTTTTTGCAGCTCTTCGGCTGTGCGCTGGCCAGACAGTTATGCACAGGCCAGGCGGGTTTTAA
GAGTTTTAATAAGTTTTAAAGAGTTTTAGGCGGAAAAATCGCCTTTTTTCTCTTTTATATCAGTCACTTACATGTGTGACC
GGTTCCCAATGTACGGCTTTGGGTTCCCAATGTACGGGTTCCGGTTCCCAATGTACGGCTTTGGGTTCCCAATGTAC
GTGCTATCCACAGGAAAGAGACTTTTTCGACCTTTTTCCCCTGCTAGGGCAATTTGCCCTAGCATCTGCTCCGTACAT
TAGGAACCGGCGGATGCTTCGCCCTCGATCAGGTTGCGGTAGCGCATGACTAGGATCGGGCCAGCCTGCCCCGCC
TCCTCCTTCAAATCGTACTCCGGCAGGTCATTTGACCCGATCAGCTTGCGCACGGTGAAACAGAACTTCTTGAACTC
TCCGGCGCTGCCACTGCGTTCGTAGATCGTCTTGAACAACCATCTGGCTTCTGCCTTGCCTGCGGCGCGGCGTGCC
AGGCGGTAGAGAAAACGGCCGATGCCGGGATCGATCAAAAAGTAATCGGGGTGAACCGTCAGCACGTCCGGGTTCT
TGCCTTCTGTGATCTCGCGGTACATCCAATCAGCTAGCTCGATCTCGATGTACTCCGGCCGCCCGGTTTCGCTCTTTA
CGATCTTGTAGCGGCTAATCAAGGCTTCACCCTCGGATACCGTCACCAGGCGGCCGTTCTTGGCCTTCTTCGTACGC
TGCATGGCAACGTGCGTGGTGTTTAACCGAATGCAGGTTTCTACCAGGTCGTCTTTCTGCTTTCCGCCATCGGCTCG
CCGGCAGAACTTGAGTACGTCCGCAACGTGTGGACGGAACACGCGGCCGGGCTTGTCTCCCTTCCCTTCCCGGTAT
CGGTTCATGGATTCGGTTAGATGGGAAACCGCCATCAGTACCAGGTCGTAATCCCACACACTGGCCATGCCGGCCG
GCCCTGCGGAAACCTCTACGTGCCCGTCTGGAAGCTCGTAGCGGATCACCTCGCCAGCTCGTCGGTCACGCTTCGA
CAGACGGAAAACGGCCACGTCCATGATGCTGCGACTATCGCGGGTGCCCACGTCATAGAGCATCGGAACGAAAAAA
TCTGGTTGCTCGTCGCCCTTGGGCGGCTTCCTAATCGACGGCGCACCGGCTGCCGGCGGTTGCCGGGATTCTTTG
CGGATTCGATCAGCGGCCGCTTGCCACGATTCACCGGGGCGTGCTTCTGCCTCGATGCGTTGCCGCTGGGCGGCC
TGCGCGGCCTTCAACTTCTCCACCAGGTCATCACCCAGCGCCGCGCCGATTTGTACCGGGCCGGATGGTTTGCGAC
CGTCACGCCGATTCCTCGGGCTTGGGGGTTCCAGTGCCATTGCAGGGCCGGCAGACAACCCAGCCGCTTACGCCT
GGCCAACCGCCCGTTCCTCCACACATGGGGCATTCCACGGCGTCGGTGCCTGGTTGTTCTTGATTTTCCATGCCGC
CTCCTTTAGCCGCTAAAATTCATCTACTCATTTATTCATTTGCTCATTTACTCTGGTAGCTGCGCGATGTATTCAGATAGC
AGCTCGGTAATGGTCTTGCCTTGGCGTACCGCGTACATCTTCAGCTTGGTGTGATCCTCCGCCGGCAACTGAAAGTT
GACCCGCTTCATGGCTGGCGTGTCTGCCAGGCTGGCCAACGTTGCAGCCTTGCTGCTGCGTGCGCTCGGACGGCC
GGCACTTAGCGTGTTTGTGCTTTTGCTCATTTTCTCTTTACCTCATTAACTCAAATGAGTTTTGATTTAATTTCAGCGGC
CAGCGCCTGGACCTCGCGGGCAGCGTCGCCCTCGGGTTCTGATTCAAGAACGGTTGTGCCGGCGGCGGCAGTGC
CTGGGTAGCTCACGCGCTGCGTGATACGGGACTCAAGAATGGGCAGCTCGTACCCGGCCAGCGCCTCGGCAACCT
CACCGCCGATGCGCGTGCCTTTGATCGCCCGCGACACGACAAAGGCCGCTTGTAGCCTTCCATCCGTGACCTCAAT
GCGCTGCTTAACCAGCTCCACCAGGTCGGCGGTGGCCCATATGTCGTAAGGGCTTGGCTGCACCGGAATCAGCACG
AAGTCGGCTGCCTTGATCGCGGACACAGCCAAGTCCGCCGCCTGGGGCGCTCCGTCGATCACTACGAAGTCGCGC
CGGCCGATGGCCTTCACGTCGCGGTCAATCGTCGGGCGGTCGATGCCGACAACGGTTAGCGGTTGATCTTCCCGC
ACGGCCGCCCAATCGCGGGCACTGCCCTGGGGATCGGAATCGACTAACAGAACATCGGCCCCGGCGAGTTGCAGG
GCGCGGGCTAGATGGGTTGCGATGGTCGTCTTGCCTGACCCGCCTTTCTGGTTAAGTACAGCGATAACCTTCATGCG
TTCCCCTTGCGTATTTGTTTATTTACTCATCGCATCATATACGCAGCGACCGCATGACGCAAGCTGTTTTACTCAAATAC
ACATCACCTTTTTAGACGGCGGCGCTCGGTTTCTTCAGCGGCCAAGCTGGCCGGCCAGGCCGCCAGCTTGGCATCA
GACAAACCGGCCAGGATTTCATGCAGCCGCACGGTTGAGACGTGCGCGGGCGGCTCGAACACGTACCCGGCCGC
GATCATCTCCGCCTCGATCTCTTCGGTAATGAAAAACGGTTCGTCCTGGCCGTCCTGGTGCGGTTTCATGCTTGTTC
CTCTTGGCGTTCATTCTCGGCGGCCGCCAGGGCGTCGGCCTCGGTCAATGCGTCCTCACGGAAGGCACCGCGCCG
CCTGGCCTCGGTGGGCGTCACTTCCTCGCTGCGCTCAAGTGCGCGGTACAGGGTCGAGCGATGCACGCCAAGCAG
TGCAGCCGCCTCTTTCACGGTGCGGCCTTCCTGGTCGATCAGCTCGCGGGCGTGCGCGATCTGTGCCGGGGTGAG
GGTAGGGCGGGGGCCAAACTTCACGCCTCGGGCCTTGGCGGCCTCGCGCCCGCTCCGGGTGCGGTCGATGATTA
GGGAACGCTCGAACTCGGCAATGCCGGCGAACACGGTCAACACCATGCGGCCGGCCGGCGTGGTGGTGTCGGCC
CACGGCTCTGCCAGGCTACGCAGGCCCGCGCCGGCCTCCTGGATGCGCTCGGCAATGTCCAGTAGGTCGCGGGT

GCTGCGGGCCAGGCGGTCTAGCCTGGTCACTGTCACAACGTCGCCAGGGCGTAGGTGGTCAAGCATCCTGGCCAG
CTCCGGGCGGTCGCGCCTGGTGCCGGTGATCTTCTCGGAAAACAGCTTGGTGCAGCCGGCCGCGTGCAGTTCGG
CCCGTTGGTTGGTCAAGTCCTGGTCGTCGGTGCTGACGCGGGCATAGCCCAGCAGGCCAGCGGCGGCGCTCTTGT
TCATGGCGTAATGTCTCCGGTTCTAGTCGCAAGTATTCTACTTTATGCGACTAAAACACGCGACAAGAAAACGCCAGG
AAAAGGGCAGGGCGGCAGCCTGTCGCGTAACTTAGGACTTGTGCGACATGTCGTTTTCAGAAGACGGCTGCACTGA
ACGTCAGAAGCCGACTGCACTATAGCAGCGGAGGGGTTGGATCAAAGTACTTTGATCCCGAGGGGAACCCTGTGGT
TGGCATGCACATACAAATGGACGAACGGATAAACCTTTTCACGCCCTTTTAAATATCCGTTATTCTAA

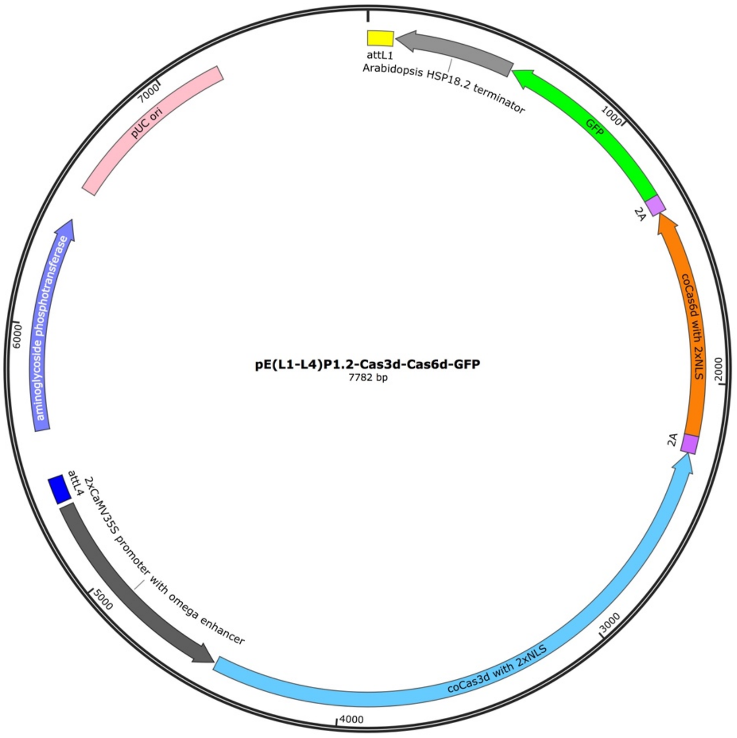

Supplementary Fig. 18. Plasmid vector pE(L1-L4)P1.2-Cas3d-Cas6d-GFP. Intermediate plasmid DNA to construct pMGTiD20.

1..96: attL1
106..552(complement): Arabidopsis HSP18.2 terminator
559..1275(complement): GFP
1276..1338(complement): 2A self-cleavage peptide
1339..2196(complement): coCas6d with 2xNLS
2197..2262(complement): 2A self-cleavage peptide
2263..4479(complement): coCas3d with 2xNLS
4489..5308(complement): 2x CaMV35S promoter with omega translational enhancer
5328..5423: attL4
5607..6416: Aminoglycoside phosphotransferase
6537..7210: pUC ori

>pE(L1-L4)P1.2-Cas3d-Cas6d-GFP
AAATAATGATTTTATTTTGACTGATAGTGACCTGTTCGTTGCAACAAATTGATGAGCAATGCTTTTTTATAATGCCAACTT
TGTACAAAAAAGCAGGCGGGGCCCGTCTCACGTTACAATTAGTACTAAAATAAACGTAAACCGAACTTAATCTCCTCAT
TATGTGCCATGTTTTAAATCATTTGTTTTGCGGTTATTACGCTTACTTTTTTCTACATCTTTATTTTATAAAATAGATCATGA
AATAGCATGATGAAGGGAGGTTTATAGGTCCCTCGCCTCGTTTTACTCTATGAGCCCATCTTATCTTTAATCATATTCCA
TAGTCCATACCATAGCACATACAGTAGTTATATGCTGCAGAAGAGATCCAACAAAACATTCACAATGGATTATAGAAACA
TTTGTTTATTCATTATAATGAAATCTTACATTCATTTAATATTAGAAAAAGCCACAAATTCATAACACAACAAGCCAAGAAA
AAAACACAAACTTAAGCACACAAACTTTTTATTTGACACACCAAATATTTCATCTTCATCTTCATATGAGCTCTTACTTGT
ACAGCTCGTCCATGCCGAGAGTGATCCCGGCGGCGGTCACGAACTCCAGCAGGACCATGTGATCGCGCTTCTCGTT
GGGGTCTTTGCTCAGGGCGGACTGGGTGCTCAGGTAGTGGTTGTCGGGCAGCAGCACGGGGCCGTCGCCGATGG
GGGTGTTCTGCTGGTAGTGGTCGGCGAGCTGCACGCTGCCGTCCTCGATGTTGTGGCGGATCTTGAAGTTCACCTT
GATGCCGTTCTTCTGCTTGTCGGCCATGATATAGACGTTGTGGCTGTTGTAGTTGTACTCCAGCTTGTGCCCCAGGAT
GTTGCCGTCCTCCTTGAAGTCGATGCCCTTCAGCTCGATGCGGTTCACCAGGGTGTCGCCCTCGAACTTCACCTCG
GCGCGGGTCTTGTAGTTGCCGTCGTCCTTGAAGAAGATGGTGCGCTCCTGGACGTAGCCTTCGGGCATGGCGGACT
TGAAGAAGTCGTGCTGCTTCATGTGGTCGGGGTAGCGGCTGAAGCACTGCACGCCGTAGGTCAGGGTGGTCACGA
GGGTGGGCCAGGGCACGGGCAGCTTGCCGGTGGTGCAGATGAACTTCAGGGTCAGCTTGCCGTAGGTGGCATCG
CCCTCGCCCTCGCCGGACACGCTGAACTTGTGGCCGTTTACGTCGCCGTCCAGCTCGACCAGGATGGGCACCACC
CCGGTGAACAGCTCCTCGCCCTTGCTCACTGGCCCGGGATTCTCCTCAACGTCACCGCATGTTAGCAGACTTCCTCT
GCCCTCTCCGGATCCTCTCTTGTCCTTAGAGATTCTTCTAGTCATTCCCATTCCCATAGTAGTCTTTCTTCCCACTCCA
GCATAAAGAGCGAAGTCAGCAAGAGCGTTGATAGCCTTGATCTTAGCTGGCTCCACCTCTCCAAGAAGTCTATAGTG
GATCTCTCCCACGCATCCGATAGACTGGTTCTTATAAGCCTCGTCAGCAAGCTTAGTCTGGATGTCGAAGAAAGATGG

GAAGATAGACTCAAGAGCGATAGAGTCGAATGGGATTCCAGAATATCTGTTCCATCTTCCAAGAAGAGAGTTGAACAC
AAGCTCTCTAGTTGGAAGAGCAGAGTCGAACTTTCCCTGTCTGAAAGTCACTGGAGTAGCGAAAAGGAAGTCGAACT
CTCTGTTAGTCTCAGAAGCGTTCTCATAAAGATACTGATAAGTGCAAGAGTTAGCCCAAGGCTGCACAGACTGTGGAG
TAGCAAGCACAGAAGTGATCACAAGGTCAGCAGATCCAAGGTGCCAAGGCTGCTTTGGGTTAAGGTTAAGCCAAAGA
GAAGTAAGCTTTCCGAAAAGTCTGTCGTCAAGAAGAGAGATTCTCCACCAGCATGGAGTCTCAGAAGCGATCTCTCT
CTCGTGTCTCCACTGTGGAGAGTTGATAGTCTTTCCTCCAGACTGCATCTGAAGTGGAGAAAGAGTGAAAGCCTTGT
CAGCCTCAGCAGTGTGAAGGATGTTTCCAAGCTCCTGGTCCACAGAAGACACAAGAGTAAGGAAAAGAGCGTGAAG
GTGTCTTCCAGTAAGGAAGTTTGGATAGATTGGAGATCTTGGAGTAAGGTTAAGCACAAGAGAATATGGTCCAGAAAC
CTTCCTTTTCTTTTTCGGGTCGAATTCAGGGCCAGGGTTTTCCTCCACGTCGCCGCAGGTCAGCAGGCTGCCTCTG
CCCTCAGAGCCGCAGATCCACACCTCTCCTCCCTTTCCCTTGAATCTGTGAGCAAGAGTGTCAAGAAGAAGAGCAG
CCTGTCCGATAGCGATAGAATATGGAGAAAGTGGAGAGTGAAGAGAAGACTCGTCAGAGATTGGATAGATTCCGAAG
TGCATTGGAAGCTGAAGTCTCTGTCTCACAGCAGCCACTGGATAAGCGAACACGAAAGACACAAGAGCAAGATCTCT
AAGCTCTCTTGAGATTCTGTCAAGCCAAGGGTTGTCTGGCTGCCACACTCCGATTCCAGTAAGCACCTGCACCTTCC
AAGCAGAAGCGATCTCTCCAAGATCTCCGTCATAAGTGAATCTCCAGTTAAGTCTCTCCTCTCTATATCCCTTAAGGTT
AAGGAAAGCAAGGCAATAGTCGAATCTTCTTCTAGCGATTGGAGTCTTAGTAGCCTCGATCTGTCTCTTGAACTCTCC
CTTTCTCCACACCTCCACCTCAAGGTTTCCAAGGATAGATGGAAGGTCATAAGTCTTGAATCTGTCGCACTCCTCTGG
CTCAGTAGAGTCATAAAGTCCGCAAAGAAGTGGAGAAGATCCTCTGAAAGAAGAAGCGTCCTCGAAGATTGGGTTTC
CGTTCTTTCCAGAAAGCTCCTTCCACTCGTCAGCCCATCCCTTCACTCTTCCAGCCACTCTCTTAAGAGAAGTGTCGA
ACACCTCCTCGCACTCCTGCTGGAACTTCTGTCTTGACTCTCCATACTGAGACTTGATCTTTGGAGATCCAAGGTTCC
ACCAAAGCTGGAAAGACTGCACAGCTCCCCATCTCTGATAATATCCCTCGAAGTTGTTGATGTGTCTATAGTTAGACTT
GATAGCCTCCTGAAGCTGGATTCTGTCATATCTCTCTCCCACCTGAAGTGGAGCGTCCTTCTTCTCGAAAAGTCTCTC
AAGGAAGAACTTTGGCACAAGAGCGATAGCAGTGAAGTTAGTGAACTTCACAGCAGTTCCCTTTCTGTCATATCCAGA
GTGTCTTCCAAGTCTTCCGAATCTCTGGATGAAGTTTCCAGCGTCAGAAGACTCGAAGATAAGGAAGTTGATCTTGAA
GTCCACTCCCACGTCGATAGTAGAAGTTCCGATCACAAGATCTCTGTTAAGAGAAGCAAGTTTCTCTCTAGTTCCAGA
AAGTCCAGTGTTCTCTCCCACAGTAAGTCCGATAGTAGCAAGAAGCTCTCTGAAGATTGGAAGAAGTCTCTTCACAGA
AGCGATAGAGTTAAGGATGATAGCTCCCTTAGATCCTGGATATCTCTTGAAATAGTCAAGGATTCTCTCCTTGTTCTCC
TTAAGCCAGTTCTCAGAAGTCTGGAAAGAAGATGGAAGTGGGATGAAAGAAAGCTCGATCTCAGAAGTCACCTGTCT
CCATCCCTGCTGAGCAAGAGAGTTAGCCTCCTCTGGAGTGTCTGGGAATCTATACTTTCCCTCTCTCACTGGGTCGAT
AGATCTATATCTGAATCCAGCCTTGTCAAGTCTCTTAAGAAGTCCCTCGTCTGGAGTAGCAGAAAGGAAAAGATATCTC
TTTCCTCTGTTAGCTCTTCTGATAAGAAGCATAGTGTTGATGATAGAAGCCACCTGTGGAGTTCCGAACACGTGGAAC
TCGTCGAAAAGGAAAAGGTCGAAGTGCTTGTCGATTCTGTTCCAAAGCTGGTCTGGGTTGTCATATGGAGTAAGATAA
GCAGCTCTGTGAAGATAGTGGAAGATGTCTGGGTTAGTAAGAAGGATCTCAGACTGCTTAGATCTAGTCTCAAGAGC
GATAGCCTTAGTCTTTCCGTCTCTCTCAGCATAAAGCTCAAGCTCTGGTCCAGTAAGAAGGTTCACTCTTGGCTGGTC
AGTTGGCTGGAATCTCTGGATATAAGCCTCGATCTGTCCTCTCTGATCTCTAGCAAGCTCGTTAGTTGGATAAAGTCC
CATGATTGGGAAATATCCCTGAAGAGTTCTAAGATAAGCAGCAAGAGACTTTCCGTCTCCAGTCATAGCAGTGTTGAA
GATCACGTCGATGTCTGGGTCGTTAAGAGCCTTCCAAGTCTCCACCTGGTGCCAAGAAAGTCTCCATCCCTGTGGCA
CCTTGATTCCGTCTGGGATCTCGTCAGCTGGGCAAGAATACACTGGCTTAAGAGTCACCTGATAGTTTCCTCCAGAAA
CCTTGCGCTTCTTCTTAGGGTCTACCTTCCGCTTCTTCTTTGGGTCCATGGTGCTTGTAATTGTAAATAGTAATTGTAAT
GTTGTTTGTTGTTTGTTGTTGTTGGTAATTGTTGTAAAAATACTCTAGACCGGCCTCTCCAAATGAAATGAACTTCCTTA
TATAGAGGAAGGGTCTTGCGAAGGATAGTGGGATTGTGCGTCATCCCTTACGTCAGTGGAGATATCACATCAATCCAC
TTGCTTTGAAGACGTGGTTGGAACGTCTTCTTTTTCCACGATGCTCCTCGTGGGTGGGGGTCCATCTTTGGGACCAC
TGTCGGCAGAGGCATCTTGAACGATAGCCTTTCCTTTATCGCAATGATGGCATTTGTAGGTGCCACCTTCCTTTTCTAC
TGTCCTTTTGATGAAGTGACAGATAGCTGGGCAATGGAATCCGAGGAGGTTTCCCGATATTACCCTTTGTTGAAAAGT
CTCAATAGCCCTTTGGTCTTCTGAGACTGTATCTTTGATATTCTTGGAGTAGACGAGAGTGTCGTGCTCCACCATGTTC
ACATCAATCCACTTGCTTTGAAGACGTGGTTGGAACGTCTTCTTTTTCCACGATGCTCCTCGTGGGTGGGGGTCCAT
CTTTGGGACCACTGTCGGCAGAGGCATCTTGAACGATAGCCTTTCCTTTATCGCAATGATGGCATTTGTAGGTGCCAC
CTTCCTTTTCTACTGTCCTTTTGATGAAGTGACAGATAGCTGGGCAATGGAATCCGAGGAGGTTTCCCGATATTACCCT
TTGTTGAAAAGTCTCAATAGCCCTTTGGTCTTCTGAGACTGTATCTTTGATATTCTTGGAGTAGACGAGAGTGTCGTGC
TCCACCATGTTGGCTTGGCGCGCCATTAACACCCAACTTTTCTATACAAAGTTGGCATTATAAGAAAGCATTGCTTATC
AATTTGTTGCAACGAACAGGTCACTATCAGTCAAAATAAAATCATTATTTGCCATCCAGCTGCAGGGCGGCCGCGATAT
CCCCTATAGTGAGTCGTATTACATGGTCATAGCTGTTTCCTGGCAGCTCTGGCCCGTGTCTCAAAATCTCTGATGTTAC
ATTGCACAAGATAAAAATATATCATCATGAACAATAAAACTGTCTGCTTACATAAACAGTAATACAAGGGGTGTTATGAGC
CATATTCAACGGGAAACGTCGAGGCCGCGATTAAATTCCAACATGGATGCTGATTTATATGGGTATAAATGGGCTCGCG
ATAATGTCGGGCAATCAGGTGCGACAATCTATCGCTTGTATGGGAAGCCCGATGCGCCAGAGTTGTTTCTGAAACATG
GCAAAGGTAGCGTTGCCAATGATGTTACAGATGAGATGGTCAGACTAAACTGGCTGACGGAATTTATGCCTCTTCCGA
CCATCAAGCATTTTATCCGTACTCCTGATGATGCATGGTTACTCACCACTGCGATCCCCGGAAAAACAGCATTCCAGG
TATTAGAAGAATATCCTGATTCAGGTGAAAATATTGTTGATGCGCTGGCAGTGTTCCTGCGCCGGTTGCATTCGATTCC
TGTTTGTAATTGTCCTTTTAACAGCGATCGCGTATTTCGTCTCGCTCAGGCGCAATCACGAATGAATAACGGTTTGGTT
GATGCGAGTGATTTTGATGACGAGCGTAATGGCTGGCCTGTTGAACAAGTCTGGAAAGAAATGCATAAACTTTTGCCA
TTCTCACCGGATTCAGTCGTCACTCATGGTGATTTCTCACTTGATAACCTTATTTTTGACGAGGGGAAATTAATAGGTT

GTATTGATGTTGGACGAGTCGGAATCGCAGACCGATACCAGGATCTTGCCATCCTATGGAACTGCCTCGGTGAGTTTT
CTCCTTCATTACAGAAACGGCTTTTTCAAAAATATGGTATTGATAATCCTGATATGAATAAATTGCAGTTTCATTTGATGC
TCGATGAGTTTTTCTAATCAGAATTGGTTAATTGGTTGTAACACTGGCAGAGCATTACGCTGACTTGACGGGACGGCG
CAAGCTCATGACCAAAATCCCTTAACGTGAGTTACGCGTCGTTCCACTGAGCGTCAGACCCCGTAGAAAAGATCAAA
GGATCTTCTTGAGATCCTTTTTTTCTGCGCGTAATCTGCTGCTTGCAAACAAAAAAACCACCGCTACCAGCGGTGGTT
TGTTTGCCGGATCAAGAGCTACCAACTCTTTTTCCGAAGGTAACTGGCTTCAGCAGAGCGCAGATACCAAATACTGTT
CTTCTAGTGTAGCCGTAGTTAGGCCACCACTTCAAGAACTCTGTAGCACCGCCTACATACCTCGCTCTGCTAATCCTG
TTACCAGTGGCTGCTGCCAGTGGCGATAAGTCGTGTCTTACCGGGTTGGACTCAAGACGATAGTTACCGGATAAGGC
GCAGCGGTCGGGCTGAACGGGGGGTTCGTGCACACAGCCCAGCTTGGAGCGAACGACCTACACCGAACTGAGATA
CCTACAGCGTGAGCTATGAGAAAGCGCCACGCTTCCCGAAGGGAGAAAGGCGGACAGGTATCCGGTAAGCGGCAG
GGTCGGAACAGGAGAGCGCACGAGGGAGCTTCCAGGGGGAAACGCCTGGTATCTTTATAGTCCTGTCGGGTTTCGC
CACCTCTGACTTGAGCGTCGATTTTTGTGATGCTCGTCAGGGGGGCGGAGCCTATGGAAAAACGCCAGCAACGCGG
CCTTTTTACGGTTCCTGGCCTTTTGCTGGCCTTTTGCTCACATGTTCTTTCCTGCGTTATCCCCTGATTCTGTGGATAA
CCGTATTACCGCCTTTGAGTGAGCTGATACCGCTCGCCGCAGCCGAACGACCGAGCGCAGCGAGTCAGTGAGCGA
GGAAGCGGAAGAGCGCCCAATACGCAAACCGCCTCTCCCCGCGCGTTGGCCGATTCATTAATGCAGCTGGCACGAC
AGGTTTCCCGACTGGAAAGCGGGCAGTGAGCGCAACGCAATTAATACGCGTACCGCTAGCCAGGAAGAGTTTGTAG
AAACGCAAAAAGGCCATCCGTCAGGATGGCCTTCTGCTTAGTTTGATGCCTGGCAGTTTATGGCGGGCGTCCTGCCC
GCCACCCTCCGGGCCGTTGCTTCACAACGTTCAAATCCGCTCCCGGCGGATTTGTCCTACTCAGGAGAGCGTTCAC
CGACAAACAACAGATAAAACGAAAGGCCCAGTCTTCCGACTGAGCCTTTCGTTTTATTTGATGCCTGGCAGTTCCCTA
CTCTCGCGTTAACGCTAGCATGGATGTTTTCCCAGTCACGACGTTGTAAAACGACGGCCAGTCTTAAGCTCGGGCCC
C

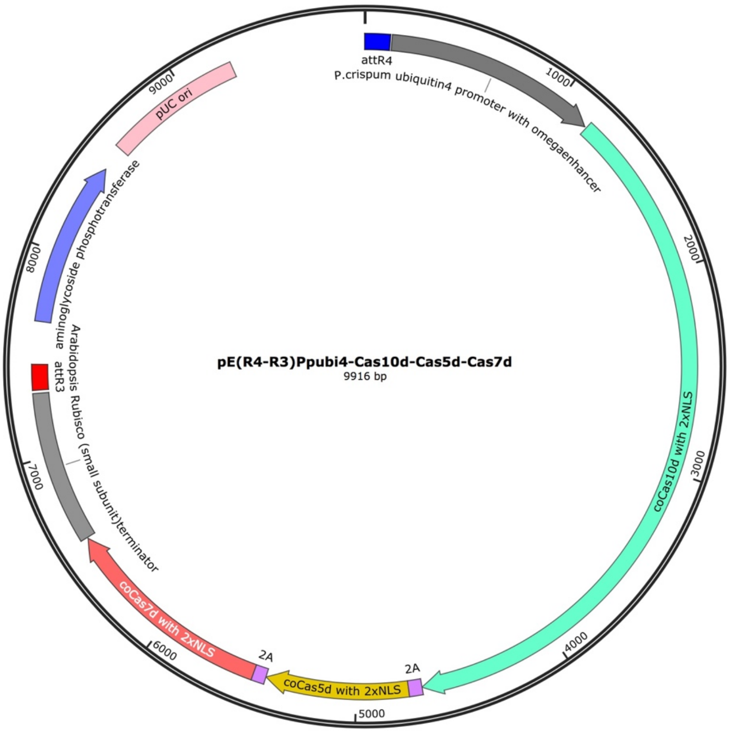

Supplementary Fig. 19. Plasmid vector pE(R4-R3)Ppubi4-Cas10d-Cas5d-Cas7d. Intermediate plasmid DNA to construct pMGTiD20.

1..124: attR4
133..1172: P.crispum ubiquitin4 promoter with omega translational enhancer
1182..4679: coCas10d with 2xNLS
4680..4745: 2A self-cleavage peptide
4746..5444: coCas5d with 2xNLS
5445..5507: 2A self-cleavage peptide
5508..6560: coCas7d with 2xNLS
6567..7309: Arabidopsis Rubisco (small subunit) terminator
7325..7448: attR3
7656..8465: Aminoglycoside phosphotransferase
8586..9259: pUC ori

>pE(R4-R3)Ppubi4-Cas10d-Cas5d-Cas7d
CATAGTGACTGGATATGTTGTGTTTTACAGTATTATGTAGTCTGTTTTTTATGCAAAATCTAATTTAATATATTGATATTTATA
TCATTTTACGTTTCTCGTTCAACTTTTCTATACAAAGTTGGGCGCGCCAAAAATTACGGATATGAATATAGGCATATCCG
TATCCGAATTATCCGTTTGACAGCTAGCAACGATTGTACAATTGCTTCTTTAAAAAAGGAAGAAAGAAAGAAAGAAAAG
AATCAACATCAGCGTTAACAAACGGCCCCGTTACGGCCCAAACGGTCATATAGAGTAACGGCGTTAAGCGTTGAAAG
ACTCCTATCGAAATACGTAACCGCAAACGTGTCATAGTCAGATCCCCTCTTCCTTCACCGCCTCAAACACAAAAATAAT
CTTCTACAGCCTATATATACAACCCCCCCTTCTATCTCTCCTTTCTCACAATTCATCATCTTTCTTTCTCTACCCCCAATT
TTAAGAAATCCTCTCTTCTCCTCTTCATTTTCAAGGTAAATCTCTCTCTCTCTCTCTCTCTCTGTTATTCCTTGTTTTAAT
TAGGTATGTATTATTGCTAGTTTGTTAATCTGCTTATCTTATGTATGCCTTATGTGAATATCTTTATCTTGTTCATCTCATCC
GTTTAGAAGCTATAAATTTGTTGATTTGACTGTGTATCTACACGTGGTTATGTTTATATCTAATCAGATATGAATTTCTTCA
TATTGTTGCGTTTGTGTGTACCAATCCGAAATCGTTGATTTTTTTCATTTAATCGTGTAGCTAATTGTACGTATACATATG
GATCTACGTATCAATTGTTCATCTGTTTGTGTTTGTATGTATACAGATCTGAAAACATCACTTCTCTCATCTGATTGTGTT
GTTACATACATAGATATAGATCTGTTATATCATTTTTTTATTAATTGTGTATATATATATGTGCATAGATCTGGATTACATGAT
TGTGATTATTTACATGATTTTGTTATTTACGTATGTATATATGTAGATCTGGACTTTTTGGAGTTGTTGACTTGATTGTATTT
GTGTGTGTATATGTGTGTTCTGATCTTGATATGTTATGTATGTGCATCTAGAGTATTTTTACAACAATTACCAACAACAAC
AAACAACAAACAACATTACAATTACTATTTACAATTACAAGCACCATGGACCCTAAGAAAAAGAGAAAGGTTTCTGGAC
CAAAGAAGCAGAAGAAGCTTGAGGAGACTGGACAGCTTAACCTTTTCGACAACACTACTGAGATCGACGACGAGGA
CCTTGACTTCGAGTTCGAGGACATCGACCTTGAGTCTCTTGTGTCTGAGGACCTTGGAATCACTGAGTCTGTGTCTG
ACAGAAGAGTGGAGACTGTGAGACAGCTTCTTACTCTTAAGCTTCTTAGAGAGGCTATCAGAGCTGAGAACCCAGAC
GACAGAGTGATGGCTGACTTCGCTGAGATGGTGCTTCCAAACCTTCTTAGACTTGCTATCGGAGTGACTGCTAAGGG
AGGAAACTTCATCGAGGCTGTGGACAGAGGAAGAGAGCTTAGAAACAAGCCAAAGGCTAAGAGAGACAACGCTGGA

GATCAGTCTCTTAACACTCACCTTCTTAACGGACTTTTCCCAGCTAACCTTATCGAGAAGAGACTTCAGAAGCTTAACA
CTACTGTGAGAAGAATCATCAAGGAGTTCGAGAGAAGACTTGCTATCGCTGGATTCCTTGTGCACGACTTCGAGAAG
TTCTCTTATGACAGATTCCCATCTATGTCTGAGAGATATATCCAGATCCAGAGAGACTTCATCCAGGACCCATTCAAGA
ACCAGGACCCAAGAAAGCTTTCAAGAGAGGAGCACAGAGAGATCCTTCAGGTGCTTATCCCAGAGCTTGGACTTGA
CAGATTCCTTTTCCCAGACAACCCAGAGAGATGGCTTGAGTATCTTGACGACCTTCTTTATATCGCTAAGAACACTCAG
AGAAGAAACGACACTGACCTTAACACTTCTGAGGACGGACTTAACGTGAGACTTAACGACAGAGTGATCGAGTCTCT
TTGCGACCTTGCTTGCCTTGCTGACAGACTTGCTTCTATCATCAAGCACCCACACGACGCTGAGAAGGCTTCTCTTC
AGGACCTTCTTTATTCTCTTTCTGACGGAGAGCTTAAGTTCACTTATCACTCTATCGCTGAGAACAGAGGAGTGCTTAC
TAACGTGCTTAACAACGCTGTGATGGAGGCTCACCAGGAGCTTGACTATCAGCCACTTCTTTATCTTCCAACTGGAGT
GGTGTATATCGCTCCAAAGAACGCTCCAGAGGTGTCTCTTGAGACTCTTCCAAACAGAGTGGTGGACACTATCAAGT
CTCTTTGCTCTGGAGAGCTTCAGAGAAAGCAGACTGGATTCGGAAGAGACGGAAAGGGtATGAAGTATGCTGACTATT
ATTCTCAGTTCTTCGACGACGCTGGACTTATGAGAGCTGCTCTTAACGCTACTCTTAGAATCCTTGGAGACAACAAGG
CTTCTGTGGCTAGATCTAGAGGAGAGAACCTTATCAAGTTCCAGCAGCAGGGAGTGCTTCCAACTGACTATGACTTC
CACTGCGAGGACGACATCAGAATCGACAGACTTGCTGAGTTCGGAGACGTGGTGACTAGAAAGATCTGGGGAGACA
GACTTGAGAAGATCGAGCAGGCTAGAAAGCTTCAGAAGAACCTTCCAGCTCCACCAGACCTTGACCTTATCTCTGAG
ATCGCTCACTATTGGAACCTTGAGAACTATCTTCCACAGATCAGAGCTATCAAGAGAATCAACGAGTCTCTTAAGGAG
CTTAAGCTTAAGGGAAACACTGGAGGAGTGCCATATGAGTGGTATTATCTTGCTGCTCAGTATCTTAAGCAGCACCCA
GGAATCGAGGACATCAGACCAGTGGCTGAGGACCTTATCGCTTTCCTTGCTGCTAAGATCGCTGCTATCGTGGCTGG
ATATAACCTTCCAGACGGATGGGAGGACCTTAGAGAGTGGGTGAACCAGGTGGTGCAGCTTCCAGGAAGAGAGCTT
GCTCACTCTATCGAGACTTTCCAGAAGGAGCTTAACCACTATAACGCTGCTAAGAAGCAGGGAAGAGGAAGACAGCT
TCTTTGCTCTATCTCTCACTCTCCATATTCTGTGTCTGAGCAGATGGAGTCTGCTGTGCTTTTCACTCCACAGGTGTAT
ACTAACAAGCAGATGCTTGCTGGATCTAACGCTAAGAGAAACATCTCTTCTATCGCTGGAACTGAGATGATGCTTAGA
CAGATCCTTATGAACCAGACTCAGGCTGTGGGAAAGAGATTCGAGGACGGAAAGTATAGATATCTTTATTTCTATCCAA
CTTATTATTTCACTCCAGAGACTAACTCTTTCCTTCAGAAGGCTTATGCTAACATCGCTCAGACTAGATTCGACTCTTCT
ATCAAGCTTCACTTCGTGGACAAGAACCTTGTGGCTAACTTCGACAGAACTAGATATCAGTCTGTGGACTCTTTCCTT
ATCGACGAGAAGCTTAGACAGAAGAAGGAGACTATCAACGAGGAGGAGGACGGAAAGAAGGACAGAACTTTCAAGC
TTTCTTATCCAGAGGACAAGCCACTTACTTTCTATTTCATGGCTCTTCCACCAGGAAGAAACCCAACTGACACTGAGT
CTTGGGTAATGCCAGCTTGGCTTGGACTTGCTTTCCCAATGATCCTTGACGTGAAGACTGTGGTGTCTGAGTCTCCA
ATCCCACCATATAGAGACGGAGCTGAGTTCGAGGAGACTGTGTTCCTTGACTCTGCTCCACAGGCTATCAGATCTCTT
ACTAGATGCGACAGATTCAGACTTGACAGAGTGCTTAACCCTTGGCAGGACAACGACGGAAAGAAGTATTCTGCTCC
ACTTAACACTCTTACTGCTGCTTATTCTATCCACCTTGACGTGAACTCTAAGCAGGGAAAGACTGGATATGACCCAAAC
TGGGGAAAGCTTACTGAGCTTGCTATCAACCTTGAGACTTCTCCACTTTATGTGTTCCACTATTTCAAGCAGTGGAAG
AGAGGAAAGGACGCTGACATCCCATCTGCTAACAGAATCGCTCTTTATCTTTATGACTTCTATCCATGCTTCGACCCAT
ATGTGCAGGCTAACAGAACTAACCTTACTATCGACATGACTGCTGAGTCTCCACTTAACCACCCAAAGAACCTTACTG
AGCTTTATAGACAGTTCTATAGAGCTAAGTCTTCTAAGGGAAAGCCAATCAAGGCTAACGCTATCCTTAAGCCAATCGA
CGAGGCTGCTGACATCATCCTTAAGGCTGACAAGGCTATCTCTGACGACCTTACTTCTCTTGTGGCTGCTAGACTTTT
CAAGCTTATGGACAGAGTGAGATCTCAGACTGCTGAGGGAAGATATGTGATCAAGGAGAGAGATCAGGAGAGAGAG
AAGATCCTTGACTTCGCTAAGTATTTCGTGAAGAACGTGTTCGAGGAGTCTTTCGAGTCTGACAGAGCTAGACTTGCT
GGAAGACAGCTTAACATCATCAGAGACACTTGCGAGTTCCTTTATAGACTTGAGATGGACAAGGAGAGAAGACAGAG
ACAGGTGCAGCCACTTGACACTTCTAACTCTTCTTCTGAGGAGGAGGAGGGGTCTGAGGGACGCGGCTCCCTGCTC
ACCTGTGGAGATGTGGAAGAGAACCCAGGCCCCGAATTCGACCCGAAAAAGAAAAGGAAGGTTTCTGGAGTGCACA
TCTATTCTTGCCAGCTTGAGCTTCACGACTCTCTTTATTATGCTACTAGAGAGATCGGAAGACTTTATGAGTCTGAGCC
AGTGATCCACAACTATGCTCTTTGCTATGCTCTTGGACTTGTGAACTCTGACTCTTATAGATATTTCTGCTCTGAGCAG
ATCCCACAGTATCAGGAGCACCTTAACCCACTTAACGAGGAGAAGATCTATGTGACTCCAGCTAGAGCTATCGCTCAC
ACTGCTGTGCTTAACACTTGGAAGTATGCTAACAACAACTATCACGTGGAGATGGAGAAGACTCAGAAGAACATCCCA
TCTTTCGGAAGAGCTAAGGAGATCGCTCCAGAGTCTATCTTCGAGTGCTTCATCATCTCTCACCACCCACTTCAGCTT
CCAAAGTGGATCAGACTTGGAAAGTGGATGTCTAAGGCTGAGGTGAAGCTTACTGAGCTTTCTCTTTCTAAGCAGAA
GGAGGACCTTTTCATCTATCCATATCCACTTAACCCACTTGACGTGATGTTCACTCACCAGGTTATCGGATATGACGTG
ATCAACATGCCACCAGTGTCTCTTATCAGAAACGTGAGAATGAGAGGAGAGTATTATCAGATCTCTGACAGACCAGAC
CTTAAGATCCCAGCTAGACTTTCTTATCACTTCGGAGGATCCGGAGAGGGCAGAGGAAGTCTGCTAACATGCGGTGA
CGTTGAGGAGAATCCCGGGCCAGACCCAAAGAAGAAGCGGAAGGTAGACCCTAAGAAGAAGCGCAAGGTTTCTGG
AACTTTCCTTACTTCTGTGGACGCTAAGTTCTTCCACTCTGAGATCCCATATAAGCCAATGGGAAAGTATGTGCACTTC
CTTACTATCAGAGTGACTGAGTCTTATCCACTTTTCCAGACTGACGGAGAGCTTAACAAGGCTAGAGTGAGAGCTGGA
ATCGACTCTAAGAAGACTATCTCAAGACTTTCTATGTTCAAGAGAAAGCAGTCTACTCCAGAGAGACTTGTGGGAAGA
GAGCTTCTTAGAAACTATGGACTTATCACTGCTGAGGAGTGCGAGTATAACGTGAAGTTCGCTATGAACAACGCTGAC
TGCATCATCTATGGATTCGCTATCGGAGACTCTGGATCTGAGAAGTCTAAGGTGGTGGTGGACACTGCTTTCTCTATC
ACTCCATTCGACGAGTCTCACGAGTCTTTCACTCTTAACGCTCCATATGAGAACGGAACTATGGCTTCTAAGGGAGAG
AACAACACTAAGGTGGGAGAGGTGACTTCAAGAATCAACCAGCAGGACCACATCAGACCACAGGTGTTCTTCCCATC
TATCGTGACTCTTAAGGACCCAACTGAGGCTTCTTTCCTTTATGTGTTCAACAACATCCTTAGAACTAGACACTATGGT
GCTCAGACTACTAGAACTGGAAGAGTGAGAAACGAGCTTATCGGAGTGATCTTCGCTGACGGAGAGATCGTGTCTAA

CCTTAGATGGACTCAGGCTATCTATGACAGACTTCCAGACGAGGTGCTTCACTCTATCGACCCACTTGACGAGGACC
TTGTGATGGAGAAGGCTACTGAGGCTATCCAGGCTCTTATGGCTGAGGAGTTCATCGTGCACACTGACTTCATCGGA
GAGAACTTCCAGCCACTTCTTACTGAGGTGAAGACTCTTACTGGAACTGAGGCTGGAATCCTTTCTGTGCTTGACCA
GGCTAACAAGGAGTCTAAGAAGTATTTCGAGCAGTATATCGAGAAGAAGAAGGCTGAGAAGAAGTAAGAGCTCTCCC
CTTTCTGGAATATTCAGCGTTGATTATTCTGGAACCCATTTCTATGTGGTCAATGCAAATTTAAGAAATTATTTGCCGACT
TAAAAGTTGAGGAACTATTGTTGAAAGTGAAAATGTTATTCCTATCAGTTTCTCTATAATTATAGTTATCATTTCATTTCAT
TTTTGCCCTTAGCTCTTTGAAATCTTATTTTTCGTTTAGCTCCTTTAAACAACATTGTGGCTCCTTTAAATTATCCTCATA
ATTCTTGCTTTTGACTCCCTAGACTAACCAATAAACTCTAATAAAAAAAGAAAAACTTGCTACATTGTTTTAAGAAATTTT
GCACATGAAAGCAATCAATCAAACCTCGATATTTAAAGAAAAGTTTATGTAAGGGAGTGTAACCATTTTTCAGATGACAT
AGCCATTGGAGATTTGGAAAAGGTAGTATATACAGAAAATTCAAATGCATCTTTTAAAATTATAAACATAAAACATGTATT
CCAAACCCCTAAGTGGGATATTAAGTCAAGAAATAGCATTACATAAAGCAAGGATCGACAAAGACTGAAATTTGTCAAG
CATGAAGTTACTAAATTTTGGAAATTTTGTTTACGTTAAATTTGATCATTGGTTATGCATTAAAAGCTGAAACAGTTATTTA
GAAGGAAAAGTAGTTAACGTTCCCAAAAAGTTCTAAATAAAACTAGATTCCAAATGATAATGAAAACGTCGCATCTACTA
TATGTTTGGAATCCTGCAGGGATTAAACAACTTTGTATAATAAAGTTGAACGAGAAACGTAAAATGATATAAATATCAATA
TATTAAATTAGATTTTGCATAAAAAACAGACTACATAATACTGTAAAACACAACATATCCAGTCACTATGAATCAACTACTT
AGATGGTATTAGTGACCTGTACTGCAGGGCGGCCGCGATATCCCCTATAGTGAGTCGTATTACATGGTCATAGCTGTTT
CCTGGCAGCTCTGGCCCGTGTCTCAAAATCTCTGATGTTACATTGCACAAGATAAAAATATATCATCATGAACAATAAAA
CTGTCTGCTTACATAAACAGTAATACAAGGGGTGTTATGAGCCATATTCAACGGGAAACGTCGAGGCCGCGATTAAATT
CCAACATGGATGCTGATTTATATGGGTATAAATGGGCTCGCGATAATGTCGGGCAATCAGGTGCGACAATCTATCGCTT
GTATGGGAAGCCCGATGCGCCAGAGTTGTTTCTGAAACATGGCAAAGGTAGCGTTGCCAATGATGTTACAGATGAGA
TGGTCAGACTAAACTGGCTGACGGAATTTATGCCTCTTCCGACCATCAAGCATTTTATCCGTACTCCTGATGATGCATG
GTTACTCACCACTGCGATCCCCGGAAAAACAGCATTCCAGGTATTAGAAGAATATCCTGATTCAGGTGAAAATATTGTT
GATGCGCTGGCAGTGTTCCTGCGCCGGTTGCATTCGATTCCTGTTTGTAATTGTCCTTTTAACAGCGATCGCGTATTT
CGTCTCGCTCAGGCGCAATCACGAATGAATAACGGTTTGGTTGATGCGAGTGATTTTGATGACGAGCGTAATGGCTG
GCCTGTTGAACAAGTCTGGAAAGAAATGCATAAACTTTTGCCATTCTCACCGGATTCAGTCGTCACTCATGGTGATTT
CTCACTTGATAACCTTATTTTTGACGAGGGGAAATTAATAGGTTGTATTGATGTTGGACGAGTCGGAATCGCAGACCGA
TACCAGGATCTTGCCATCCTATGGAACTGCCTCGGTGAGTTTTCTCCTTCATTACAGAAACGGCTTTTTCAAAAATATG
GTATTGATAATCCTGATATGAATAAATTGCAGTTTCATTTGATGCTCGATGAGTTTTTCTAATCAGAATTGGTTAATTGGT
TGTAACACTGGCAGAGCATTACGCTGACTTGACGGGACGGCGCAAGCTCATGACCAAAATCCCTTAACGTGAGTTAC
GCGTCGTTCCACTGAGCGTCAGACCCCGTAGAAAAGATCAAAGGATCTTCTTGAGATCCTTTTTTTCTGCGCGTAATC
TGCTGCTTGCAAACAAAAAAACCACCGCTACCAGCGGTGGTTTGTTTGCCGGATCAAGAGCTACCAACTCTTTTTCC
GAAGGTAACTGGCTTCAGCAGAGCGCAGATACCAAATACTGTTCTTCTAGTGTAGCCGTAGTTAGGCCACCACTTCAA
GAACTCTGTAGCACCGCCTACATACCTCGCTCTGCTAATCCTGTTACCAGTGGCTGCTGCCAGTGGCGATAAGTCGT
GTCTTACCGGGTTGGACTCAAGACGATAGTTACCGGATAAGGCGCAGCGGTCGGGCTGAACGGGGGGTTCGTGCA
CACAGCCCAGCTTGGAGCGAACGACCTACACCGAACTGAGATACCTACAGCGTGAGCTATGAGAAAGCGCCACGCT
TCCCGAAGGGAGAAAGGCGGACAGGTATCCGGTAAGCGGCAGGGTCGGAACAGGAGAGCGCACGAGGGAGCTTC
CAGGGGGAAACGCCTGGTATCTTTATAGTCCTGTCGGGTTTCGCCACCTCTGACTTGAGCGTCGATTTTTGTGATGCT
CGTCAGGGGGGCGGAGCCTATGGAAAAACGCCAGCAACGCGGCCTTTTTACGGTTCCTGGCCTTTTGCTGGCCTTT
TGCTCACATGTTCTTTCCTGCGTTATCCCCTGATTCTGTGGATAACCGTATTACCGCCTTTGAGCTTTCCTGCGTTATC
CCCTGATTCTGTGGATAACCGTATTACCGCCTTTGAGTGAGCTGATACCGCTCGCCGCAGCCGAACGACCGAGCGCA
GCGAGTCAGTGAGCGAGGAAGCGGAAGAGCGCCCAATACGCAAACCGCCTCTCCCCGCGCGTTGGCCGATTCATT
AATGCAGCTGGCACGACAGGTTTCCCGACTGGAAAGCGGGCAGTGAGCGCAACGCAATTAATACGCGTACCGCTAG
CCAGGAAGAGTTTGTAGAAACGCAAAAAGGCCATCCGTCAGGATGGCCTTCTGCTTAGTTTGATGCCTGGCAGTTTA
TGGCGGGCGTCCTGCCCGCCACCCTCCGGGCCGTTGCTTCACAACGTTCAAATCCGCTCCCGGCGGATTTGTCCTA
CTCAGGAGAGCGTTCACCGACAAACAACAGATAAAACGAAAGGCCCAGTCTTCCGACTGAGCCTTTCGTTTTATTTG
ATGCCTGGCAGTTCCCTACTCTCGCGTTAACGCTAGCATGGATGTTTTCCCAGTCACGACGTTGTAAAACGACGGCC
AGTCTTAAGCTCGGGCCCCTACAGGTCACTAATACCATCTAAGTAGTTGATT

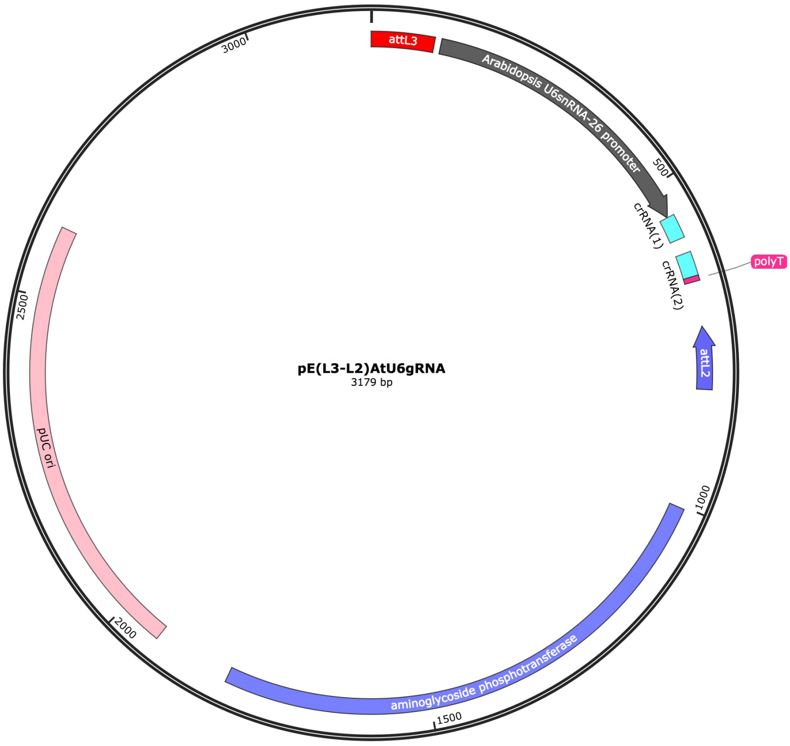

Supplementary Fig. 20. Plasmid vector pE(L3-L2)AtU6gRNA. Intermediate plasmid DNA to construct pMGTiD20.

1..96: attL3
107..551: Arabidopsis U6snRNA-26 promoter
552..588: crRNA(1)
612..648: crRNA(2)
649..656: polyT
725..820: attL2
1004..1813: Aminoglycoside phosphotransferase
1934..2607: pUC ori

>pE(L3-L2)AtU6gRNA
AAATAATGATTTTATTTTGACTGATAGTGACCTGTTCGTTGCAACAAATTGATGAGCAATGCTTTTTTATAATGCCAACTT
TGTATAATAAAGTTGGGCCTGCAGGCTTCGTTGAACAACGGAAACTCGACTTGCCTTCCGCACAATACATCATTTCTTC
TTAGCTTTTTTTCTTCTTCTTCGTTCATACAGTTTTTTTTTGTTTATCAGCTTACATTTTCTTGAACCGTAGCTTTCGTTTT
CTTCTTTTTAACTTTCCATTCGGAGTTTTTGTATCTTGTTTCATAGTTTGTCCCAGGATTAGAATGATTAGGCATCGAAC
CTTCAAGAATTTGATTGAATAAAACATCTTCATTCTTAAGATATGAAGATAATCTTCAAAAGGCCCCTGGGAATCTGAAA
GAAGAGAAGCAGGCCCATTTATATGGGAAAGAACAATAGTATTTCTTATATAGGCCCATTTAAGTTGAAAACAATCTTCA
AAAGTCCCACATCGCTTAGATAAGAAAACGAAGCTGAGTTTATATACAGCTAGAGTCGAAGTAGTGATTGTTCCAATTA
ATCTTAAGCCCTATTAGGGATTGAAACGGAGACCCTCAATTGTCGGTCTCGTTCCAATTAATCTTAAGCCCTATTAGGG
ATTGAAACTTTTTTTTGCAAAATTTTCCAGATCGATTTCTTCTTCCTCTGTTCTTCGGCGTTCAATTTCTTACGTATTAAT
ACCCAGCTTTCTTGTACAAAGTTGGCATTATAAGAAAGCATTGCTTATCAATTTGTTGCAACGAACAGGTCACTATCAG
TCAAAATAAAATCATTATTTGCCATCCAGCTGCAGGGCGGCCGCGATATCCCCTATAGTGAGTCGTATTACATGGTCAT
AGCTGTTTCCTGGCAGCTCTGGCCCGTGTCTCAAAATCTCTGATGTTACATTGCACAAGATAAAAATATATCATCATGA
ACAATAAAACTGTCTGCTTACATAAACAGTAATACAAGGGGTGTTATGAGCCATATTCAACGGGAAACGTCGAGGCCG
CGATTAAATTCCAACATGGATGCTGATTTATATGGGTATAAATGGGCTCGCGATAATGTCGGGCAATCAGGTGCGACAA
TCTATCGCTTGTATGGGAAGCCCGATGCGCCAGAGTTGTTTCTGAAACATGGCAAAGGTAGCGTTGCCAATGATGTTA
CAGATGAGATGGTCAGACTAAACTGGCTGACGGAATTTATGCCTCTTCCGACCATCAAGCATTTTATCCGTACTCCTG
ATGATGCATGGTTACTCACCACTGCGATCCCCGGAAAAACAGCATTCCAGGTATTAGAAGAATATCCTGATTCAGGTG
AAAATATTGTTGATGCGCTGGCAGTGTTCCTGCGCCGGTTGCATTCGATTCCTGTTTGTAATTGTCCTTTTAACAGCGA
TCGCGTATTTCGTCTCGCTCAGGCGCAATCACGAATGAATAACGGTTTGGTTGATGCGAGTGATTTTGATGACGAGCG
TAATGGCTGGCCTGTTGAACAAGTCTGGAAAGAAATGCATAAACTTTTGCCATTCTCACCGGATTCAGTCGTCACTCA
TGGTGATTTCTCACTTGATAACCTTATTTTTGACGAGGGGAAATTAATAGGTTGTATTGATGTTGGACGAGTCGGAATC
GCAGACCGATACCAGGATCTTGCCATCCTATGGAACTGCCTCGGTGAGTTTTCTCCTTCATTACAGAAACGGCTTTTT
CAAAAATATGGTATTGATAATCCTGATATGAATAAATTGCAGTTTCATTTGATGCTCGATGAGTTTTTCTAATCAGAATTG

GTTAATTGGTTGTAACACTGGCAGAGCATTACGCTGACTTGACGGGACGGCGCAAGCTCATGACCAAAATCCCTTAA
CGTGAGTTACGCGTCGTTCCACTGAGCGTCAGACCCCGTAGAAAAGATCAAAGGATCTTCTTGAGATCCTTTTTTTCT
GCGCGTAATCTGCTGCTTGCAAACAAAAAAACCACCGCTACCAGCGGTGGTTTGTTTGCCGGATCAAGAGCTACCAA
CTCTTTTTCCGAAGGTAACTGGCTTCAGCAGAGCGCAGATACCAAATACTGTTCTTCTAGTGTAGCCGTAGTTAGGCC
ACCACTTCAAGAACTCTGTAGCACCGCCTACATACCTCGCTCTGCTAATCCTGTTACCAGTGGCTGCTGCCAGTGGC
GATAAGTCGTGTCTTACCGGGTTGGACTCAAGACGATAGTTACCGGATAAGGCGCAGCGGTCGGGCTGAACGGGGG
GTTCGTGCACACAGCCCAGCTTGGAGCGAACGACCTACACCGAACTGAGATACCTACAGCGTGAGCTATGAGAAAG
CGCCACGCTTCCCGAAGGGAGAAAGGCGGACAGGTATCCGGTAAGCGGCAGGGTCGGAACAGGAGAGCGCACGA
GGGAGCTTCCAGGGGGAAACGCCTGGTATCTTTATAGTCCTGTCGGGTTTCGCCACCTCTGACTTGAGCGTCGATTT
TTGTGATGCTCGTCAGGGGGGCGGAGCCTATGGAAAAACGCCAGCAACGCGGCCTTTTTACGGTTCCTGGCCTTTT
GCTGGCCTTTTGCTCACATGTTCTTTCCTGCGTTATCCCCTGATTCTGTGGATAACCGTATTACCGCCTTTGAGTGAGC
TGATACCGCTCGCCGCAGCCGAACGACCGAGCGCAGCGAGTCAGTGAGCGAGGAAGCGGAAGAGCGCCCAATAC
GCAAACCGCCTCTCCCCGCGCGTTGGCCGATTCATTAATGCAGCTGGCACGACAGGTTTCCCGACTGGAAAGCGGG
CAGTGAGCGCAACGCAATTAATACGCGTACCGCTAGCCAGGAAGAGTTTGTAGAAACGCAAAAAGGCCATCCGTCAG
GATGGCCTTCTGCTTAGTTTGATGCCTGGCAGTTTATGGCGGGCGTCCTGCCCGCCACCCTCCGGGCCGTTGCTTC
ACAACGTTCAAATCCGCTCCCGGCGGATTTGTCCTACTCAGGAGAGCGTTCACCGACAAACAACAGATAAAACGAAA
GGCCCAGTCTTCCGACTGAGCCTTTCGTTTTATTTGATGCCTGGCAGTTCCCTACTCTCGCGTTAACGCTAGCATGGA
TGTTTTCCCAGTCACGACGTTGTAAAACGACGGCCAGTCTTAAGCTCGGGCCCC

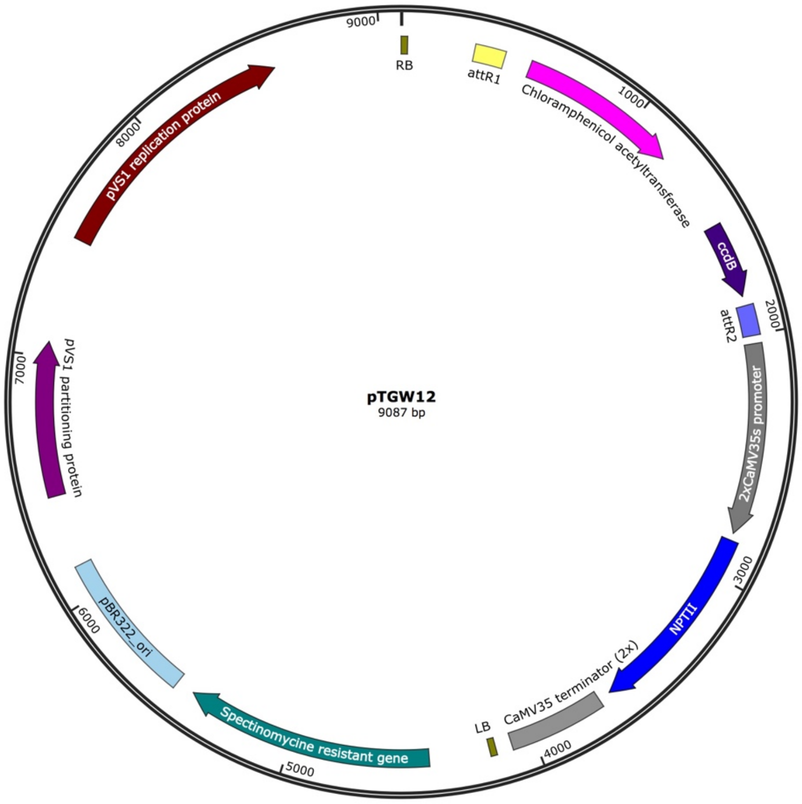

Supplementary Fig. 21. Plasmid vector pTGW12. Intermediate plasmid DNA to construct pMGTiD20.

1..26: RB, T-DNA right border
299..423: attR1
532..1191: Chloramphenicol acetyltransferase
1533..1838: ccdB
1879..2003: attR2
2037..2816: 2xCaMV35s promoter
2847..3644: NPTII
3695..4092: CaMV35 terminator (2x)
4159..4184: LB, T-DNA left border
4431..5441: Spectinomycine resistant gene
5518..6137: pBR322 ori
7490..8563: pVS1 replication protein
6432..7061: pVS1 partitioning protein

>pTGW12
TAAACGCTCTTTTCTCTTAGGTTTACCCGCCAATATATCCTGTCAAACACTGATAGTTTAAACTGAAGGCGGGAAACGA
CAATCTGATCCAAGCTCAAGCTGCTCTAGCATTCGCCATTCAGGCTGCGCAACTGTTGGGAAGGGCGATCGGTGCG
GGCCTCTTCGCTATTACGCCAGCTGGCGAAAGGGGGATGTGCTGCAAGGCGATTAAGTTGGGTAACGCCAGGGTTT
TCCCAGTCACGACGTTGTAAAACGACGGCCAGTGCCAAGCTCCAATTAGGGCCCCGCTAGTGATATCACAAGTTTGT
ACAAAAAAGCTGAACGAGAAACGTAAAATGATATAAATATCAATATATTAAATTAGATTTTGCATAAAAAACAGACTACATA
ATACTGTAAAACACAACATATCCAGTCACTATGGCGGCCGCATTAGGCACCCCAGGCTTTACACTTTATGCTTCCGGCT
CGTATAATGTGTGGATTTTGAGTTAGGATCCGTCGAGATTTTCAGGAGCTAAGGAAGCTAAAATGGAGAAAAAAATCAC
TGGATATACCACCGTTGATATATCCCAATGGCATCGTAAAGAACATTTTGAGGCATTTCAGTCAGTTGCTCAATGTACCT
ATAACCAGACCGTTCAGCTGGATATTACGGCCTTTTTAAAGACCGTAAAGAAAAATAAGCACAAGTTTTATCCGGCCTT
TATTCACATTCTTGCCCGCCTGATGAATGCTCATCCGGAATTCCGTATGGCAATGAAAGACGGTGAGCTGGTGATATG
GGATAGTGTTCACCCTTGTTACACCGTTTTCCATGAGCAAACTGAAACGTTTTCATCGCTCTGGAGTGAATACCACGA
CGATTTCCGGCAGTTTCTACACATATATTCGCAAGATGTGGCGTGTTACGGTGAAAACCTGGCCTATTTCCCTAAAGG
GTTTATTGAGAATATGTTTTTCGTCTCAGCCAATCCCTGGGTGAGTTTCACCAGTTTTGATTTAAACGTGGCCAATATG
GACAACTTCTTCGCCCCCGTTTTCACCATGGGCAAATATTATACGCAAGGCGACAAGGTGCTGATGCCGCTGGCGAT
TCAGGTTCATCATGCCGTTTGTGATGGCTTCCATGTCGGCAGAATGCTTAATGAATTACAACAGTACTGCGATGAGTG
GCAGGGCGGGGCGTAAACGCGTGGATCCGGCTTACTAAAAGCCAGATAACAGTATGCGTATTTGCGCGCTGATTTTT

GCGGTATAAGAATATATACTGATATGTATACCCGAAGTATGTCAAAAAGAGGTaTGCTATGAAGCAGCGTATTACAGTGA
CAGTTGACAGCGACAGCTATCAGTTGCTCAAGGCATATATGATGTCAATATCTCCGGTCTGGTAAGCACAACCATGCA
GAATGAAGCCCGTCGTCTGCGTGCCGAACGCTGGAAAGCGGAAAATCAGGAAGGGATGGCTGAGGTCGCCCGGTT
TATTGAAATGAACGGCTCTTTTGCTGACGAGAACAGGGGCTGGTGAAATGCAGTTTAAGGTTTACACCTATAAAAGAG
AGAGCCGTTATCGTCTGTTTGTGGATGTACAGAGTGATATTATTGACACGCCCGGGCGACGGATGGTGATCCCCCTG
GCCAGTGCACGTCTGCTGTCAGATAAAGTCTCCCGTGAACTTTACCCGGTGGTGCATATCGGGGATGAAAGCTGGC
GCATGATGACCACCGATATGGCCAGTGTGCCGGTCTCCGTTATCGGGGAAGAAGTGGCTGATCTCAGCCACCGCGA
AAATGACATCAAAAACGCCATTAACCTGATGTTCTGGGGAATATAAATGTCAGGCTCCCTTATACACAGCCAGTCTGCA
GGTCGACCATAGTGACTGGATATGTTGTGTTTTACAGTATTATGTAGTCTGTTTTTTATGCAAAATCTAATTTAATATATTG
ATATTTATATCATTTTACGTTTCTCGTTCAGCTTTCTTGTACAAAGTGGTGATATCCCGCGGTGGCGCGCCAAGTTTGC
CAACATGGTGGAGCACGACACTCTCGTCTACTCCAAGAATATCAAAGATACAGTCTCAGAAGACCAAAGGGCTATTGA
GACTTTTCAACAAAGGGTAATATCGGGAAACCTCCTCGGATTCCATTGCCCAGCTATCTGTCACTTCATCAAAAGGAC
AGTAGAAAAGGAAGGTGGCACCTACAAATGCCATCATTGCGATAAAGGAAAGGCTATCGTTCAAGATGCCTCTGCCG
ACAGTGGTCCCAAAGATGGACCCCCACCCACGAGGAGCATCGTGGAAAAAGAAGACGTTCCAACCACGTCTTCAAA
GCAAGTGGATTGATGTGAACATGGTGGAGCACGACACTCTCGTCTACTCCAAGAATATCAAAGATACAGTCTCAGAAG
ACCAAAGGGCTATTGAGACTTTTCAACAAAGGGTAATATCGGGAAACCTCCTCGGATTCCATTGCCCAGCTATCTGTC
ACTTCATCAAAAGGACAGTAGAAAAGGAAGGTGGCACCTACAAATGCCATCATTGCGATAAAGGAAAGGCTATCGTTC
AAGATGCCTCTGCCGACAGTGGTCCCAAAGATGGACCCCCACCCACGAGGAGCATCGTGGAAAAAGAAGACGTTCC
AACCACGTCTTCAAAGCAAGTGGATTGATGTGATATCTCCACTGACGTAAGGGATGACGCACAATCCCACTATCCTTC
GCAAGACCcTTCCTCTATATAAGGAAGTTCATTTCATTTGGAGAGGACACGCTGAAATCACCAGTCTCTCTCTACAAAT
CTATCTCTCTCGAGCTTTCGCAGATCTGTCGATCGACCATGTGGATTGAACAAGATGGATTGCACGCAGGTTCTCCG
GCCGCTTGGGTGGAGAGGCTATTCGGCTATGACTGGGCACAACAGACAATCGGCTGCTCTGATGCCGCCGTGTTtC
GGCTGTCAGCGCAGGGGCGCCCGGTTCTTTTTGTCAAGACCGACCTGTCCGGTGCCCTGAATGAACTCCAGGACG
AGGCAGCGCGGCTATCGTGGCTGGCCACGACGGGCGTTCCTTGCGCAGCTGTGCTCGACGTTGTCACTGAAGCGG
GAAGGGACTGGCTGCTATTGGGCGAAGTGCCGGGGCAGGATCTCCTGTCATCTCACCTTGCTCCTGCCGAGAAAGT
ATCCATCATGGCTGATGCAATGCGGCGGCTGCATACGCTTGATCCGGCTACCTGCCCATTCGACCACCAAGCGAAAC
ATCGCATCGAGCGAGCACGTACTCGGATGGAAGCCGGTCTTGTCGATCAGGATGATCTGGACGAAGAGCATCAGGG
GCTCGCGCCAGCCGAACTGTTCGCCAGGCTCAAGGCGCGCATGCCCGACGGCGAGGATCTCGTCGTGACACATGG
CGATGCCTGCTTGCCGAATATCATGGTGGAAAATGGCCGCTTTTCTGGATTCATCGACTGTGGCCGGCTGGGTGTGG
CGGACCGCTATCAGGACATAGCGTTGGCTACCCGTGATATTGCTGAAGAGCTTGGCGGCGAATGGGCTGACCGCTT
CCTCGTGCTTTACGGTATCGCCGCTCCCGATTCGCAGCGCATCGCCTTCTATCGCCTTCTTGACGAGTTCTTCTGAG
CGGGACTCTGGGGTTCGGATCGATCCTCTAGCTAGAGTCGATCGACAAGCTCGAGTTTCTCCATAATAATGTGTGAGT
AGTTCCCAGATAAGGGAATTAGGGTTCCTATAGGGTTTCGCTCATGTGTTGAGCATATAAGAAACCCTTAGTATGTATTT
GTATTTGTAAAATACTTCTATCAATAAAATTTCTAATTCCTAAAACCAAAATCCAGTACTAAAATCCAGATCCCCCGAATTA
ATTAACGATCGACAAGCTCTATTTTCTCCATAATAATGTGTGAGTAGTTCCCAGATAAGGGAATTAGGGTTCCTATAGGG
TTTCGCTCATGTGTTGAGCATATAAGAAACCCTTAGTATGTATTTGTATTTGTAAAATACTTCTATCAATAAAATTTCTAATT
CCTAAAACCAAAATCCAGTACTAAAATCCAGATCCCCCGAATTAATTCGGCGTTAATTCAGTACATTAAAAACGTCCGC
AATGTGTTATTAAGTTGTCTAAGCGTCAATTTGTTTACACCACAATATATCCTGCCACCAGCCAGCCAACAGCTCCCCG
ACCGGCAGCTCGGCACAAAATCACCACTCGATACAGGCAGCCCATCAGTCCGGGACGGCGTCAGCGGGAGAGCCG
TTGTAAGGCGGCAGACCAGCCAGGACAGAAATGCCTCGACTTCGCTGCTaCCCAAGGTTGCCGGGTGACGCACACC
GTGGAAACGGATGAAGGCACGAACCCAGTGGACATAAGCCTGTTCGGTTCGTAAGCTGTAATGCAAGTAGCGTATGC
GCTCACGCAACTGGTCCAGAACCTTGACCGAACGCAGCGGTGGTAACGGCGCAGTGGCGGTTTTCATGGCTTGTTA
TGACTGTTTTTTTGGGGTACAGTCTATGCCTCGGGCATCCAAGCAGCAAGCGCGTTACGCCGTGGGTCGATGTTTGA
TGTTATGGAGCAGCAACGATGTTACGCAGCAGGGCAGTCGCCCTAAAACAAAGTTAAACATtATGAGGGAAGCGGTG
ATCGCCGAAGTATCGACTCAACTATCAGAGGTAGTTGGCGTCATCGAGCGCCATCTCGAACCGACGTTGCTGGCCGT
ACATTTGTACGGCTCCGCAGTGGATGGCGGCCTGAAGCCACACAGTGATATTGATTTGCTGGTTACGGTGACCGTAA
GGCTTGATGAAACAACGCGGCGAGCTTTGATCAACGACCTTTTGGAAACTTCGGCTTCCCCTGGAGAGAGCGAGAT
TCTCCGCGCTGTAGAAGTCACCATTGTTGTGCACGACGACATCATTCCGTGGCGTTATCCAGCTAAGCGCGAACTGC
AATTTGGAGAATGGCAGCGCAATGACATTCTTGCAGGTATCTTCGAGCCAGCCACGATCGACATTGATCTGGCTATCT
TGCTGACAAAAGCAAGAGAACATAGCGTTGCCTTGGTAGGTCCAGCGGCGGAGGAACTCTTTGATCCGGTTCCTGA
ACAGGATCTATTTGAGGCGCTAAATGAAACCTTAACGCTATGGAACTCGCCGCCCGACTGGGCTGGCGATGAGCGAA
ATGTAGTGCTTACGTTGTCCCGCATTTGGTACAGCGCAGTAACCGGCAAAATCGCGCCGAAGGATGTCGCTGCCGAC
TGGGCAATGGAGCGCCTGCCGGCCCAGTATCAGCCCGTCATACTTGAAGCTAGACAGGCTTATCTTGGACAAGAAGA
AGATCGCTTGGCCTCGCGCGCAGATCAGTTGGAAGAATTTGTCCACTACGTGAAAGGCGAGATCACCAAGGTAGTC
GGCAAATAACCCTCGAGCCACCCATGACCAAAATCCCTTAACGTGAGTTACGCGTCGTTCCACTGAGCGTCAGACCC
CGTAGAAAAGATCAAAGGATCTTCTTGAGATCCTTTTTTTCTGCGCGTAATCTGCTGCTTGCAAACAAAAAAACCACC
GCTACCAGCGGTGGTTTGTTTGCCGGATCAAGAGCTACCAACTCTTTTTCCGAAGGTAACTGGCTTCAGCAGAGCGC
AGATACCAAATACTGTtCTTCTAGTGTAGCCGTAGTTAGGCCACCACTTCAAGAACTCTGTAGCACCGCCTACATACCT
CGCTCTGCTAATCCTGTTACCAGTGGCTGCTGCCAGTGGCGATAAGTCGTGTCTTACCGGGTTGGACTCAAGACGAT
AGTTACCGGATAAGGCGCAGCGGTCGGGCTGAACGGGGGGTTCGTGCACACAGCCCAGCTTGGAGCGAACGACCT

ACACCGAACTGAGATACCTACAGCGTGAGCtaTGAGAAAGCGCCACGCTTCCCGAAGGGAGAAAGGCGGACAGGTA
TCCGGTAAGCGGCAGGGTCGGAACAGGAGAGCGCACGAGGGAGCTTCCAGGGGGAAACGCCTGGTATCTTTATAG
TCCTGTCGGGTTTCGCCACCTCTGACTTGAGCGTCGATTTTTGTGATGCTCGTCAGGGGGGCGGAGCCTATGGAAA
AACGCCAGCAACGCGGCCTTTTTACGGTTCCTGGCCTTTTGCTGGCCTTTTGCTCACATGTTCTTTCCTGCGTTATCC
CCTGATTCTGTGGATAACCGTATTACCGCCTTTGAGTGAGCTGATACCGCTCGCCGCAGCCGAACGACCGAGCGCA
GCGAGTCAGTGAGCGAGGAAGCGGAAGAGCGCCCCTGAAGAAACCGAGCGCCGCCGTCTAAAAAGGTGATGTGTA
TTTGAGTAAAACAGCTTGCGTCATGCGGTCGCTGCGTATATGATGCGATGAGTAAATAAACAAATACGCAAGGGGAAC
GCATGAAGGTTATCGCTGTACTTAACCAGAAAGGCGGGTCAGGCAAGACGACCATCGCAACCCATCTAGCCCGCGC
CCTGCAACTCGCCGGGGCCGATGTTCTGTTAGTCGATTCCGATCCCCAGGGCAGTGCCCGCGATTGGGCGGCCGT
GCGGGAAGATCAACCGCTAACCGTTGTCGGCATCGACCGCCCGACGATTGACCGCGACGTGAAGGCCATCGGCCG
GCGCGACTTCGTAGTGATCGACGGAGCGCCCCAGGCGGCGGACTTGGCTGTGTCCGCGATCAAGGCAGCCGACTT
CGTGCTGATTCCGGTGCAGCCAAGCCCTTACGACATATGGGCCACCGCCGACCTGGTGGAGCTGGTTAAGCAGCGC
ATTGAGGTCACGGATGGAAGGCTACAAGCGGCCTTTGTCGTGTCGCGGGCGATCAAAGGCACGCGCATCGGCGGT
GAGGTTGCCGAGGCGCTGGCCGGGTACGAGCTGCCCATTCTTGAGTCCCGTATCACGCAGCGCGTGAGCTACCCA
GGCACTGCCGCCGCCGGCACAACCGTTCTTGAATCAGAACCCGAGGGCGACGCTGCCCGCGAGGTCCAGGCGCT
GGCCGCTGAAATTAAATCAAAACTCATTTGAGTTAATGAGGTAAAGAGAAAATGAGCAAAAGCACAAACACGCTAAGT
GCCGGCCGTCCGAGCGCACGCAGCAGCAAGGCTGCAACGTTGGCCAGCCTGGCAGACACGCCAGCCATGAAGCG
GGTCAACTTTCAGTTGCCGGCGGAGGATCACACCAAGCTGAAGATGTACGCGGTACGCCAAGGCAAGACCATTACC
GAGCTGCTATCTGAATACATCGCGCAGCTACCAGAGTAAATGAGCAAATGAATAAATGAGTAGATGAATTTTAGCGGCT
AAAGGAGGCGGCATGGAAAATCAAGAACAACCAGGCACCGACGCCGTGGAATGCCCCATGTGTGGAGGAACGGGC
GGTTGGCCAGGCGTAAGCGGCTGGGTTGcCTGCCGGCCCTGCAATGGCACTGGAACCCCCAAGCCCGAGGAATCG
GCGTGAGCGGTCGCAAACCATCCGGCCCGGTACAAATCGGCGCGGCGCTGGGTGATGACCTGGTGGAGAAGTTGA
AGGCCGCGCAGGCCGCCCAGCGGCAACGCATCGAGGCAGAAGCACGCCCCGGTGAATCGTGGCAAGCGGCCGCT
GATCGAATCCGCAAAGAATCCCGGCAACCGCCGGCAGCCGGTGCGCCGTCGATTAGGAAGCCGCCCAAGGGCGAC
GAGCAACCAGATTTTTTCGTTCCGATGCTCTATGACGTGGGCACCCGCGATAGTCGCAGCATCATGGACGTGGCCGT
TTTCCGTCTGTCGAAGCGTGACCGACGAGCTGGCGAGGTGATCCGCTACGAGCTTCCAGACGGGCACGTAGAGGT
TTCCGCAGGGCCGGCCGGCATGGCCAGTGTGTGGGATTACGACCTGGTACTGATGGCGGTTTCCCATCTAACCGAA
TCCATGAACCGATACCGGGAAGGGAAGGGAGACAAGCCCGGCCGCGTGTTCCGTCCACACGTTGCGGACGTACTC
AAGTTCTGCCGGCGAGCCGATGGCGGAAAGCAGAAAGACGACCTGGTAGAAACCTGCATTCGGTTAAACACCACGC
ACGTTGCCATGCAGCGTACGAAGAAGGCCAAGAACGGCCGCCTGGTGACGGTATCCGAGGGTGAAGCCTTGATTAG
CCGCTACAAGATCGTAAAGAGCGAAACCGGGCGGCCGGAGTACATCGAGATCGAGCTAGCTGATTGGATGTACCGC
GAGATCACAGAAGGCAAGAACCCGGACGTGCTGACGGTTCACCCCGATTACTTTTTGATCGATCCCGGCATCGGCC
GTTTTCTCTACCGCCTGGCACGCCGCGCCGCAGGCAAGGCAGAAGCCAGATGGTTGTTCAAGACGATCTACGAACG
CAGTGGCAGCGCCGGAGAGTTCAAGAAGTTCTGTTTCACCGTGCGCAAGCTGATCGGGTCAAATGACCTGCCGGA
GTACGATTTGAAGGAGGAGGCGGGGCAGGCTGGCCCGATCCTAGTCATGCGCTACCGCAACCTGATCGAGGGCGA
AGCATCCGCCGGTTCCTAATGTACGGAGCAGATGCTAGGGCAAATTGCCCTAGCAGGGGAAAAAGGTCGAAAAAGT
CTCTTTCCTGTGGATAGCACGTACATTGGGAACCCAAAGCCGTACATTGGGAACCGGAACCCGTACATTGGGAACCC
AAAGCCGTACATTGGGAACCGGTCACACATGTAAGTGACTGATATAAAAGAGAAAAAAGGCGATTTTTCCGCCTAAAA
CTCTTTAAAACTTATTAAAACTCTTAAAACCCGCCTGGCCTGTGCATAACTGTCTGGCCAGCGCACAGCCGAAGAGCT
GCAAAAAGCGCCTACCCTTCGGTCGCTGCGCTCCCTACGCCCCGCCGCTTCGCGTCGGCCTATCGCGGCCGCTGG
CCGCTCAAAAATGGCTGGCCTACGGCCAGGCAATCTACCAGGGCGCGGACAAGCCGCGCCGTCGCCACTCGACCG
CCGGCGCCCACATCAAGGCACGCACATACAAATGGACGAACGGATAAACCTTTTCACGCCCTTTTAAATATCCGaTTAT
TCTAA

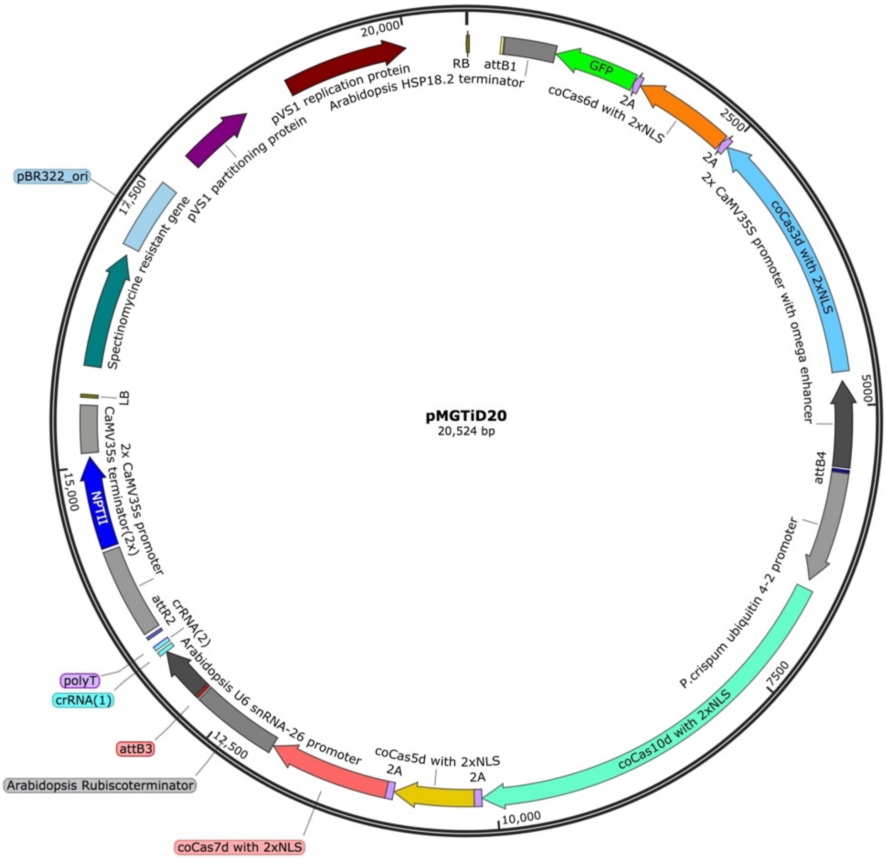

Supplementary Fig. 22. Plasmid vector pMGTiDP20.

1..26: RB, T-DNA right border
299..320: attB1
330..776 (complement): Arabidopsis HSP18.2 terminator
783..1499 (complement): GFP
1500..1562 (complement): 2A self-cleavage peptide
1563..2420 (complement): coCas6d with 2xNLS
2421..2486) (complement): 2A self-cleavage peptide
2487..4703 (complement): coCas3d with 2xNLS
4784..5532 (complement): 2x CaMV35S promoter with omega translational enhancer
5552..5572: attB4
5581..6549: P.crispum ubiquitin 4-2 promoter
6630..10127: coCas10d with 2xNLS
10128..10193: 2A self-cleavage peptide
10194..10892: coCas5d with 2xNLS
10893..10955: 2A self-cleavage peptide
10956..12008: coCas7d with 2xNLS
12015..12757: Arabidopsis Rubisco (small subunit) terminator
12773..12793: attB3
12804..13248: Arabidopsis U6 snRNA-26 promoter
13249..13285: crRNA(1)
13309..13345: crRNA(2)
13346..13353: polyT
13477..14256: 2x CaMV35s promoter
14287..15081: NPTII
15119..15529: CaMV35s terminator(2x)
15596..15621: LB, T-DNA Left border
15868..16878: Spectinomycine resistant gene
16955..17574: pBR322 ori
18927..20000: pVS1 replication protein
17869..18498: pVS1 partitioning protein

>pMGTiD20
TAAACGCTCTTTTCTCTTAGGTTTACCCGCCAATATATCCTGTCAAACACTGATAGTTTAAACTGAAGGCGGGAAACGA
CAATCTGATCCAAGCTCAAGCTGCTCTAGCATTCGCCATTCAGGCTGCGCAACTGTTGGGAAGGGCGATCGGTGCG
GGCCTCTTCGCTATTACGCCAGCTGGCGAAAGGGGGATGTGCTGCAAGGCGATTAAGTTGGGTAACGCCAGGGTTT
TCCCAGTCACGACGTTGTAAAACGACGGCCAGTGCCAAGCTCCAATTAGGGCCCCGCTAGTGATATCACAAGTTTGT
ACAAAAAAGCAGGCGGGGCCCGTCTCACGTTACAATTAGTACTAAAATAAACGTAAACCGAACTTAATCTCCTCATTAT
GTGCCATGTTTTAAATCATTTGTTTTGCGGTTATTACGCTTACTTTTTTCTACATCTTTATTTTATAAAATAGATCATGAAAT
AGCATGATGAAGGGAGGTTTATAGGTCCCTCGCCTCGTTTTACTCTATGAGCCCATCTTATCTTTAATCATATTCCATAG
TCCATACCATAGCACATACAGTAGTTATATGCTGCAGAAGAGATCCAACAAAACATTCACAATGGATTATAGAAACATTT
GTTTATTCATTATAATGAAATCTTACATTCATTTAATATTAGAAAAAGCCACAAATTCATAACACAACAAGCCAAGAAAAAA
ACACAAACTTAAGCACACAAACTTTTTATTTGACAcaccaaatatttcatcttcatcttcatATGAGCTCTTACTTGTACAGCTCGTCC
ATGCCGAGAGTGATCCCGGCGGCGGTCACGAACTCCAGCAGGACCATGTGATCGCGCTTCTCGTTGGGGTCTTTGC
TCAGGGCGGACTGGGTGCTCAGGTAGTGGTTGTCGGGCAGCAGCACGGGGCCGTCGCCGATGGGGGTGTTCTGC
TGGTAGTGGTCGGCGAGCTGCACGCTGCCGTCCTCGATGTTGTGGCGGATCTTGAAGTTCACCTTGATGCCGTTCT
TCTGCTTGTCGGCCATGATATAGACGTTGTGGCTGTTGTAGTTGTACTCCAGCTTGTGCCCCAGGATGTTGCCGTCCT
CCTTGAAGTCGATGCCCTTCAGCTCGATGCGGTTCACCAGGGTGTCGCCCTCGAACTTCACCTCGGCGCGGGTCTT
GTAGTTGCCGTCGTCCTTGAAGAAGATGGTGCGCTCCTGGACGTAGCCTTCGGGCATGGCGGACTTGAAGAAGTCG
TGCTGCTTCATGTGGTCGGGGTAGCGGCTGAAGCACTGCACGCCGTAGGTCAGGGTGGTCACGAGGGTGGGCCAG
GGCACGGGCAGCTTGCCGGTGGTGCAGATGAACTTCAGGGTCAGCTTGCCGTAGGTGGCATCGCCCTCGCCCTCG
CCGGACACGCTGAACTTGTGGCCGTTTACGTCGCCGTCCAGCTCGACCAGGATGGGCACCACCCCGGTGAACAGC
TCCTCGCCCTTGCTCACTGGCCCGGGATTCTCCTCAACGTCACCGCATGTTAGCAGACTTCCTCTGCCCTCTCCGGA
TCCTCTCTTGTCCTTAGAGATTCTTCTAGTCATTCCCATTCCCATAGTAGTCTTTCTTCCCACTCCAGCATAAAGAGCG
AAGTCAGCAAGAGCGTTGATAGCCTTGATCTTAGCTGGCTCCACCTCTCCAAGAAGTCTATAGTGGATCTCTCCCACG
CATCCGATAGACTGGTTCTTATAAGCCTCGTCAGCAAGCTTAGTCTGGATGTCGAAGAAAGATGGGAAGATAGACTCA
AGAGCGATAGAGTCGAATGGGATTCCAGAATATCTGTTCCATCTTCCAAGAAGAGAGTTGAACACAAGCTCTCTAGTT
GGAAGAGCAGAGTCGAACTTTCCCTGTCTGAAAGTCACTGGAGTAGCGAAAAGGAAGTCGAACTCTCTGTTAGTCTC
AGAAGCGTTCTCATAAAGATACTGATAAGTGCAAGAGTTAGCCCAAGGCTGCACAGACTGTGGAGTAGCAAGCACAG
AAGTGATCACAAGGTCAGCAGATCCAAGGTGCCAAGGCTGCTTTGGGTTAAGGTTAAGCCAAAGAGAAGTAAGCTTT
CCGAAAAGTCTGTCGTCAAGAAGAGAGATTCTCCACCAGCATGGAGTCTCAGAAGCGATCTCTCTCTCGTGTCTCCA
CTGTGGAGAGTTGATAGTCTTTCCTCCAGACTGCATCTGAAGTGGAGAAAGAGTGAAAGCCTTGTCAGCCTCAGCAG
TGTGAAGGATGTTTCCAAGCTCCTGGTCCACAGAAGACACAAGAGTAAGGAAAAGAGCGTGAAGGTGTCTTCCAGTA
AGGAAGTTTGGATAGATTGGAGATCTTGGAGTAAGGTTAAGCACAAGAGAATATGGTCCAGAAACCTTCCTTTTCTTTT
TCGGGTCGAATTCAGGGCCAGGGTTTTCCTCCACGTCGCCGCAGGTCAGCAGGCTGCCTCTGCCCTCAGAGCCGC
AGATCCACACCTCTCCTCCCTTTCCCTTGAATCTGTGAGCAAGAGTGTCAAGAAGAAGAGCAGCCTGTCCGATAGCG
ATAGAATATGGAGAAAGTGGAGAGTGAAGAGAAGACTCGTCAGAGATTGGATAGATTCCGAAGTGCATTGGAAGCTG
AAGTCTCTGTCTCACAGCAGCCACTGGATAAGCGAACACGAAAGACACAAGAGCAAGATCTCTAAGCTCTCTTGAGA
TTCTGTCAAGCCAAGGGTTGTCTGGCTGCCACACTCCGATTCCAGTAAGCACCTGCACCTTCCAAGCAGAAGCGATC
TCTCCAAGATCTCCGTCATAAGTGAATCTCCAGTTAAGTCTCTCCTCTCTATATCCCTTAAGGTTAAGGAAAGCAAGGC
AATAGTCGAATCTTCTTCTAGCGATTGGAGTCTTAGTAGCCTCGATCTGTCTCTTGAACTCTCCCTTTCTCCACACCTC
CACCTCAAGGTTTCCAAGGATAGATGGAAGGTCATAAGTCTTGAATCTGTCGCACTCCTCTGGCTCAGTAGAGTCATA
AAGTCCGCAAAGAAGTGGAGAAGATCCTCTGAAAGAAGAAGCGTCCTCGAAGATTGGGTTTCCGTTCTTTCCAGAAA
GCTCCTTCCACTCGTCAGCCCATCCCTTCACTCTTCCAGCCACTCTCTTAAGAGAAGTGTCGAACACCTCCTCGCAC
TCCTGCTGGAACTTCTGTCTTGACTCTCCATACTGAGACTTGATCTTTGGAGATCCAAGGTTCCACCAAAGCTGGAAA
GACTGCACAGCTCCCCATCTCTGATAATATCCCTCGAAGTTGTTGATGTGTCTATAGTTAGACTTGATAGCCTCCTGAA
GCTGGATTCTGTCATATCTCTCTCCCACCTGAAGTGGAGCGTCCTTCTTCTCGAAAAGTCTCTCAAGGAAGAACTTTG
GCACAAGAGCGATAGCAGTGAAGTTAGTGAACTTCACAGCAGTTCCCTTTCTGTCATATCCAGAGTGTCTTCCAAGTC
TTCCGAATCTCTGGATGAAGTTTCCAGCGTCAGAAGACTCGAAGATAAGGAAGTTGATCTTGAAGTCCACTCCCACG
TCGATAGTAGAAGTTCCGATCACAAGATCTCTGTTAAGAGAAGCAAGTTTCTCTCTAGTTCCAGAAAGTCCAGTGTTCT
CTCCCACAGTAAGTCCGATAGTAGCAAGAAGCTCTCTGAAGATTGGAAGAAGTCTCTTCACAGAAGCGATAGAGTTAA
GGATGATAGCTCCCTTAGATCCTGGATATCTCTTGAAATAGTCAAGGATTCTCTCCTTGTTCTCCTTAAGCCAGTTCTC
AGAAGTCTGGAAAGAAGATGGAAGTGGGATGAAAGAAAGCTCGATCTCAGAAGTCACCTGTCTCCATCCCTGCTGAG
CAAGAGAGTTAGCCTCCTCTGGAGTGTCTGGGAATCTATACTTTCCCTCTCTCACTGGGTCGATAGATCTATATCTGAA
TCCAGCCTTGTCAAGTCTCTTAAGAAGTCCCTCGTCTGGAGTAGCAGAAAGGAAAAGATATCTCTTTCCTCTGTTAGC
TCTTCTGATAAGAAGCATAGTGTTGATGATAGAAGCCACCTGTGGAGTTCCGAACACGTGGAACTCGTCGAAAAGGA
AAAGGTCGAAGTGCTTGTCGATTCTGTTCCAAAGCTGGTCTGGGTTGTCATATGGAGTAAGATAAGCAGCTCTGTGAA
GATAGTGGAAGATGTCTGGGTTAGTAAGAAGGATCTCAGACTGCTTAGATCTAGTCTCAAGAGCGATAGCCTTAGTCT
TTCCGTCTCTCTCAGCATAAAGCTCAAGCTCTGGTCCAGTAAGAAGGTTCACTCTTGGCTGGTCAGTTGGCTGGAAT
CTCTGGATATAAGCCTCGATCTGTCCTCTCTGATCTCTAGCAAGCTCGTTAGTTGGATAAAGTCCCATGATTGGGAAAT
ATCCCTGAAGAGTTCTAAGATAAGCAGCAAGAGACTTTCCGTCTCCAGTCATAGCAGTGTTGAAGATCACGTCGATGT

CTGGGTCGTTAAGAGCCTTCCAAGTCTCCACCTGGTGCCAAGAAAGTCTCCATCCCTGTGGCACCTTGATTCCGTCT
GGGATCTCGTCAGCTGGGCAAGAATACACTGGCTTAAGAGTCACCTGATAGTTTCCTCCAGAAACCTTGCGCTTCTT
CTTAGGGTCTACCTTCCGCTTCTTCTTTGGGTCCATGGTGCTTGTAATTGTAAATAGTAATTGTAATGTTGTTTGTTGTT
TGTTGTTGTTGGTAATTGTTGTAAAAATACTCTAGACCGGCCTCTCCAAATGAAATGAACTTCCTTATATAGAGGAAGG
GTCTTGCGAAGGATAGTGGGATTGTGCGTCATCCCTTACGTCAGTGGAGATATCACATCAATCCACTTGCTTTGAAGA
CGTGGTTGGAACGTCTTCTTTTTCCACGATGCTCCTCGTGGGTGGGGGTCCATCTTTGGGACCACTGTCGGCAGAG
GCATCTTGAACGATAGCCTTTCCTTTATCGCAATGATGGCATTTGTAGGTGCCACCTTCCTTTTCTACTGTCCTTTTGAT
GAAGTGACAGATAGCTGGGCAATGGAATCCGAGGAGGTTTCCCGATATTACCCTTTGTTGAAAAGTCTCAATAGCCCT
TTGGTCTTCTGAGACTGTATCTTTGATATTCTTGGAGTAGACGAGAGTGTCGTGCTCCACCATGTTCACATCAATCCAC
TTGCTTTGAAGACGTGGTTGGAACGTCTTCTTTTTCCACGATGCTCCTCGTGGGTGGGGGTCCATCTTTGGGACCAC
TGTCGGCAGAGGCATCTTGAACGATAGCCTTTCCTTTATCGCAATGATGGCATTTGTAGGTGCCACCTTCCTTTTCTAC
TGTCCTTTTGATGAAGTGACAGATAGCTGGGCAATGGAATCCGAGGAGGTTTCCCGATATTACCCTTTGTTGAAAAGT
CTCAATAGCCCTTTGGTCTTCTGAGACTGTATCTTTGATATTCTTGGAGTAGACGAGAGTGTCGTGCTCCACCATGTTG
GCTTGGCGCGCCATTAACACCCAACTTTTCTATACAAAGTTGGGCGCGCCAAAAATTACGGATATGAATATAGGCATAT
CCGTATCCGAATTATCCGTTTGACAGCTAGCAACGATTGTACAATTGCTTCTTTAAAAAAGGAAGAAAGAAAGAAAGAA
AAGAATCAACATCAGCGTTAACAAACGGCCCCGTTACGGCCCAAACGGTCATATAGAGTAACGGCGTTAAGCGTTGA
AAGACTCCTATCGAAATACGTAACCGCAAACGTGTCATAGTCAGATCCCCTCTTCCTTCACCGCCTCAAACACAAAAAT
AATCTTCTACAGCCTATATATACAACCCCCCCTTCTATCTCTCCTTTCTCACAATTCATCATCTTTCTTTCTCTACCCCCA
ATTTTAAGAAATCCTCTCTTCTCCTCTTCATTTTCAAGGTAAATCTCTCTCTCTCTCTCTCTCTCTGTTATTCCTTGTTTT
AATTAGGTATGTATTATTGCTAGTTTGTTAATCTGCTTATCTTATGTATGCCTTATGTGAATATCTTTATCTTGTTCATCTCA
TCCGTTTAGAAGCTATAAATTTGTTGATTTGACTGTGTATCTACACGTGGTTATGTTTATATCTAATCAGATATGAATTTCT
TCATATTGTTGCGTTTGTGTGTACCAATCCGAAATCGTTGATTTTTTTCATTTAATCGTGTAGCTAATTGTACGTATACATA
TGGATCTACGTATCAATTGTTCATCTGTTTGTGTTTGTATGTATACAGATCTGAAAACATCACTTCTCTCATCTGATTGTG
TTGTTACATACATAGATATAGATCTGTTATATCATTTTTTTATTAATTGTGTATATATATATGTGCATAGATCTGGATTACATG
ATTGTGATTATTTACATGATTTTGTTATTTACGTATGTATATATGTAGATCTGGACTTTTTGGAGTTGTTGACTTGATTGTAT
TTGTGTGTGTATATGTGTGTTCTGATCTTGATATGTTATGTATGTGCATCTAGAGTATTTTTACAACAATTACCAACAACA
ACAAACAACAAACAACATTACAATTACTATTTACAATTACAAGCACCATGGACCCTAAGAAAAAGAGAAAGGTTTCTGG
ACCAAAGAAGCAGAAGAAGCTTGAGGAGACTGGACAGCTTAACCTTTTCGACAACACTACTGAGATCGACGACGAG
GACCTTGACTTCGAGTTCGAGGACATCGACCTTGAGTCTCTTGTGTCTGAGGACCTTGGAATCACTGAGTCTGTGTC
TGACAGAAGAGTGGAGACTGTGAGACAGCTTCTTACTCTTAAGCTTCTTAGAGAGGCTATCAGAGCTGAGAACCCAG
ACGACAGAGTGATGGCTGACTTCGCTGAGATGGTGCTTCCAAACCTTCTTAGACTTGCTATCGGAGTGACTGCTAAG
GGAGGAAACTTCATCGAGGCTGTGGACAGAGGAAGAGAGCTTAGAAACAAGCCAAAGGCTAAGAGAGACAACGCTG
GAGATCAGTCTCTTAACACTCACCTTCTTAACGGACTTTTCCCAGCTAACCTTATCGAGAAGAGACTTCAGAAGCTTAA
CACTACTGTGAGAAGAATCATCAAGGAGTTCGAGAGAAGACTTGCTATCGCTGGATTCCTTGTGCACGACTTCGAGA
AGTTCTCTTATGACAGATTCCCATCTATGTCTGAGAGATATATCCAGATCCAGAGAGACTTCATCCAGGACCCATTCAA
GAACCAGGACCCAAGAAAGCTTTCAAGAGAGGAGCACAGAGAGATCCTTCAGGTGCTTATCCCAGAGCTTGGACTT
GACAGATTCCTTTTCCCAGACAACCCAGAGAGATGGCTTGAGTATCTTGACGACCTTCTTTATATCGCTAAGAACACTC
AGAGAAGAAACGACACTGACCTTAACACTTCTGAGGACGGACTTAACGTGAGACTTAACGACAGAGTGATCGAGTCT
CTTTGCGACCTTGCTTGCCTTGCTGACAGACTTGCTTCTATCATCAAGCACCCACACGACGCTGAGAAGGCTTCTCT
TCAGGACCTTCTTTATTCTCTTTCTGACGGAGAGCTTAAGTTCACTTATCACTCTATCGCTGAGAACAGAGGAGTGCTT
ACTAACGTGCTTAACAACGCTGTGATGGAGGCTCACCAGGAGCTTGACTATCAGCCACTTCTTTATCTTCCAACTGGA
GTGGTGTATATCGCTCCAAAGAACGCTCCAGAGGTGTCTCTTGAGACTCTTCCAAACAGAGTGGTGGACACTATCAA
GTCTCTTTGCTCTGGAGAGCTTCAGAGAAAGCAGACTGGATTCGGAAGAGACGGAAAGGGtATGAAGTATGCTGACT
ATTATTCTCAGTTCTTCGACGACGCTGGACTTATGAGAGCTGCTCTTAACGCTACTCTTAGAATCCTTGGAGACAACAA
GGCTTCTGTGGCTAGATCTAGAGGAGAGAACCTTATCAAGTTCCAGCAGCAGGGAGTGCTTCCAACTGACTATGACT
TCCACTGCGAGGACGACATCAGAATCGACAGACTTGCTGAGTTCGGAGACGTGGTGACTAGAAAGATCTGGGGAGA
CAGACTTGAGAAGATCGAGCAGGCTAGAAAGCTTCAGAAGAACCTTCCAGCTCCACCAGACCTTGACCTTATCTCTG
AGATCGCTCACTATTGGAACCTTGAGAACTATCTTCCACAGATCAGAGCTATCAAGAGAATCAACGAGTCTCTTAAGG
AGCTTAAGCTTAAGGGAAACACTGGAGGAGTGCCATATGAGTGGTATTATCTTGCTGCTCAGTATCTTAAGCAGCACC
CAGGAATCGAGGACATCAGACCAGTGGCTGAGGACCTTATCGCTTTCCTTGCTGCTAAGATCGCTGCTATCGTGGCT
GGATATAACCTTCCAGACGGATGGGAGGACCTTAGAGAGTGGGTGAACCAGGTGGTGCAGCTTCCAGGAAGAGAGC
TTGCTCACTCTATCGAGACTTTCCAGAAGGAGCTTAACCACTATAACGCTGCTAAGAAGCAGGGAAGAGGAAGACAG
CTTCTTTGCTCTATCTCTCACTCTCCATATTCTGTGTCTGAGCAGATGGAGTCTGCTGTGCTTTTCACTCCACAGGTGT
ATACTAACAAGCAGATGCTTGCTGGATCTAACGCTAAGAGAAACATCTCTTCTATCGCTGGAACTGAGATGATGCTTAG
ACAGATCCTTATGAACCAGACTCAGGCTGTGGGAAAGAGATTCGAGGACGGAAAGTATAGATATCTTTATTTCTATCCA
ACTTATTATTTCACTCCAGAGACTAACTCTTTCCTTCAGAAGGCTTATGCTAACATCGCTCAGACTAGATTCGACTCTTC
TATCAAGCTTCACTTCGTGGACAAGAACCTTGTGGCTAACTTCGACAGAACTAGATATCAGTCTGTGGACTCTTTCCTT
ATCGACGAGAAGCTTAGACAGAAGAAGGAGACTATCAACGAGGAGGAGGACGGAAAGAAGGACAGAACTTTCAAGC
TTTCTTATCCAGAGGACAAGCCACTTACTTTCTATTTCATGGCTCTTCCACCAGGAAGAAACCCAACTGACACTGAGT
CTTGGGTAATGCCAGCTTGGCTTGGACTTGCTTTCCCAATGATCCTTGACGTGAAGACTGTGGTGTCTGAGTCTCCA

ATCCCACCATATAGAGACGGAGCTGAGTTCGAGGAGACTGTGTTCCTTGACTCTGCTCCACAGGCTATCAGATCTCTT
ACTAGATGCGACAGATTCAGACTTGACAGAGTGCTTAACCCTTGGCAGGACAACGACGGAAAGAAGTATTCTGCTCC
ACTTAACACTCTTACTGCTGCTTATTCTATCCACCTTGACGTGAACTCTAAGCAGGGAAAGACTGGATATGACCCAAAC
TGGGGAAAGCTTACTGAGCTTGCTATCAACCTTGAGACTTCTCCACTTTATGTGTTCCACTATTTCAAGCAGTGGAAG
AGAGGAAAGGACGCTGACATCCCATCTGCTAACAGAATCGCTCTTTATCTTTATGACTTCTATCCATGCTTCGACCCAT
ATGTGCAGGCTAACAGAACTAACCTTACTATCGACATGACTGCTGAGTCTCCACTTAACCACCCAAAGAACCTTACTG
AGCTTTATAGACAGTTCTATAGAGCTAAGTCTTCTAAGGGAAAGCCAATCAAGGCTAACGCTATCCTTAAGCCAATCGA
CGAGGCTGCTGACATCATCCTTAAGGCTGACAAGGCTATCTCTGACGACCTTACTTCTCTTGTGGCTGCTAGACTTTT
CAAGCTTATGGACAGAGTGAGATCTCAGACTGCTGAGGGAAGATATGTGATCAAGGAGAGAGATCAGGAGAGAGAG
AAGATCCTTGACTTCGCTAAGTATTTCGTGAAGAACGTGTTCGAGGAGTCTTTCGAGTCTGACAGAGCTAGACTTGCT
GGAAGACAGCTTAACATCATCAGAGACACTTGCGAGTTCCTTTATAGACTTGAGATGGACAAGGAGAGAAGACAGAG
ACAGGTGCAGCCACTTGACACTTCTAACTCTTCTTCTGAGGAGGAGGAGGGGTCTGAGGGACGCGGCTCCCTGCTC
ACCTGTGGAGATGTGGAAGAGAACCCAGGCCCCGAATTCGACCCGAAAAAGAAAAGGAAGGTTTCTGGAGTGCACA
TCTATTCTTGCCAGCTTGAGCTTCACGACTCTCTTTATTATGCTACTAGAGAGATCGGAAGACTTTATGAGTCTGAGCC
AGTGATCCACAACTATGCTCTTTGCTATGCTCTTGGACTTGTGAACTCTGACTCTTATAGATATTTCTGCTCTGAGCAG
ATCCCACAGTATCAGGAGCACCTTAACCCACTTAACGAGGAGAAGATCTATGTGACTCCAGCTAGAGCTATCGCTCAC
ACTGCTGTGCTTAACACTTGGAAGTATGCTAACAACAACTATCACGTGGAGATGGAGAAGACTCAGAAGAACATCCCA
TCTTTCGGAAGAGCTAAGGAGATCGCTCCAGAGTCTATCTTCGAGTGCTTCATCATCTCTCACCACCCACTTCAGCTT
CCAAAGTGGATCAGACTTGGAAAGTGGATGTCTAAGGCTGAGGTGAAGCTTACTGAGCTTTCTCTTTCTAAGCAGAA
GGAGGACCTTTTCATCTATCCATATCCACTTAACCCACTTGACGTGATGTTCACTCACCAGGTTATCGGATATGACGTG
ATCAACATGCCACCAGTGTCTCTTATCAGAAACGTGAGAATGAGAGGAGAGTATTATCAGATCTCTGACAGACCAGAC
CTTAAGATCCCAGCTAGACTTTCTTATCACTTCGGAGGATCCGGAGAGGGCAGAGGAAGTCTGCTAACATGCGGTGA
CGTTGAGGAGAATCCCGGGCCAGACCCAAAGAAGAAGCGGAAGGTAGACCCTAAGAAGAAGCGCAAGGTTTCTGG
AACTTTCCTTACTTCTGTGGACGCTAAGTTCTTCCACTCTGAGATCCCATATAAGCCAATGGGAAAGTATGTGCACTTC
CTTACTATCAGAGTGACTGAGTCTTATCCACTTTTCCAGACTGACGGAGAGCTTAACAAGGCTAGAGTGAGAGCTGGA
ATCGACTCTAAGAAGACTATCTCAAGACTTTCTATGTTCAAGAGAAAGCAGTCTACTCCAGAGAGACTTGTGGGAAGA
GAGCTTCTTAGAAACTATGGACTTATCACTGCTGAGGAGTGCGAGTATAACGTGAAGTTCGCTATGAACAACGCTGAC
TGCATCATCTATGGATTCGCTATCGGAGACTCTGGATCTGAGAAGTCTAAGGTGGTGGTGGACACTGCTTTCTCTATC
ACTCCATTCGACGAGTCTCACGAGTCTTTCACTCTTAACGCTCCATATGAGAACGGAACTATGGCTTCTAAGGGAGAG
AACAACACTAAGGTGGGAGAGGTGACTTCAAGAATCAACCAGCAGGACCACATCAGACCACAGGTGTTCTTCCCATC
TATCGTGACTCTTAAGGACCCAACTGAGGCTTCTTTCCTTTATGTGTTCAACAACATCCTTAGAACTAGACACTATGGT
GCTCAGACTACTAGAACTGGAAGAGTGAGAAACGAGCTTATCGGAGTGATCTTCGCTGACGGAGAGATCGTGTCTAA
CCTTAGATGGACTCAGGCTATCTATGACAGACTTCCAGACGAGGTGCTTCACTCTATCGACCCACTTGACGAGGACC
TTGTGATGGAGAAGGCTACTGAGGCTATCCAGGCTCTTATGGCTGAGGAGTTCATCGTGCACACTGACTTCATCGGA
GAGAACTTCCAGCCACTTCTTACTGAGGTGAAGACTCTTACTGGAACTGAGGCTGGAATCCTTTCTGTGCTTGACCA
GGCTAACAAGGAGTCTAAGAAGTATTTCGAGCAGTATATCGAGAAGAAGAAGGCTGAGAAGAAGTAAGAGCTCTCCC
CTTTCTGGAATATTCAGCGTTGATTATTCTGGAACCCATTTCTATGTGGTCAATGCAAATTTAAGAAATTATTTGCCGACT
TAAAAGTTGAGGAACTATTGTTGAAAGTGAAAATGTTATTCCTATCAGTTTCTCTATAATTATAGTTATCATTTCATTTCAT
TTTTGCCCTTAGCTCTTTGAAATCTTATTTTTCGTTTAGCTCCTTTAAACAACATTGTGGCTCCTTTAAATTATCCTCATA
ATTCTTGCTTTTGACTCCCTAGACTAACCAATAAACTCTAATAAAAAAAGAAAAACTTGCTACATTGTTTTAAGAAATTTT
GCACATGAAAGCAATCAATCAAACCTCGATATTTAAAGAAAAGTTTATGTAAGGGAGTGTAACCATTTTTCAGATGACAT
AGCCATTGGAGATTTGGAAAAGGTAGTATATACAGAAAATTCAAATGCATCTTTTAAAATTATAAACATAAAACATGTATT
CCAAACCCCTAAGTGGGATATTAAGTCAAGAAATAGCATTACATAAAGCAAGGATCGACAAAGACTGAAATTTGTCAAG
CATGAAGTTACTAAATTTTGGAAATTTTGTTTACGTTAAATTTGATCATTGGTTATGCATTAAAAGCTGAAACAGTTATTTA
GAAGGAAAAGTAGTTAACGTTCCCAAAAAGTTCTAAATAAAACTAGATTCCAAATGATAATGAAAACGTCGCATCTACTA
TATGTTTGGAATCCTGCAGGGATTAAACAACTTTGTATAATAAAGTTGGGCCTGCAGGCTTCGTTGAACAACGGAAACT
CGACTTGCCTTCCGCACAATACATCATTTCTTCTTAGCTTTTTTTCTTCTTCTTCGTTCATACAGTTTTTTTTTGTTTATC
AGCTTACATTTTCTTGAACCGTAGCTTTCGTTTTCTTCTTTTTAACTTTCCATTCGGAGTTTTTGTATCTTGTTTCATAGT
TTGTCCCAGGATTAGAATGATTAGGCATCGAACCTTCAAGAATTTGATTGAATAAAACATCTTCATTCTTAAGATATGAA
GATAATCTTCAAAAGGCCCCTGGGAATCTGAAAGAAGAGAAGCAGGCCCATTTATATGGGAAAGAACAATAGTATTTCT
TATATAGGCCCATTTAAGTTGAAAACAATCTTCAAAAGTCCCACATCGCTTAGATAAGAAAACGAAGCTGAGTTTATATA
CAGCTAGAGTCGAAGTAGTGATTGTTCCAATTAATCTTAAGCCCTATTAGGGATTGAAACGGAGACCCTCAATTGTCGG
TCTCGTTCCAATTAATCTTAAGCCCTATTAGGGATTGAAACTTTTTTTTGCAAAATTTTCCAGATCGATTTCTTCTTCCTC
TGTTCTTCGGCGTTCAATTTCTTACGTATTAATACCCAGCTTTCTTGTACAAAGTGGTGATATCCCGCGGTGGCGCGCC
AAGTTTGCCAACATGGTGGAGCACGACACTCTCGTCTACTCCAAGAATATCAAAGATACAGTCTCAGAAGACCAAAGG
GCTATTGAGACTTTTCAACAAAGGGTAATATCGGGAAACCTCCTCGGATTCCATTGCCCAGCTATCTGTCACTTCATCA
AAAGGACAGTAGAAAAGGAAGGTGGCACCTACAAATGCCATCATTGCGATAAAGGAAAGGCTATCGTTCAAGATGCCT
CTGCCGACAGTGGTCCCAAAGATGGACCCCCACCCACGAGGAGCATCGTGGAAAAAGAAGACGTTCCAACCACGT
CTTCAAAGCAAGTGGATTGATGTGAACATGGTGGAGCACGACACTCTCGTCTACTCCAAGAATATCAAAGATACAGTC
TCAGAAGACCAAAGGGCTATTGAGACTTTTCAACAAAGGGTAATATCGGGAAACCTCCTCGGATTCCATTGCCCAGCT

ATCTGTCACTTCATCAAAAGGACAGTAGAAAAGGAAGGTGGCACCTACAAATGCCATCATTGCGATAAAGGAAAGGCT
ATCGTTCAAGATGCCTCTGCCGACAGTGGTCCCAAAGATGGACCCCCACCCACGAGGAGCATCGTGGAAAAAGAAG
ACGTTCCAACCACGTCTTCAAAGCAAGTGGATTGATGTGATATCTCCACTGACGTAAGGGATGACGCACAATCCCACT
ATCCTTCGCAAGACCcTTCCTCTATATAAGGAAGTTCATTTCATTTGGAGAGGACACGCTGAAATCACCAGTCTCTCTC
TACAAATCTATCTCTCTCGAGCTTTCGCAGATCTGTCGATCGACCATGATTGAACAAGATGGATTGCACGCAGGTTCT
CCGGCCGCTTGGGTGGAGAGGCTATTCGGCTATGACTGGGCACAACAGACAATCGGCTGCTCTGATGCCGCCGTGT
TtCGGCTGTCAGCGCAGGGGCGCCCGGTTCTTTTTGTCAAGACCGACCTGTCCGGTGCCCTGAATGAACTCCAGGA
CGAGGCAGCGCGGCTATCGTGGCTGGCCACGACGGGCGTTCCTTGCGCAGCTGTGCTCGACGTTGTCACTGAAGC
GGGAAGGGACTGGCTGCTATTGGGCGAAGTGCCGGGGCAGGATCTCCTGTCATCTCACCTTGCTCCTGCCGAGAAA
GTATCCATCATGGCTGATGCAATGCGGCGGCTGCATACGCTTGATCCGGCTACCTGCCCATTCGACCACCAAGCGAA
ACATCGCATCGAGCGAGCACGTACTCGGATGGAAGCCGGTCTTGTCGATCAGGATGATCTGGACGAAGAGCATCAG
GGGCTCGCGCCAGCCGAACTGTTCGCCAGGCTCAAGGCGCGCATGCCCGACGGCGAGGATCTCGTCGTGACACAT
GGCGATGCCTGCTTGCCGAATATCATGGTGGAAAATGGCCGCTTTTCTGGATTCATCGACTGTGGCCGGCTGGGTGT
GGCGGACCGCTATCAGGACATAGCGTTGGCTACCCGTGATATTGCTGAAGAGCTTGGCGGCGAATGGGCTGACCGC
TTCCTCGTGCTTTACGGTATCGCCGCTCCCGATTCGCAGCGCATCGCCTTCTATCGCCTTCTTGACGAGTTCTTCTGA
GCGGGACTCTGGGGTTCGGATCGATCCTCTAGCTAGAGTCGATCGACAAGCTCGAGTTTCTCCATAATAATGTGTGA
GTAGTTCCCAGATAAGGGAATTAGGGTTCCTATAGGGTTTCGCTCATGTGTTGAGCATATAAGAAACCCTTAGTATGTAT
TTGTATTTGTAAAATACTTCTATCAATAAAATTTCTAATTCCTAAAACCAAAATCCAGTACTAAAATCCAGATCCCCCGAAT
TAATTAACGATCGACAAGCTCTATTTTCTCCATAATAATGTGTGAGTAGTTCCCAGATAAGGGAATTAGGGTTCCTATAG
GGTTTCGCTCATGTGTTGAGCATATAAGAAACCCTTAGTATGTATTTGTATTTGTAAAATACTTCTATCAATAAAATTTCTA
ATTCCTAAAACCAAAATCCAGTACTAAAATCCAGATCCCCCGAATTAATTCGGCGTTAATTCAGTACATTAAAAACGTCC
GCAATGTGTTATTAAGTTGTCTAAGCGTCAATTTGTTTACACCACAATATATCCTGCCACCAGCCAGCCAACAGCTCCC
CGACCGGCAGCTCGGCACAAAATCACCACTCGATACAGGCAGCCCATCAGTCCGGGACGGCGTCAGCGGGAGAGC
CGTTGTAAGGCGGCAGACCAGCCAGGACAGAAATGCCTCGACTTCGCTGCTaCCCAAGGTTGCCGGGTGACGCACA
CCGTGGAAACGGATGAAGGCACGAACCCAGTGGACATAAGCCTGTTCGGTTCGTAAGCTGTAATGCAAGTAGCGTAT
GCGCTCACGCAACTGGTCCAGAACCTTGACCGAACGCAGCGGTGGTAACGGCGCAGTGGCGGTTTTCATGGCTTG
TTATGACTGTTTTTTTGGGGTACAGTCTATGCCTCGGGCATCCAAGCAGCAAGCGCGTTACGCCGTGGGTCGATGTT
TGATGTTATGGAGCAGCAACGATGTTACGCAGCAGGGCAGTCGCCCTAAAACAAAGTTAAACATtATGAGGGAAGCGG
TGATCGCCGAAGTATCGACTCAACTATCAGAGGTAGTTGGCGTCATCGAGCGCCATCTCGAACCGACGTTGCTGGCC
GTACATTTGTACGGCTCCGCAGTGGATGGCGGCCTGAAGCCACACAGTGATATTGATTTGCTGGTTACGGTGACCGT
AAGGCTTGATGAAACAACGCGGCGAGCTTTGATCAACGACCTTTTGGAAACTTCGGCTTCCCCTGGAGAGAGCGAG
ATTCTCCGCGCTGTAGAAGTCACCATTGTTGTGCACGACGACATCATTCCGTGGCGTTATCCAGCTAAGCGCGAACT
GCAATTTGGAGAATGGCAGCGCAATGACATTCTTGCAGGTATCTTCGAGCCAGCCACGATCGACATTGATCTGGCTAT
CTTGCTGACAAAAGCAAGAGAACATAGCGTTGCCTTGGTAGGTCCAGCGGCGGAGGAACTCTTTGATCCGGTTCCT
GAACAGGATCTATTTGAGGCGCTAAATGAAACCTTAACGCTATGGAACTCGCCGCCCGACTGGGCTGGCGATGAGCG
AAATGTAGTGCTTACGTTGTCCCGCATTTGGTACAGCGCAGTAACCGGCAAAATCGCGCCGAAGGATGTCGCTGCCG
ACTGGGCAATGGAGCGCCTGCCGGCCCAGTATCAGCCCGTCATACTTGAAGCTAGACAGGCTTATCTTGGACAAGAA
GAAGATCGCTTGGCCTCGCGCGCAGATCAGTTGGAAGAATTTGTCCACTACGTGAAAGGCGAGATCACCAAGGTAGT
CGGCAAATAACCCTCGAGCCACCCATGACCAAAATCCCTTAACGTGAGTTACGCGTCGTTCCACTGAGCGTCAGACC
CCGTAGAAAAGATCAAAGGATCTTCTTGAGATCCTTTTTTTCTGCGCGTAATCTGCTGCTTGCAAACAAAAAAACCACC
GCTACCAGCGGTGGTTTGTTTGCCGGATCAAGAGCTACCAACTCTTTTTCCGAAGGTAACTGGCTTCAGCAGAGCGC
AGATACCAAATACTGTtCTTCTAGTGTAGCCGTAGTTAGGCCACCACTTCAAGAACTCTGTAGCACCGCCTACATACCT
CGCTCTGCTAATCCTGTTACCAGTGGCTGCTGCCAGTGGCGATAAGTCGTGTCTTACCGGGTTGGACTCAAGACGAT
AGTTACCGGATAAGGCGCAGCGGTCGGGCTGAACGGGGGGTTCGTGCACACAGCCCAGCTTGGAGCGAACGACCT
ACACCGAACTGAGATACCTACAGCGTGAGCtaTGAGAAAGCGCCACGCTTCCCGAAGGGAGAAAGGCGGACAGGTA
TCCGGTAAGCGGCAGGGTCGGAACAGGAGAGCGCACGAGGGAGCTTCCAGGGGGAAACGCCTGGTATCTTTATAG
TCCTGTCGGGTTTCGCCACCTCTGACTTGAGCGTCGATTTTTGTGATGCTCGTCAGGGGGGCGGAGCCTATGGAAA
AACGCCAGCAACGCGGCCTTTTTACGGTTCCTGGCCTTTTGCTGGCCTTTTGCTCACATGTTCTTTCCTGCGTTATCC
CCTGATTCTGTGGATAACCGTATTACCGCCTTTGAGTGAGCTGATACCGCTCGCCGCAGCCGAACGACCGAGCGCA
GCGAGTCAGTGAGCGAGGAAGCGGAAGAGCGCCCCTGAAGAAACCGAGCGCCGCCGTCTAAAAAGGTGATGTGTA
TTTGAGTAAAACAGCTTGCGTCATGCGGTCGCTGCGTATATGATGCGATGAGTAAATAAACAAATACGCAAGGGGAAC
GCATGAAGGTTATCGCTGTACTTAACCAGAAAGGCGGGTCAGGCAAGACGACCATCGCAACCCATCTAGCCCGCGC
CCTGCAACTCGCCGGGGCCGATGTTCTGTTAGTCGATTCCGATCCCCAGGGCAGTGCCCGCGATTGGGCGGCCGT
GCGGGAAGATCAACCGCTAACCGTTGTCGGCATCGACCGCCCGACGATTGACCGCGACGTGAAGGCCATCGGCCG
GCGCGACTTCGTAGTGATCGACGGAGCGCCCCAGGCGGCGGACTTGGCTGTGTCCGCGATCAAGGCAGCCGACTT
CGTGCTGATTCCGGTGCAGCCAAGCCCTTACGACATATGGGCCACCGCCGACCTGGTGGAGCTGGTTAAGCAGCGC
ATTGAGGTCACGGATGGAAGGCTACAAGCGGCCTTTGTCGTGTCGCGGGCGATCAAAGGCACGCGCATCGGCGGT
GAGGTTGCCGAGGCGCTGGCCGGGTACGAGCTGCCCATTCTTGAGTCCCGTATCACGCAGCGCGTGAGCTACCCA
GGCACTGCCGCCGCCGGCACAACCGTTCTTGAATCAGAACCCGAGGGCGACGCTGCCCGCGAGGTCCAGGCGCT
GGCCGCTGAAATTAAATCAAAACTCATTTGAGTTAATGAGGTAAAGAGAAAATGAGCAAAAGCACAAACACGCTAAGT

GCCGGCCGTCCGAGCGCACGCAGCAGCAAGGCTGCAACGTTGGCCAGCCTGGCAGACACGCCAGCCATGAAGCG
GGTCAACTTTCAGTTGCCGGCGGAGGATCACACCAAGCTGAAGATGTACGCGGTACGCCAAGGCAAGACCATTACC
GAGCTGCTATCTGAATACATCGCGCAGCTACCAGAGTAAATGAGCAAATGAATAAATGAGTAGATGAATTTTAGCGGCT
AAAGGAGGCGGCATGGAAAATCAAGAACAACCAGGCACCGACGCCGTGGAATGCCCCATGTGTGGAGGAACGGGC
GGTTGGCCAGGCGTAAGCGGCTGGGTTGcCTGCCGGCCCTGCAATGGCACTGGAACCCCCAAGCCCGAGGAATCG
GCGTGAGCGGTCGCAAACCATCCGGCCCGGTACAAATCGGCGCGGCGCTGGGTGATGACCTGGTGGAGAAGTTGA
AGGCCGCGCAGGCCGCCCAGCGGCAACGCATCGAGGCAGAAGCACGCCCCGGTGAATCGTGGCAAGCGGCCGCT
GATCGAATCCGCAAAGAATCCCGGCAACCGCCGGCAGCCGGTGCGCCGTCGATTAGGAAGCCGCCCAAGGGCGAC
GAGCAACCAGATTTTTTCGTTCCGATGCTCTATGACGTGGGCACCCGCGATAGTCGCAGCATCATGGACGTGGCCGT
TTTCCGTCTGTCGAAGCGTGACCGACGAGCTGGCGAGGTGATCCGCTACGAGCTTCCAGACGGGCACGTAGAGGT
TTCCGCAGGGCCGGCCGGCATGGCCAGTGTGTGGGATTACGACCTGGTACTGATGGCGGTTTCCCATCTAACCGAA
TCCATGAACCGATACCGGGAAGGGAAGGGAGACAAGCCCGGCCGCGTGTTCCGTCCACACGTTGCGGACGTACTC
AAGTTCTGCCGGCGAGCCGATGGCGGAAAGCAGAAAGACGACCTGGTAGAAACCTGCATTCGGTTAAACACCACGC
ACGTTGCCATGCAGCGTACGAAGAAGGCCAAGAACGGCCGCCTGGTGACGGTATCCGAGGGTGAAGCCTTGATTAG
CCGCTACAAGATCGTAAAGAGCGAAACCGGGCGGCCGGAGTACATCGAGATCGAGCTAGCTGATTGGATGTACCGC
GAGATCACAGAAGGCAAGAACCCGGACGTGCTGACGGTTCACCCCGATTACTTTTTGATCGATCCCGGCATCGGCC
GTTTTCTCTACCGCCTGGCACGCCGCGCCGCAGGCAAGGCAGAAGCCAGATGGTTGTTCAAGACGATCTACGAACG
CAGTGGCAGCGCCGGAGAGTTCAAGAAGTTCTGTTTCACCGTGCGCAAGCTGATCGGGTCAAATGACCTGCCGGA
GTACGATTTGAAGGAGGAGGCGGGGCAGGCTGGCCCGATCCTAGTCATGCGCTACCGCAACCTGATCGAGGGCGA
AGCATCCGCCGGTTCCTAATGTACGGAGCAGATGCTAGGGCAAATTGCCCTAGCAGGGGAAAAAGGTCGAAAAAGT
CTCTTTCCTGTGGATAGCACGTACATTGGGAACCCAAAGCCGTACATTGGGAACCGGAACCCGTACATTGGGAACCC
AAAGCCGTACATTGGGAACCGGTCACACATGTAAGTGACTGATATAAAAGAGAAAAAAGGCGATTTTTCCGCCTAAAA
CTCTTTAAAACTTATTAAAACTCTTAAAACCCGCCTGGCCTGTGCATAACTGTCTGGCCAGCGCACAGCCGAAGAGCT
GCAAAAAGCGCCTACCCTTCGGTCGCTGCGCTCCCTACGCCCCGCCGCTTCGCGTCGGCCTATCGCGGCCGCTGG
CCGCTCAAAAATGGCTGGCCTACGGCCAGGCAATCTACCAGGGCGCGGACAAGCCGCGCCGTCGCCACTCGACCG
CCGGCGCCCACATCAAGGCACGCACATACAAATGGACGAACGGATAAACCTTTTCACGCCCTTTTAAATATCCGaTTAT
TCTAA

Supplementary Tables

Supplementary Table 1. The list of TiD gRNAs target sequences.

gRNA name*	PAM	Sequence, 5'-3'	target gene	Locus	Description
SlIAA9_GTC_gRNA1(+)	GTC	TACCTGGATCTCAGTCTCCCGAAAGAGGTGAGGAG	SlIAA9 (Solyc04g076850)	Chr4: 61847384-61847422 (SL3.0)	"gRNA1(+)" or "232-267" used in Fig. 1f.
SlIAA9_GTT_gRNA 5A(-)	GTT	GAAGAAGCCAGTAGAGTTACATTACTCTGGCCCTC	SlIAA9 (Solyc04g076850)	Chr4: 61847188-61847226 (SL3.0)	"gRNA 5A(-)" or "33-68" used in Fig. 1f. Two gRNAs, GTT_gRNA 5A(-) and GTT_gRNA 5B(+), were used for the multiplex mutagenesis and described as "GTT+GTT_gRNA5(-)(+)" (Fig. 3) in further analysis. TiD CRISPR crRNA (5'-GTTCCAATTAATCTTAAGCCCTATTAGGGATTGAAAC-3') was inserted between two gRNAs,GTT_gRNA5-A(–) and GTT_gRNA5-B(+), in the multiplex vector, in which two gRNAs can be expressed separately in plant cells.
SlIAA9_GTT_gRNA 5B(+)	GTT	CGTCGGTGGACAGCTGTAATATTTCCACCTCATCA	SlIAA9 (Solyc04g076850)	Chr4: 61847294-61847332 (SL3.0)	
SlIAA9_GTC_gRNA24-59 (+)	GTT	GGGGAGGAGGAGGGCCAGAGTAATGTAACTCTACT	SlIAA9 (Solyc04g076850)	Chr4: 61847176-61847214 (SL3.0)	"24-59" used in Fig. 1f.
SlIAA9_GTC_gRNA82-117(-)	GTT	TCGCTCTTTAAGAGCTGATCCTTTTATGCATATGC	SlIAA9 (Solyc04g076850)	Chr4: 61847237-61847275 (SL3.0)	"82-117" used in Fig. 1f.
SlIAA9_GTT_gRNA177-212(-)	GTT	GCCTTGAGATTTAATCCACACCCATTATTGTCCTC	SlIAA9 (Solyc04g076850)	Chr4: 61847332-61847370 (SL3.0)	"177-212" used in Fig. 1f.
SlIAA9_GTT_gRNA294-329(+)	GTT	GATGAGAAGCTGCTCTTCCCCTTGCACCCTTCCAA	SlIAA9 (Solyc04g076850)	Chr4: 61847446-61847484 (SL3.0)	"294-329" used in Fig. 1f.
SlRIN_GTC_4003-4238(+)	GTC	AATTGGATTCATCATTGAGGCAAATTAGGTCAACA	SlRIN (Solyc05g012020)	Chr5: 5226882-5226920 (SL3.0)	
AAVS1_GTC_70-107(+)	GTC	cctagtggccccactgtggggtggaggggacagat	*Hs* AAVS1	Chr19: 55115760-55115794	"AAVS70-107" was used in Fig. 1d, 1e.
AAVS1_GTC_159-196(+)	GTC	ccagctcggggacacaggatccctggaggcagcaa	*Hs* AAVS1	Chr19: 55115849-55115883	"AAVS159-196" was used in Fig. 1d, 1e.
OsNADK2_GTC_91-125(+)	GTC	GGGAGGTGGTGGTGGAGGCCGGCGGCGGCGGGGCG	OsNADK2(Os11g0191400)	Chr11: 4625322-4625356	
OsNADK2_GTT_2196-2230(-)	GTT	GCAGATGCACACATATTTCCATCAACATGATCTGA	OsNADK2(Os11g0191400)	Chr11: 4627461-4627427	
OsNADK2_GTA_1980-2014(-)	GTA	ATACCTGAAGCAACTGATAGAATATAATCATTATC	OsNADK2(Os11g0191400)	Chr11: 4627245-4627211	
OsNADK2_GTA_3860-3894(-)	GTA	TTTCTCTAATAACTTCTGAATCTTACCTTTGTGAT	OsNADK2(Os11g0191400)	Chr11: 4629125-4629091	

Supplementary Table 2. The list of target DNA fragments used in the nanoLuc assay.

	Sequence, 5'-3'
IAA9_24-59 (+)<br>IAA9_33-68 (-)<br>IAA9_82-117 (-)<br>IAA9_177-212 (-)<br>IAA9_232-267 (+)<br>IAA9_294-329 (+)	GGGGAGGAGGAGGGCCAGAGTAATGTAACTCTACTGGCTTCTTCAACTTCCTTAGGAAGCATATGCATAAAAGGATCAGCTCTTAAAGAGCGAA<br>ACTATATGGGTCTATCTGATTGTTCGTCGGTGGACAGCTGTAATATTTCCACCTCATCAGAGGACAATAATGGGTGTGGATTAAATCTCAAGGCA<br>ACGGAGCTCAGGCTCGGTCTACCTGGATCTCAGTCTCCCGAAAGAGGTGAGGAGACTTGCCCTGTGAGCTCGACAAAGGTTGATGAGAAGCT<br>GCTCTTCCCCTTGCACCCTTCCAAAGATTCTGCTTTCTCGGTATCGCAGAAAACAGTAGTTAGTGGCAAC
OsNADK2_GTC_91-125(+)	CGCCGAGAACACAACCTCCGCGACAGCGACGCGCTCCCTCCGCCGCCGCCGCCGCCGCCGCTGCCACGCGAAAGGCTCCGTCCTCGTCC<br>TCGCCGGCTCCGCGGGACCCTGCTTGATGCTCGCCGTCTGCGCGCGGCACGGGCCCGCGAAGCTTCCGCCGCCGCCGCCGCCGCTCGCC<br>GGGGAGCGGGCGGCCGCATGGGTCGTCGGGAGGTGGTGGTGGAGGCCGGCGGCGGCGGGGCGGCGCGGCGTCGTCGCCGCGCGGGCGT<br>CGTTCTTCAGCTCGCGGATCGGGCTCGACTCCCAGGTTCGTCGGTTCGATCGCCCGCCGCTCGTGAGCATTTCGTCGAATATCTGCGGGGCG<br>GTCCTTGCCGCGCTGCTTTCTGGGGGTCGTTGCAGCGTAGTAGTTCTTGATTTTTAGTTATCAATTTCCGTTGCTTTCTTGTACATATGGCTGATG<br>AGTGATGACCTGTGCTTTTGTTCACCTTGAGATCATCTGGTACTCTGGCTTGGG
OsNADK2_GTT_2196-2230(-)<br>OsNADK2_GTA_1980-2014(-)	ACGGTCGAATAGCTTGTTGGTCTCAAGGAGAAAACAAAGTCTCAGTGCTGAACAAAATGGAGCCATTGACTATGAAGCAGCCGAATTTAAAGTT<br>CTAAAAAGTTCGAATGGAGCATCGTTCGATAATGATTATATTCTATCAGTTGCTTCAGGTATTACTAATGGAAAACCATCCAACAATGGAGCCTC<br>CACATCTGTTGAGGACAGGGAAATGGAAACCTCAGTTGTAACAGTTGATCCTAGGACATCTGATACCAGCAATTCTAATGGCAATGCTCCACTT<br>GGATCACAAAAATCTGCTGAAAGGAACGGTTCTCTTTATGTGGAGAGAGAAAAATCAGATCATGTTGATGGAAATATGTGTGCATCTGCAACTGG<br>TGTTGTTAGACTTCAGTCAAGAAGAAAAGCAGAGATGTTTCTAGTACGCACTGATGGATTTTCCTGTACAAGAGAAAAAGTAACTGAATCATCTC<br>TGGCTTTTACGCATCCTAGCACCCAGCAGCAGATGCTTATGTGGAAATCTCCTCCAAAGACTG
OsNADK2_GTA_3860-3894(-)	CCTCCTTTCAGACAACACTTTATCATGTTAACTTGTTTATTTGTCAAATCTAAGCATGCTATCATTTTGTTTACCTCAGTTCGAAGGTTTCAGACAA<br>GACTTGAGGGCTGTCATCCATGGGAACAATACACTTGGAGTTTATATAACCCTTAGAATGCGTCTACGATGTGAGATCTTTCGCAATGGAAAAG<br>CAATGCCTGGAAAAGTATTTGATGTGCTAAATGAAGTTGTTGTTGATCGGGGTTCTAATCCTTACCTGTCCAAAATTGAATGCTATGAGCATAACC<br>ATTTAATCACAAAGGTAAGATTCAGAAGTTATTAGAGAAATACATTTCTCTTAGCATATTTTTGCTTAGTGGCTGTGTTCATCATGGTGTTTTTCTTT<br>CTGTTATTTCTGTTTGGAGATTTAATGGTTGGAGTCATGAAGCTAGGCCCTTTTCCCAATTGTATTATGCTTGACAATGTTTAGGCAGCAGCTGCA<br>TTGAAATAGAAAAGTCACAA

Supplementary Table 3. Primers for short-range PCRs to detect small in/dels

Oligor name	Sequence, 5'-3'	Purpose	Description
SlIAA9_F27-52	GGAGGAGGAGGGCCAGAGTAATGTAA	Cel-1 assay and PCR-RFLP of the target region for *SlIAA9*	PCR primers used in Fig. 2 and Supplementary Figs. 2 & 3.
SlIAA9_R375-348	GTTGCCACTAACTACTGTTTTCTGCGAT	Cel-1 assay and PCR-RFLP of the target region for *SlIAA9*	
SLiCE_IAA9_F27-52	ACCCGGGGGCGCGCCGGATCGGAGGAGGAGGGCCAGAGTAATGTAA	Cloning of the target region for *SlIAA9* by SLiCE method	
SLiCE_IAA9_R375-348	TCTAGACTTAATTAAGGATCGTTGCCACTAACTACTGTTTTCTGCGAT	Cloning of the target region for *SlIAA9* by SLiCE method	
MiSeq500_TiD-IAA9-1_Fw	ACACTCTTTCCCTACACGACGCTCTTCCGATCTTCTACTGGCTTCTTCAACTTCCTT	1st PCR primer for the on-target in next-generation sequencing	PCR primers used in Fig. 2f and Supplementary Fig. 3c.
MiSeq500_TiD-IAA9-1_Rv	GTGACTGGAGTTCAGACGTGTGCTCTTCCGATCTTTGCCACTAACTACTGTTTTCTGC	1st PCR primer for the on-target in next-generation sequencing	
TiD_IAA9-1_off-target1_Fw	ACACTCTTTCCCTACACGACGCTCTTCCGATCTTTCACTCTTAAGCCCTCTATTGG	1st PCR primer for the off-target1 in next-generation sequencing	PCR primers used in Fig. 5c.
TiD_IAA9-1_off-target1_Rv	GTGACTGGAGTTCAGACGTGTGCTCTTCCGATCTATGGCACCCTTTGAATCACT	1st PCR primer for the off-target1 in next-generation sequencing	
TiD_IAA9-1_off-target2_Fw	ACACTCTTTCCCTACACGACGCTCTTCCGATCTTGTTTTCGGCCTATTTCAGG	1st PCR primer for the off-target1 in next-generation sequencing	
TiD_IAA9-1_off-target2_Rv	GTGACTGGAGTTCAGACGTGTGCTCTTCCGATCTCCACACGCAACCAATTAATGTA	1st PCR primer for the off-target1 in next-generation sequencing	
TiD_IAA9-1_off-target7_Fw	ACACTCTTTCCCTACACGACGCTCTTCCGATCTTGGAACGTCGTACACACTTAGC	1st PCR primer for the off-target1 in next-generation sequencing	
TiD_IAA9-1_off-target7_Rv	GTGACTGGAGTTCAGACGTGTGCTCTTCCGATCTAGCCTATTTTGGGAATTATCG	1st PCR primer for the off-target1 in next-generation sequencing	
TiD_IAA9-5B_off-target1_Fw	GATCAACTATGTGCTTTTAAATTCC	Cel-1 assay of the target region	PCR primers used in Supplementary Fig. 5c.
TiD_IAA9-5B_off-target1_Rv	TCTCAAAACATTATGAGGACCTCTGT	Cel-1 assay of the target region	
TiD_IAA9-5B_off-target2_Fw	GTCATTATTCTTTGATATAGGACCTGAA	Cel-1 assay of the target region	
TiD_IAA9-5B_off-target2_Rv	ATTCATTCTCAGCACAAATATATGAAAC	Cel-1 assay of the target region	
TiD_RIN_off-target1_Fw	AGATGAGACCTCGTAAAATAAATTAAGG	Cel-1 assay of the target region	
TiD_RIN_off-target1_Rv	ATATATATAAATTGTTGAACCCCGTTG	Cel-1 assay of the target region	
TiD_RIN_off-target2_Fw	CAATTCATATCTACATGATCCTACTACG	Cel-1 assay of the target region	
TiD_RIN_off-target2_Rv	AAAATATCCATTTAGACTTCATACTCCA	Cel-1 assay of the target region	

Supplementary Table 4. Primers for long-range PCRs to detect large deletions

Oligor name	Sequence, 5'-3'	Purpose	Description
SlIAA9-1_1.4k-F1	TTGTTACTGTCTGCCAAATGGAGG	Long PCR, Fig. 3 (1st PCR)	Data in the upper panel of Fig.3 were detected only by the 1st PCR.<br>Data in the lower panels of Fig.3 and Fig 4 were detected by nested PCR.
SlIAA9-1_3.8k-R1	CTTAATCAAACGACAAACGTTGGGTC	Long PCR, Fig. 3 (1st PCR)	
SlIAA9-1_1.3k-F2	TCTTCCTTCTTCTCTTACTGCTGC	Long PCR, Fig. 3 (nested PCR)	
SlIAA9-1_3.7k-R2	AGACAAACTCCAATATCAAACGGTTTTT	Long PCR, Fig. 3 (nestedPCR)	
SlRIN-3.2k-F1	TCCTTTAAAAGTTGGCCATACTAACTAC	Long PCR, Fig. 4 (1st PCR)	
SlRIN-3.1k-R1	AATCGTATTTGTTCTACTGTCTGC	Long PCR, Fig. 4 (1st PCR)	
SlRIN-3.2k-F2	TTATAACTCATATCATTGGCGGAACTTG	Long PCR, Fig. 4 (nested PCR)	
SlRIN-3k-R2	ATCTGTGTATCGAGGATTGTTTC	Long PCR, Fig. 4 (nested PCR)	
SlRIN-off target1_3k-F1	ACTTTGGACACTCTCCGTGAAAT	Long PCR, Fig. 5c left (1st PCR)	Data in Fig. 5d were detected by nested PCR.
SlRIN-off target1_3k-R1	GGGACACAGGCAGAAGAAATAAC	Long PCR, Fig. 5c left (1st PCR)	
SlRIN-off target1_3k-F2	ACCCAAAGTTCGAACATGAGACT	Long PCR, Fig. 5c left (nested PCR)	
SlRIN-off target1_3k-R2	AGCTTTCAACTAAACACACG	Long PCR, Fig. 5c left (nested PCR)	
SlRIN-off target2_3k-F1	CTTGTTCATCATTGTGAGTCGGG	Long PCR, Fig. 5c right (1st PCR)	
SlRIN-off target2_3k-R1	AGCCTTTTGGAAAACATTTGGGT	Long PCR, Fig. 5c right (1st PCR)	
SlRIN-off target2_3k-F2	AATGGCCACAAGAAATGTGGAAG	Long PCR, Fig. 5c right (nested PCR)	
SlRIN-off target2_3k-R2	TAGTTTCAAGGCATCTTGGTCGT	Long PCR, Fig. 5c right (nested PCR)	
SlIAA9-1-off target1_5k-F1	TCCCACCACACTTAGCACAACTAG	Long PCR, Supplementary Fig. 5a (1st PCR)	Data in supplementary Fig. 5a were detected by nested PCR.<br>(SlIAA9-1-off target1; 3K+3K,<br>SlIAA9-1-off target2; 3K+3K,<br>SlIAA9-1-off target3; 2.6K+3.6K,<br>SlIAA9-5-off target1; 2.3K+3.7K,<br>SlIAA9-5-off target2; 4K+4.2K)
SlIAA9-1-off target1_3k-R1	CATGTTAGTCCCTAGTTTCAC	Long PCR, Supplementary Fig. 5a (1st PCR)	
SlIAA9-1-off target1_3k-F2	TGCCAAAAATAGAATCCCATGAACC	Long PCR, Supplementary Fig. 5a (nested PCR)	
SlIAA9-1-off target1_3k-R2	AAAGAGTAGGCACATGAAGAGG	Long PCR, Supplementary Fig. 5a (nested PCR)	
SlIAA9-1-off target2_3k-F1	TTATTACCTTGCCGGTGTAAGAGAG	Long PCR, Supplementary Fig. 5a (1st PCR)	
SlIAA9-1-off target2_4.3k-R1	AAATGGGCCACTGAATTGCTAG	Long PCR, Supplementary Fig. 5a (1st PCR)	
SlIAA9-1-off target2_3k-F2	AATAACGAAATTGGGACTGGTGAAC	Long PCR, Supplementary Fig. 5a (nested PCR)	
SlIAA9-1-off target2_3k-R2	TTTTTCTCTTCCAATTGCTCTGCAT	Long PCR, Supplementary Fig. 5a (nested PCR)	
SlIAA9-1-off target3_3.2k-F1	ATCATACCTCAAGCTAGAATTGAGCTTCC	Long PCR, Supplementary Fig. 5a (1st PCR)	
SlIAA9-1-off target3_4k-R1	CGTCTTTATGCCAAGTTCTCATAGTGAT	Long PCR, Supplementary Fig. 5a (1st PCR)	
SlIAA9-1-off target3_2.6k-F2	ATGTTATGGTGGAAAGGACGCTAT	Long PCR, Supplementary Fig. 5a (nested PCR)	
SlIAA9-1-off target3_3.6k-R2	GGTTTGGATTGTGTATTGATGCGA	Long PCR, Supplementary Fig. 5a (nested PCR)	
SlIAA9-5B-off target1_2.4k-F1	CTAGGCGTAGTGCCCCGAGAACA	Long PCR, Supplementary Fig. 5a (1st PCR)	
SlIAA9-5B-off target1_3.9k-R1	GGATGAATATCCAGTTTCTAGGGCTAGG	Long PCR, Supplementary Fig. 5a (1st PCR)	
SlIAA9-5B-off target1_2.3k-F2	GTCGACCCTTTAGTTTCGGTTTAGC	Long PCR, Supplementary Fig. 5a (nested PCR)	
SlIAA9-5B-off target1_3.7k-R2	CCAATTTACTTCTGTCGGTCCTTCTC	Long PCR, Supplementary Fig. 5a (nested PCR)	
SlIAA9-5B-off target2_4.1k-F1	GTTCTGCTTAGAGGAGTTTCCTGATG	Long PCR, Supplementary Fig. 5a (1st PCR)	
SlIAA9-5B-off target2_4.3k-R1	GGGTCCATTGGTGAAGACTGTG	Long PCR, Supplementary Fig. 5a (1st PCR)	
SlIAA9-5B-off target2_4k-F2	CATACTTGGTTTCCATTTGCCAGG	Long PCR, Supplementary Fig. 5a (nested PCR)	
SlIAA9-5B-off target2_4.2k-R2	CACGGATGCTTCATGTGCATTG	Long PCR, Supplementary Fig. 5a (nested PCR)	

Supplementary Table 5. Off-target sequences.

target	locus	mismatch	sequences	Description
SlIAA9 GTC_gRNA1(+)	on-target	0	GTCTACCTGGATCTCAGTCTCCCGAAAGAGGTGAGGAG	*GTT_gRNA5-A(-) has no potential off-target sites in the tomato genome.
off-target1	ch09_42659576	9	GTCTAC**t**TG**at**T**a**T**t**AG**a**CTCC**t**GAAAG**g**GGTGA**t**GAG	
off-target2	ch02_35115698	10	GTCTA**ta**TGGATCT**a**AGTC**gg**CCGA**gc**GA**t**GT**c**AGG**g**G	
off-target3	ch08_46023555	11	GTCTAC**a**TGGATCT**a**AGTC**gg**CC**a**A**gt**GA**t**GT**t**AGG**ga**	
SlIAA9 GTT+GTT_gRNA5-(–)(+)				
GTT_gRNA5-B(+)	on-target	0	GTTCGTCGGTGGACAGCTGTAATATTTCCACCTCATCA	
off-target1	ch01_77004266	11	GTC**t**GTCG**t**TG**tt**CA**tt**T**ca**AAT**g**TTTCCA**tt**TCATCA	
off-target2	ch11_4520196	11	GTTCGTC**a**GTGGACA**ca**T**t**T**c**ATATTTC**attt**TC**tc**CA	
GTT_gRNA5-A(-)	on-target	0	GTTGAAGAAGCCAGTAGAGTTACATTACTCTGGCCCTC	
-*	-*	-*	-*	
SlRIN GTC_4003–4238(+)	on-target	0	GTCAATTGGATTCATCATTGAGGCAAATTAGGTCAACA	
off-target1	ch05_230678	6	GTCAA**c**TGGATTCATC**t**TTGAGGC**t**AAT**a**AG**a**TCAA**g**A	
off-target2	ch04_39422124	8	GTA**tt**TTGGATTCAT**a**ATTGAGG**a**AAA**c**TAGGT**gg**AC**t**	
