# Supplementary material for: Genome editing in plants using CRISPR type I-D nuclease
Source: Commun Biol. 2020 Nov 6;3:648. doi: 10.1038/s42003-020-01366-6 (PMC7648086; doi:10.1038/s42003-020-01366-6)
Supplement: Supplementary file 3 — Description of Additional Supplementary Files [file 42003_2020_1366_MOESM3_ESM.pdf]

Description of Additional Supplementary Files

File Name: Supplementary Data 1

Description: Source data for Fig. 1 and Supplementary Fig. 1.

File Name: Supplementary Data 2

Description: Source sequence data for Supplementary Data Table 1 of Fig. 1.
